# Is genetic liability to ADHD and ASD causally linked to educational attainment?

**DOI:** 10.1093/ije/dyab107

**Published:** 2021-06-07

**Authors:** Christina Dardani, Lucy Riglin, Beate Leppert, Eleanor Sanderson, Dheeraj Rai, Laura D Howe, George Davey Smith, Kate Tilling, Anita Thapar, Neil M Davies, Emma Anderson, Evie Stergiakouli

**Affiliations:** 1 Centre of Academic Mental Health, Bristol Medical School, University of Bristol, Bristol, UK; 2 Division of Psychological Medicine and Clinical Neurosciences, MRC Centre for Neuropsychiatric Genetics and Genomics, Cardiff University, Cardiff, UK; 3 Medical Research Council Integrative Epidemiology Unit, Bristol Medical School, University of Bristol, Bristol, UK; 4 Population Health Sciences, Bristol Medical School, University of Bristol, Bristol, UK; 5 K.G. Jebsen Center for Genetic Epidemiology, Department of Public Health and Nursing, Norwegian University of Science and Technology, Trondheim, Norway

**Keywords:** ADHD, ASD, education, cognitive ability, Mendelian randomization, multivariable

## Abstract

**Background:**

The association patterns of attention deficit hyperactivity disorder (ADHD) and autism spectrum disorder (ASD) with educational attainment (EA) are complex; children with ADHD and ASD are at risk of poor academic outcomes, and parental EA has been associated with risk of ADHD/ASD in the offspring. Little is known on the causal links between ADHD, ASD, EA and the potential contribution of cognitive ability.

**Methods:**

Using the latest genome-wide association studies (GWAS) summary data on ADHD, ASD and EA, we applied two-sample Mendelian randomization (MR) to assess the effects of genetic liability to ADHD and ASD on EA. Reverse direction analyses were additionally performed. Multivariable MR was performed to estimate any effects independent of cognitive ability.

**Results:**

Genetic liability to ADHD had a negative effect on EA, independently of cognitive ability (_MVMR_IVW: -1.7 months of education per doubling of genetic liability to ADHD; 95% CI: -2.8 to -0.7), whereas genetic liability to ASD a positive effect (_MVMR_IVW: 30 days per doubling of the genetic liability to ASD; 95% CI: 2 to 53). Reverse direction analyses suggested that genetic liability to higher EA had an effect on lower risk of ADHD, independently of cognitive ability (_MVMR_IVW_OR_: 0.33 per SD increase; 95% CI: 0.26 to 0.43) and increased risk of ASD (_MR_IVW_OR_: 1.51 per SD increase; 95% CI: 1.29 to 1.77), which was partly explained by cognitive ability (_MVMR_IVW_OR_ per SD increase: 1.24; 95%CI: 0.96 to 1.60).

**Conclusions:**

Genetic liability to ADHD and ASD is likely to affect educational attainment, independently of underlying cognitive ability.

Key Messages

- There is increasing evidence suggesting that children with attention deficit hyperactivity disorder (ADHD) are at risk of lower school performance and poor academic outcomes, and that adolescents with autism spectrum disorder (ASD) are less likely to transition to higher education compared with their typically developing peers.
- Additionally, cohort- and registry-based studies suggest an association between parental educational attainment and risk of ADHD and ASD in the offspring.
- The observational evidence seems to be in line with studies using whole-genome association findings. Specifically, genetic liability to ADHD shows negative genetic correlations and associations with educational attainment, whereas ASD presents positive associations.
- However, there is an absence of evidence from Mendelian randomization (MR) approaches on whether the associations are causal in nature, and on the possible contribution of cognitive ability.
- The present study uses a range of MR methods to assess whether there is a bidirectional causal link between genetic liability to ADHD, ASD and educational attainment and the possible role of cognitive ability.
- We found evidence of effects of genetic liability to ADHD on educational attainment, and evidence of effects of genetic liability to higher educational attainment on risk of ADHD which was independent of cognitive ability. Genetic liability to higher educational attainment was found to causally influence ASD in a positive direction although most of the effect was due to cognitive ability.
- The present study adds to the existing literature on ADHD, ASD and educational attainment by highlighting two main points: (i) the influence of genetic liability to ADHD and ASD on educational attainment, independently of cognitive ability; and (ii) the distinct contribution of genetic liability to higher educational attainment and cognitive ability on risk of ADHD and ASD.

## Introduction

Attention deficit hyperactivity disorder (ADHD) and autism spectrum disorder (ASD) are neurodevelopmental conditions that typically first manifest early in childhood and often persist into adulthood.^1^^,^^2^ Both conditions are associated with one of the strongest predictors of adult life outcomes and life satisfaction: educational attainment.^3^^,^^4^

Observational research evidence suggests that children with ADHD show lower academic performance compared with their typically developing peers,^5–7^ and the condition has been associated with increased risk of high school dropout.^8^ In the case of ASD, rates of transition to post-secondary education are much lower than in the general population, and only a small proportion of individuals with ASD who move on to higher education will graduate.^9^^,^^10^ Several factors have been found to predict educational attainment in children with ADHD or ASD, with one of the strongest being cognitive ability.^11^^,^^12^

The pattern of association of ADHD and ASD with educational outcomes is further complicated by parental educational attainment. Specifically, higher parental educational attainment has been found to be associated with increased risk of ASD in the offspring, whereas lower parental educational attainment with increased risk of ADHD.^13^^,^^14^

Observational evidence has been recently corroborated by studies using whole-genome approaches (linkage disequilibrium score regression,^15^ multi-trait analysis of GWAS- MTAG^16^) as well as aggregates of common risk variants [i.e. polygenic risk scores (PRS), suggesting strong negative genetic correlations and polygenic associations of educational attainment with ADHD and positive genetic correlations and polygenic associations with ASD].^17–19^

**Figure 1.**
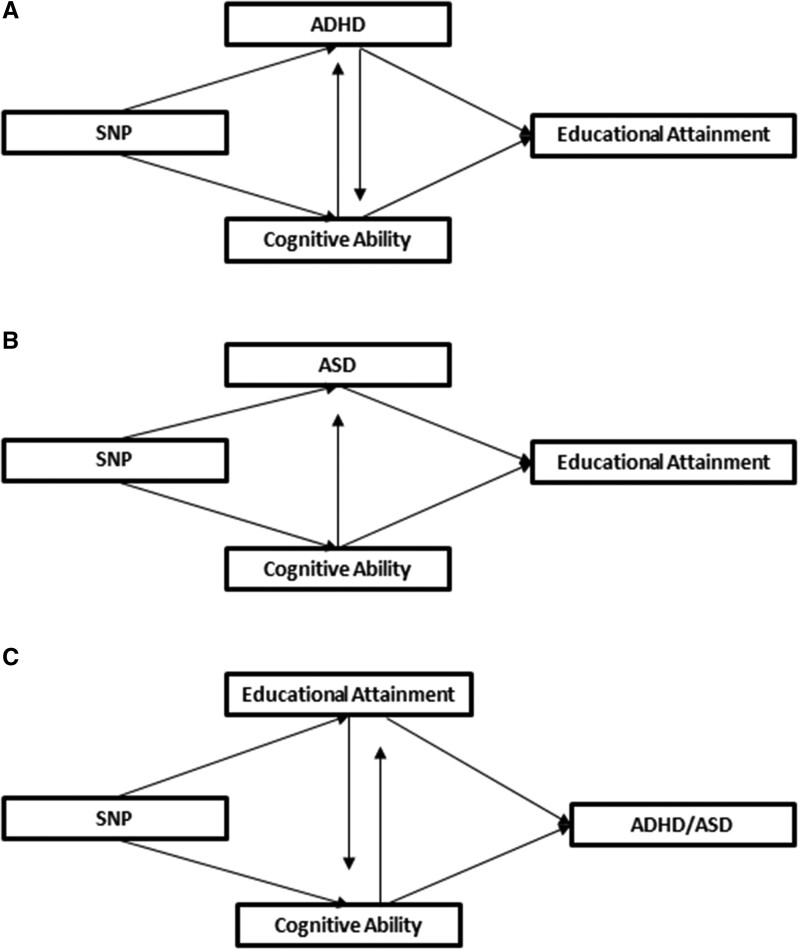
Possible causal pathways linking attention deficit hyperactivity disorder (ADHD), autism spectrum disorder (ASD), cognitive ability and educational attainment. Arrows between the exposures of interest were oriented based on previous Mendelian randomization (MR) evidence on the links of genetic liability to higher cognitive ability with ADHD, ASD and educational attainment.^24–26^ Specifically, pathway A has been based on evidence suggesting bidirectional causal links between genetic liability to ADHD and cognitive ability.^24^ Pathway B has been based on findings suggesting a causal effect of genetic liability to higher cognitive ability on ASD (but not vice versa^24^). Finally, pathway C has been based on evidence indicating bidirectional causal links between genetic liability to higher educational attainment and cognitive ability.^25^^,^^26^ All three pathways illustrate that the effects of the exposures of interest on the outcome are likely to be obscured by cognitive ability. Present figures are not formal or sufficient directed acyclic graphs (DAGs), and they do not cover all the causal pathways that might exist between the phenotypes

Despite increasing evidence suggesting polygenic and observational associations, little is known on whether there are causal links. Observational evidence can be hampered by measured or unmeasured confounding,^20^ and whole-genome approaches and PRS do not account for the potential influence of pleiotropic genetic variants.^21^ A useful method for overcoming these limitations is Mendelian randomization (MR).^22^ MR can be implemented as an instrumental variable analysis, using common genetic variants as proxies for environmental exposures and allowing the assessment of causal relations among the exposures with the outcome of interest.^23^ MR studies suggest bidirectional effects between genetic liability to ADHD and cognitive ability (Figure 1A) and effects of genetic liability to higher cognitive ability on ASD (Figure 1B).^24^ Education and cognitive ability causally influence each other according to MR findings (Figure 1C).^25^^,^^26^ Based on this, several possibilities linking ADHD, ASD and educational attainment could be proposed—some of them visualized in Figure 1.

We used genome-wide association study (GWAS) summary statistics and MR to investigate the causal links between genetic liability to ADHD, ASD and educational attainment (Figure 1). In MR settings, binary exposures (e.g. ADHD, ASD) are often approximated by continuous latent liabilities, assuming that they are normally distributed in the population.^27^^,^^28^ Under liability-threshold models of inheritance, an individual’s liability will be phenotypically expressed after the threshold has been exceeded, depending on the synergy of genetic variation, environmental factors and chance.^29–32^ This seems to be supported for ADHD and ASD, as high polygenic risk to the conditions has been associated with sub-threshold phenotypic expressions (traits) in the general population.^33^^,^^34^ We performed two-sample MR to assess whether genetic liability to ADHD and ASD (reflecting individual and potentially parental effects) are causally linked to educational attainment (Figure 1A and B), as well as whether genetic liability to higher educational attainment (reflecting potentially parental and dynastic effects) is causally linked to risk of ADHD and ASD (Figure 1C). We used an extension of MR, multivariable MR (MVMR), to assess whether any identified effects were independent of cognitive ability.

## Methods

The study was conducted using publicly available GWAS summary data. Ethics declarations for each dataset used in the present study can be found in the original publications.^17^^,^^18^^,^^24^^,^^36^

### Univariable two-sample Mendelian randomization

MR allows the estimation of causal links between an exposure and an outcome by utsing common genetic variants as instruments for the exposure of interest. The robustness of the method relies on assumptions that the genetic instruments should satisfy the following: (i) there must be a robust association between the implicated genetic variants and the exposure; (ii) the variants should not be associated with any confounders of the associations between the exposure and the outcome; and (iii) the variants should operate on the outcome entirely via the exposure.^35^ In this context, we applied two-sample MR in which the effects of the genetic instruments on the exposure and on the outcome are extracted from separate GWASs that have been conducted in independent samples from the same underlying population.^23^

### Genetic instruments

We used the latest publicly available GWAS summary statistics on ADHD,^17^ ASD,^18^ cognitive ability^24^ and educational attainment.^36^ Detailed information on the GWAS used can be found in the original publications.

In each GWAS dataset, we extracted all variants with a *P*-value <= 5 x 10^-8^. The identified variants were clumped using an r^2^ <0.01, within a 10 000 kb window, based on the 1000 Genomes European phase 3 reference panel. This resulted in 11 single nucleotide polymorphisms (SNPs) for ADHD, two SNPs for ASD, 481 SNPs for educational attainment and 212 SNPs for cognitive ability.

In order to increase the power of the ASD analyses, we relaxed the *P*-value threshold to 5 x 10^-7^. After clumping, we identified 10 independent (r^2^ <0.01) SNPs. A similar threshold (*P* <= 5 x 10^-6^) for instrument definition has been used in previous studies.^37^^,^^38^ However, we acknowledged the possibility that relaxing the inclusion threshold might lead to weak instrument bias in our estimates.^39^^,^^40^ In order to alleviate this, we performed robust adjusted profile score MR (MR raps), a method which provides an effect estimate robust to weak instrument bias.^40^ Details on the effect sizes, standard errors and *P*-values of the instruments can be found in Supplementary Table S1 (available as Supplementary data at *IJE* online).

For each analysis, instruments were extracted from the outcome GWASs. LD link online suite [LDlink: An Interactive Web Tool for Exploring Linkage Disequilibrium in Population Groups (nih.gov)]^41^ was used to identify linkage disequilibrium (LD) proxies when SNPs were not present in the outcome GWAS (r^2^ >0.9).

Finally, the alleles of the outcome variants were harmonized on the exposure so that the effect estimates of both exposure and outcome variants were expressed per effect allele increase. As the effect allele frequencies for the ADHD and ASD GWASs were not provided, when the harmonization of the exposure-outcome alleles was not possible, variants were excluded from the analyses as being palindromic. Detailed information on the harmonized datasets used in the present MR analyses can be found in Supplementary Table S2 (available as Supplementary data at *IJE* online). The full process followed and the final number of instruments used for each analysis are visualized in Supplementary Figure S1 (available as Supplementary data at *IJE* online). Two-sample MR analyses were performed using the TwoSampleMR R package.^42^

### Inverse variance weighted MR

The primary MR method used in this study was the inverse variance weighted (IVW) regression. It is a weighted generalized linear regression of the SNP-outcome coefficients on the SNP-exposure coefficients with a constrained to zero intercept term, giving an overall effect estimate of the exposure on the outcome.^43^

### Instrument strength

We assessed the strength of the instruments by calculating their F-statistic. As a rule of thumb, if the F >10, then the IVW is unlikely to suffer from weak instrument bias.^39^

### Sensitivity analyses

We tested for the presence of horizontal pleiotropy and assessed the robustness of the causal effect estimates using a series of sensitivity analyses, including: MR-Egger regression,^43^ weighted median,^44^ weighted mode,^45^ MR Raps^40^ and Steiger filtering.^46^ Detailed information on each sensitivity analysis conducted in the present study can be found in Supplementary Methods S1 (Supplementary data are available at *IJE* online).

### Multivariable Mendelian randomization

Where multiple exposures are suspected to have effects on an outcome, and the exposures are genetically and phenotypically correlated, univariable MR can yield biased effect estimates.^47^ Multivariable MR (MVMR) is an extension of MR, in which multiple exposures are entered within the same model, and their direct effects on the outcome can be estimated.^47^ We used MVMR to estimate the direct effects of ADHD, ASD and cognitive ability on educational attainment, and the direct effects of genetic liability to higher educational attainment and cognitive ability on risk of ADHD and ASD.

For each MVMR analysis, 212 genome-wide significant and independent (r^2^ <0.01, 10 000 kb-window) instruments for cognitive ability were added to the models. The full list of primary exposure instruments and cognitive ability instruments was clumped (r^2^ = 0.01), to ensure the absence of LD among the included SNPs, and then harmonized. The process followed and the number of instruments used in the MVMR analyses are visualized in Figure S2, available as Supplementary data at *IJE* online. We performed an inverse variance weighted (IVW) regression of the SNP-outcome coefficients on the SNP-exposure coefficients, entering the two exposures in the regression model simultaneously. Details on the effect sizes, standard errors and *P*-values of the cognitive ability instruments used can be found in Supplementary Table S1. We estimated the heterogeneity of the effect estimates of the instruments included using a modified version of the Q statistic as well as strength of the instruments of each exposure conditional on the other using a conditional F statistic.^48^ Evidence of heterogeneity indicates the possibility of biased effect estimates.^47^ Additionally, as a sensitivity analysis, in cases where Steiger filtering suggested that SNPs explained more variation in the outcome than in the exposure, we repeated MVMR analyses by removing these SNPs. Finally, we estimated robust to weak instruments direct effects. This approach is based on the minimization of Q statistics.^48^ We calculated confidence intervals using non-parametric bootstrap with 1000 iterations. Overlapping confidence intervals between the IVW MVMR, and the robust to weak instruments MVMR, provide support for the findings and strengthen their interpretation.

Details on the MVMR method and analytical process, as well as estimation of F and Q statistics, and robust to weak instruments MVMR, have been described elsewhere.^47^^,^^48^ MVMR analyses were performed using R, version 3.5. Q and conditional F statistics, as well as robust to weak instruments MVMR analyses, were conducted and estimated using the MVMR package [https://github.com/WSpiller/MVMR].

### Interpretation of the effect estimates

In the present study two of our exposures were binary; genetic liability to ASD and to ADHD. GWAS summary statistics for these exposures were estimated using logistic regression, and effect sizes represent log odds ratios.^17^^,^^18^ Therefore, the resulting MR estimates represent the change in the outcome per unit change in genetic liability to ADHD/ASD on the log odds scale. A unit increase in the log odds of the exposure corresponds to a 2.72-fold multiplicative increase in the odds of the exposure. For rare exposures, the odds are equal to the probability and, therefore, the MR estimate then represents the average change in the outcome per 2.72-fold increase in the prevalence of the exposure, in the case of the present study genetic liability to ADHD/ASD.^49^ It may aid interpretation of the estimates to think about the change in the outcome per doubling (2-fold increase) the prevalence of the exposure (i.e. genetic liability to ADHD/ASD).^49^ Thus, as recommended by Burgess and Labrecque, 2018,^49^ in the analyses investigating the effects of genetic liability to ADHD and ASD on educational attainment, we firstly multiplied the estimates by the standard deviation (SD) of educational attainment (years of education SD = 4.2)^36^ to convert them to months/days (where appropriate) of education, and then multiplied by ln^2^ to express the effect of a doubling of genetic liability to ADHD, ASD. In the analyses investigating the effects of genetic liability to higher educational attainment on risk of ADHD and ASD, MR estimates and 95% confidence intervals (CI) are expressed per one-SD increase in educational attainment on the odds of developing ADHD and ASD.

## Results

### Total effect of genetic liability to ADHD on educational attainment

For the univariable MR, the F statistic of the ADHD instruments ranged from 30 to 51. The IVW effect estimate suggested that a doubling in the genetic liability to ADHD decreases years of education by around 3 months (_MR_IVW: −3.6 months per doubling of genetic liability to ADHD; 95% CI: -5.2 to -2.1; *P*val = 5 x 10^−6^) (Table 1).

**Table 1.** The total and direct (not mediated via cognitive ability) effect estimates of genetic liability to attention deficit hyperactivity disorder (ADHD) and autism spectrum disorder (ASD) on educational attainment

Exposure: genetic liability to ADHD (log-odds). Outcome: educational attainment (SD)				
Type of effect	Beta	SE	95% CI	*P*-value
Total effect	−0.103	0.023	−0.15, −0.06	5 x 10^−6^
Direct effect	−0.049	0.014	−0.08, −0.02	0.0004
Exposure: genetic liability to ASD (log-odds). Outcome: educational attainment (SD)				
Type of effect	Beta	SE	95% CI	*P*-value
Total effect	0.004	0.031	−0.06, 0.07	0.9
Direct effect	0.028	0.013	0.002, 0.05	0.03

The effect estimates were directionally consistent across the sensitivity analyses performed. There was limited evidence of horizontal pleiotropy in the analyses, as suggested by the MR-Egger intercept (intercept = -0.008; *P*val = 0.41). Steiger filtering suggested that the direction of the effect was correct for all the ADHD instruments. Supplementary Table S3 (Supplementary data are available at *IJE* online) shows the effect estimates, standard errors and *P*-values derived from the primary and sensitivity analyses.

### Direct effect of genetic liability to ADHD on educational attainment, independent of cognitive ability

For the MVMR, the direct effect of genetic liability to ADHD on educational attainment (i.e. not via cognitive ability) was approximately 50% smaller than the total effect (_MVMR_IVW: -1.7 months per doubling of genetic liability to ADHD; 95% CI: -2.8 to -0.7; *P*val = 4 x 10^-4^) (Table 1). There was evidence of heterogeneity among the effect estimates of the instruments as indicated by the Q statistic (Q = 693; *P*val = 1 x 10^-61^). Supplementary Table S4 (available as Supplementary data at *IJE* online) contains the direct effect estimates of genetic liability to ADHD and cognitive ability on educational attainment, and the robust to weak instruments direct effect estimates and corresponding confidence intervals, as well as the conditional F statistics of the instruments.

### Total effect of genetic liability to ASD on educational attainment

For the univariable MR, the F statistic of the ASD instruments ranged from 26 to 36. There was little evidence of an effect of genetic liability to ASD on educational attainment (_MR_IVW: 3 days, per doubling of genetic liability to ASD; 95% CI: -2.1 months to 2.4 months; *P*val = 0.9) (Table 1). The confidence intervals across primary and sensitivity analyses were largely overlapping. There was little evidence of directional horizontal pleiotropy (MR-Egger intercept = -0.009; *P*val = 0.42) (Table 2). Steiger filtering suggested that the effect direction was correct for all the ASD SNPs. Supplementary Table S5 (available as Supplementary data at *IJE* online) contains detailed information on the effect estimates, standard errors and *P*-values across primary and sensitivity analyses.

**Table 2.** The total and direct (not mediated through cognitive ability) effect estimates of genetic liability to higher educational attainment on risk of attention deficit hyperactivity disorder (ADHD) and autism spectrum disorder (ASD) diagnosis

Exposure: genetic liability to higher educational attainment (SD). Outcome: ADHD				
Type of effect	Odds ratio	SE	95% CI	*P*-value
Total effect	0.30	0.079	0.26, 0.36	6x10^−51^
Direct effect	0.33	0.126	0.26, 0.43	6x10^−17^
Exposure: genetic liability to higher educational attainment (SD). Outcome: ASD				
Type of effect	Odds ratio	SE	95% CI	*P*-value
Total effect	1.51	0.082	1.29, 1.77	5 x 10^−7^
Direct effect	1.24	0.13	0.96, 1.60	0.09

### Direct effect of genetic liability to ASD on educational attainment, independent of cognitive ability

When including cognitive ability in the MVMR models, there was some evidence suggesting that a doubling in the genetic liability to ASD had a small positive direct effect on educational attainment, approximately 29 days (_MVMR_IVW: 1 month per doubling of genetic liability to ASD; 95% CI: 3 days to 1.7 months; *P*val = 0.03) (Table 1). There was strong evidence of heterogeneity among the effect estimates of the instruments as suggested by the Q statistic (Q = 2380; *P*val <1 x 10^-10^). The direct effect estimates of genetic liability to ASD and cognitive ability on educational attainment and the robust to weak instruments direct effect estimates and corresponding confidence intervals, , the conditional F statistics of the instruments, can be found in Supplementary Table S6 (available as Supplementary data at *IJE* online).

### Total effect of genetic liability to higher educational attainment on risk of ADHD

In the univariable MR, the F statistic of the educational attainment instruments ranged from 30 to 240. There was evidence suggesting that one-SD increase in genetic liability to higher educational attainment (i.e. ≈ 4.2 years of schooling) was associated with approximately 70% lower risk of ADHD (IVW_OR_: 0.30; 95% CI: 0.26 to 0.36; *P*val = 6 x 10^-51^) (Table 2). There was limited evidence of unbalanced horizontal pleiotropy (MR-Egger intercept: −0.003; *P*val = 0.47). Both MR-Egger and SIMEX-adjusted MR-Egger estimates, accounting for these pleiotropic effects, were directionally in agreement with the IVW, and the confidence intervals across the methods were largely overlapping (Supplementary Table S7a, available as Supplementary data at *IJE* online). Steiger filtering identified 81 instruments explaining more variation in ADHD than in educational attainment. Removing those attenuated the identified effect estimate, which was still suggestive of a strong effect of genetic liability to higher educational attainment on risk of ADHD (Supplementary Table S7b).

### Direct effect of genetic liability to higher educational attainment on risk of ADHD, independent of cognitive ability

In the MVMR, the estimated effect of genetic liability to higher educational attainment on risk of ADHD, independent of cognitive ability, was largely comparable to the total effect (IVW_OR_: 0.33; 95% CI: 0.26 to 0.43; *P*val = 6 x 10^-17^) (Table 2). There was evidence of heterogeneity among the effect estimates of the instruments as indicated by the Q statistic (Q = 843; *P*val = 2 x 10^-22^). A direct effect was identified even after removing the instruments identified through Steiger filtering (Supplementary Table S8, available as Supplementary data at *IJE* online). Supplementary Table S8 contains the direct effect estimates of genetic liability to higher EA and cognitive ability on risk of ADHD, and the robust to weak instruments direct effect estimates and corresponding confidence intervals, , the conditional F-statistics of the instruments.

### Total effect of genetic liability to higher educational attainment on risk of ASD

In the univariable MR, the F statistic of the educational attainment instruments ranged from 30 to 240. There was evidence suggesting that genetic liability to higher educational attainment was associated with increased risk of ASD (IVW_OR_: 1.51 per SD increase; 95% CI: 1.29 to 1.77; *P*val = 4 x 10^-7^) (Table 2). The estimated effect was directionally consistent across the sensitivity analyses (Supplementary Table S9a, available as Supplementary data at *IJE* online) and there was limited evidence to indicate the presence of unbalanced horizontal pleiotropy (MR-Egger intercept: -0.007; *P*val = 0.11). Steiger filtering suggested that 62 SNPs associated with educational attainment explained more variation in ASD and these were removed. The exclusion of these SNPs, despite attenuating the primary analysis effect estimate, was suggestive of an effect of genetic liability to higher educational attainment on ASD (Supplementary Table S9b).

### Direct effect of genetic liability to higher educational attainment on risk of ASD, independent of cognitive ability

**Figure 2:**
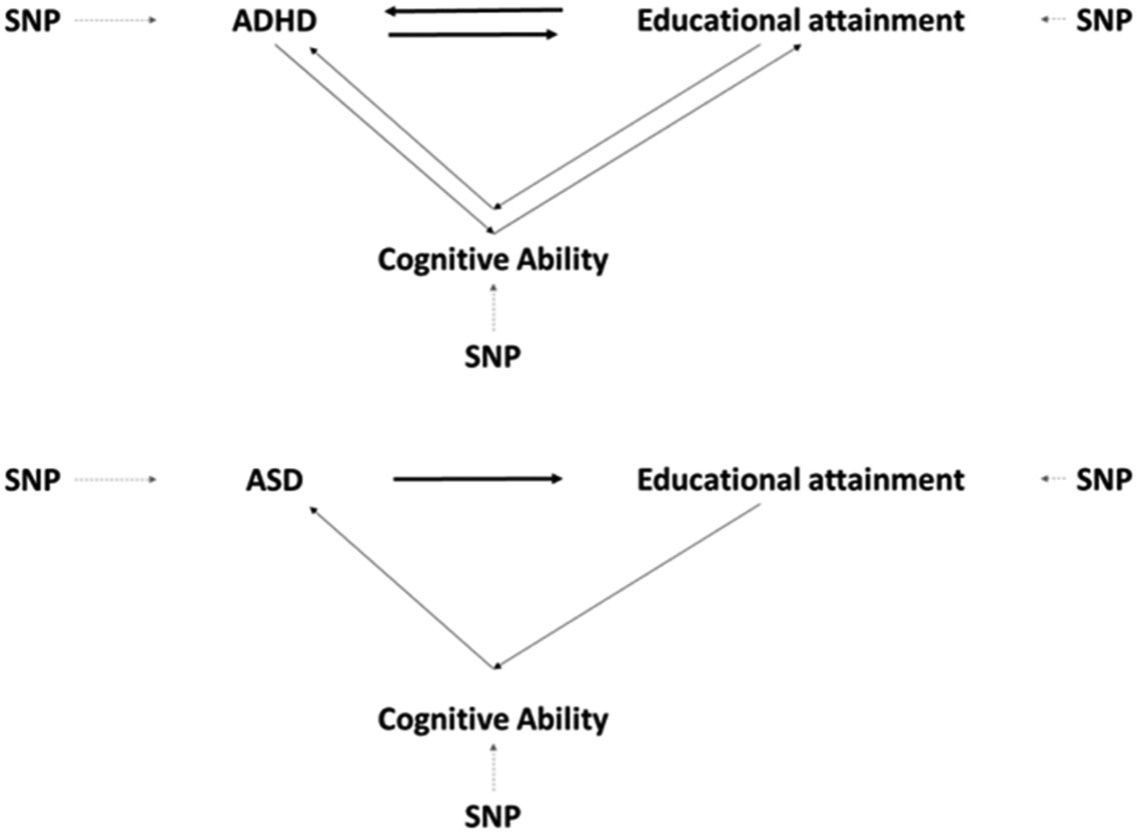
Relationships between genetic liability to attention deficit hyperactivity disorder (ADHD), autism spectrum disorder (ASD) and educational attainment, suggested by the results of the Mendelian randomization (MR) and multivariable Mendelian randomization (MVMR) analyses in the present study. Bold arrows indicate the direct, independent of cognitive ability, effects identified, whereas light arrows indicate the total effects

In the MVMR, the direct effect of genetic liability to higher educational attainment on risk of ASD, not mediated through cognitive ability, was smaller than the total effect (IVW_OR_: 1.24 per SD increase; 95% CI: 0.96 to 1.6; *P*val = 0.09) (Table 2). There was evidence of heterogeneity among the effect estimates of each instrument included as indicated by the Q statistic (Q = 910; *P*val = 2 x 10^-27^). After removing SNPs identified through Steiger filtering which explain more variation in the outcome, the effect estimate attenuated further, providing limited evidence of a direct effect of genetic liability to higher educational attainment on risk of ASD (Supplementary Table S10, available as Supplementary data at *IJE* online). Supplementary Table S10 contains the direct effect estimates of genetic liability to higher educational attainment and cognitive ability on risk of ASD, as estimated by MVMR analysis, and the robust to weak instruments direct effect estimates and corresponding confidence intervals, as well as the conditional F statistic of the instruments. The relationships suggested by the results of the present MR and MVMR analyses are visualized in Figure 2.

## Discussion

This is the first study to investigate the bidirectional associations between genetic liability to ADHD, ASD and educational attainment and explore the possible role of cognitive ability in the identified effects using two-sample MR. Despite the genetic and phenotypic overlap of ADHD and ASD, we found distinct associations of ADHD and ASD genetic liability with educational attainment.

### Bidirectional associations between genetic liability to ADHD and educational attainment

We found evidence consistent with a negative effect of genetic liability to ADHD on educational attainment, which was only partly attributed to the effects of cognitive ability. This implies that it is ADHD genetic liability itself, not just cognitive ability, which causally influences lower educational attainment. This is in line with a large body of observational evidence suggesting that beyond cognitive ability, ADHD traits and disorder are associated with poor academic outcomes.^50^^,^^51^ In addition, there is increasing evidence using large cohort and registry data suggesting beneficial effects of ADHD medication on academic performance and outcomes.^52^^,^^53^ Therefore, our results encourage future research into early interventions to ameliorate ADHD phenotypic expressions, in order to improve the academic outcomes of children with ADHD.

We also found that genetic liability to higher educational attainment, over and above cognitive ability, was associated with lower risk of ADHD. This could be explained in the context of educational attainment being associated with several socioeconomic position and lifestyle indicators.^54^^,^^55^ Therefore, our finding supports existing observational evidence suggesting that parental socioeconomic position is associated with risk of ADHD in the offspring.^13^^,^^56^ This association could be mediated by optimal lifestyle and general health factors during pregnancy, which are known also to be associated with ADHD,^57^^,^^58^ as well as better prenatal care and access to health care services.

Another possible explanation could be dynastic effects (i.e. the phenotypic expression of the parental genotype affects the phenotype of the offspring).^59^ This implies that parents with higher educational attainment might place more emphasis on their child’s academic performance, have more access to educational resources and learning stimuli and cultivate more learning behaviours. In fact, parental resource capital (including income, education and educational material at home), and parental self-efficacy beliefs to help their child, have been found to be important predictors of offspring academic performance.^60^^,^^61^ Parental emphasis on broader learning behaviours might lead to milder expression, masking or compensation of the ADHD symptomatology in their children, resulting therefore in ADHD being missed from diagnosis. Academic performance and educational attainment reflect a range of abilities beyond cognitive ability, such as social behaviour,^62^ behavioural discipline^63^ and imitation,^64^ thus it could be hypothesized that children with genetic liability to higher educational attainment might mask ADHD symptomatology.

### Bidirectional associations between genetic liability to ASD and educational attainment

In the case of ASD, we found little evidence suggesting a positive effect of genetic liability to ASD on educational attainment. The effect was identified only after the direct, independent of cognitive ability, effects were estimated. In order for this finding to be interpreted, the observational associations of ASD with educational attainment and cognitive ability need to be considered. Observational evidence suggests that academic performance in ASD is highly variable and dependent on several factors including cognitive ability, learning disabilities and executive functioning, as well as family socioeconomic indicators.^65–69^ In this context, the present finding suggests that over and beyond cognitive factors, phenotypic characteristics of ASD might have small but beneficial effects on educational attainment. Such phenotypic characteristics could include hyper-systemizing and attention to detail.^70^

We also identified a positive total effect of genetic liability to higher educational attainment on risk of ASD, which MVMR analyses revealed was attributed, at least partially, to the effects of cognitive ability. This is in line with a recent study using the polygenic transmission disequilibrium test (pTDT) in families of children with ASD, suggesting that parental polygenic risk for higher educational attainment is associated with autism risk in the proband, and these probands tend to inherit more alleles associated with higher cognitive ability compared with their siblings without ASD.^71^

Before reaching conclusions, it is worth considering the extent to which the identified bidirectional relationships, between genetic liability to ASD and educational attainment, reflect selection bias. Evidence from the USA, as well as the UK, seem to suggest an association between parental socioeconomic position indicators and autism diagnosis in the offspring, possibly due to better access to health care.^72–76^ This could possibly indicate selection bias due to socioeconomic position/factors in the ASD GWAS sample. Although recent evidence from Swedish registry data suggests that the associations between ASD, educational attainment and cognitive ability are unlikely to be influenced by selection bias,^14^ future research including samples across countries and socioeconomic strata is necessary.

Overall, in both ADHD and ASD findings, alternative explanations including diagnostic masking and selection bias cannot be rejected. Little is currently known on the sociodemographic, socioeconomic and educational factors that might influence ADHD and ASD diagnosis. Specifically, availability and access to health care services, family income and educational background, even perceived societal stigma, might be defining factors of which children will end up having a diagnosis and therefore being included in current GWASs.

### Strengths and limitations

Our study benefited from using the latest and largest publicly available GWAS data on all the phenotypes of interest. We performed thorough sensitivity analyses to assess the effect of pleiotropic variants used as instruments for each phenotype. We were also able to model the effects of each exposure along with cognitive ability, so that direct and indirect effects were quantified.

One of the limitations of the study is the use of instruments for ASD below the genome-wide significance threshold (*P*val <5 x 10^-7^). This might have made the ASD analyses prone to weak instrument bias, biasing the estimated effect towards the null. However, F statistic and MR Raps analyses do not support this interpretation. Second, there was sample overlap between the educational attainment and cognitive ability GWASs, as both studies included participants from UK Biobank (overlapping participants *n* = 195 653). This overlap represented approximately 27% of the educational attainment GWAS participants. Overlap between the exposure and outcome GWASs (as in the case of MVMR analyses of ADHD/ASD and cognitive ability on educational attainment) can lead to bias towards the observational estimate.^77^ Therefore, for a potentially more robust estimate of the effect of cognitive ability on educational attainment, we orient the readers elsewhere.^25^^,^^26^ Third, high levels of heterogeneity were identified in our analyses, and this could potentially suggest the influence of pleiotropic variants in our effect estimates. However, causal effect estimates were largely consistent, and confidence intervals overlapped across several sensitivity analyses conducted in the present study. In the case of MVMR, results should be interpreted with caution, as there was evidence of potentially weak instruments and increased heterogeneity. However, direct effect estimates were consistent when we applied robust to weak instruments MVMR methods.

It is worth considering that ADHD and ASD are highly heterogeneous phenotypes, and different phenotypic dimensions have been found to have distinct genetic underpinnings.^78^^,^^79^ The GWASs used in the present study included individuals within the broad range of ADHD and ASD diagnoses, and it is therefore not possible to decipher whether the effects identified in the present study are driven by different phenotypic sub-clusters within ADHD and ASD.

### Future directions

The present findings highlight the importance of further research into the underlying genetic components and phenotypic characteristics that might be driving the links between genetic liability to ADHD, ASD, cognitive ability and educational attainment.

Specifically, educational attainment variants are highly pleiotropic, presenting strong genetic overlaps with mental health as well as socioeconomic traits.^80^^,^^81^ In the context of the present analyses it was not possible to disentangle whether the identified effects were driven by genetic variants specific to educational attainment. Approaches such as genomic structural equation modelling (genomic SEM), allowing the identification of sets of genetic variants that explain variation unique to educational attainment and variation that is shared with other traits,^82^ are expected to offer valuable insights into the identified causal links between educational attainment, ADHD and ASD.

In addition, novel MR approaches are expected to offer valuable insights into whether the identified causal links are a result of dynastic effects or assortative mating. In the case of the present study, these are possibilities that could not be excluded. However, investigating these possibilities would be possible through within-families MR, a novel approach leveraging genetic information on sibling pairs and family trios to assess the influence of dynastic effects and assortative mating in the causal effect estimates.^59^^,^^83^

Furthermore, the availability of large birth cohorts across countries (e.g. ALSPAC,^84^ MoBa^85^) offers the opportunity to investigate which specific phenotypic expressions of genetic liability to ADHD and ASD are associated with educational attainment. There is increasing observational evidence suggesting associations between specific ADHD and ASD traits with academic performance. For instance, inattention (rather than hyperactivity) has been found to be an important predictor of academic outcomes,^51^^,^^86^ whereas less is known in the case of ASD-related traits.

## Conclusions

Despite the genetic and phenotypic overlap of two neurodevelopmental conditions, ADHD and ASD, we found distinct effects of ADHD and ASD genetic liability on educational attainment. Further research in necessary in order to elucidate whether the identified causal patterns reflect parentally transmitted effects, diagnostic masking or selection bias, and to dissect the broad phenotypes of ADHD, ASD and educational attainment by focusing on investigating causal relationships within the several sub-dimensions.

## Supplementary Data

Supplementary data are available at *IJE* online.

## Funding

The Medical Research Council (MRC) and the University of Bristol support the MRC Integrative Epidemiology Unit [MC_UU_00011/1, MC_UU_00011/3, MC_UU_00011/5]. The Economics and Social Research Council (ESRC) support N.M.D. via a Future Research Leaders grant [ES/N000757/1] and the Norwegian Research Council support N.M.D. via grant number 295989. This research was funded in whole, or in part, by the Wellcome Trust. C.D. is funded by the Wellcome Trust [108902/B/15/Z]. B.L. and L.R. are supported by the Wellcome Trust (grant ref: 204895/Z/16/Z) awarded to A.T., G.D.S., E.S. and K.T. For the purpose of Open Access, the author has applied a CC BY public copyright licence to any Author Accepted Manuscript version arising from this submission. L.D.H. is supported by a Career Development Award from the UK Medical Research Council [MR/M020894/1] and project entitled ‘social and economic value of health’, which is part of the Health Foundation’s Efficiency Research Programme (grant id: 807293). The Health Foundation is an independent charity committed to bringing about better health and health care for people in the UK. No funding body has influenced data collection, analysis or interpretation. This publication is the work of the authors, who serve as the guarantors for the contents of this paper.

## Supplementary Material

dyab107_Supplementary_Data

## Data Availability

ADHD and ASD GWAS summary data were accessed from [https://www.med.unc.edu/pgc/download-results/]. Educational attainment data were accessed from [https://www.thessgac.org/data]. Cognitive ability data were accessed from [https://ctg.cncr.nl/software/summary_statistics].
