# Correction to: Is genetic liability to ADHD and ASD causally linked to educational attainment?

**DOI:** 10.1093/ije/dyad157

**Published:** 2023-11-10

This is a correction to: Christina Dardani, Lucy Riglin, Beate Leppert, Eleanor Sanderson, Dheeraj Rai, Laura D Howe, George Davey Smith, Kate Tilling, Anita Thapar, Neil M Davies, Emma Anderson, Evie Stergiakouli, Is genetic liability to ADHD and ASD causally linked to educational attainment?, *International Journal of Epidemiology*, Volume 50, Issue 6, December 2021, Pages 2011–2023, https://doi.org/10.1093/ije/dyab107

Upon original publication, this manuscript’s received date was incorrectly given as 17 August 2021, instead of 17 August 2020.

The Publisher apologizes for this error.

These details have been corrected only in this correction notice to preserve the published version of record.

