# Supplementary material for: Is genetic liability to ADHD and ASD causally linked to educational attainment?
Source: Int J Epidemiol. 2021 Jun 7;50(6):2011–23. doi: 10.1093/ije/dyab107 (PMC8743131; doi:10.1093/ije/dyab107)
Supplement: dyab107_Supplementary_Data [file dyab107_supplementary_data.docx]

**CONTENTS**

| eMethods S1 | Sensitivity Analyses to test for horizontal pleiotropy and the robustness of the causal effect estimates. | Page: 2 |
| --- | --- | --- |
| Figure S1 | Flowcharts visualising the process for instrument definition, extraction and harmonisation for the two-sample MR analyses conducted in the present study. | Page: 3 |
| Figure S2 | Flowchart visualising the process for instrument definition for the MVMR analyses conducted in the present study. | Page: 4 |
| Table S1 | Instruments for ADHD, ASD, educational attainment (EA) and cognitive ability used in the MR and MVMR analyses. | Pages: 5- 21 |
| Table S2 | Harmonised instruments used in the MR analyses investigating:  2a. the causal effect of genetic liability to ADHD on educational attainment; 2b.the causal effect of genetic liability to ASD on educational attainment;  2c. the causal effect of genetic liability to higher educational attainment on risk of ADHD;  2d. the causal effect of genetic liability to higher educational attainment on risk of ASD | Pages: 22- 58 |
| Table S3 | Causal effect estimates of ADHD genetic liability on educational attainment derived from two-sample MR analyses. | Page: 59 |
| Table S4 | Direct causal effect estimates of genetic liability to ADHD and cognitive ability on educational attainment, as estimated by MVMR analysis. | Page: 59 |
| Table S5 | Causal effect estimates of ASD genetic liability on educational attainment derived from two-sample MR analyses. | Page: 60 |
| Table S6 | Direct causal effect estimates of genetic liability to ASD and cognitive ability on educational attainment, as estimated by MVMR analysis. | Page: 60 |
| Table S7a | Causal effect estimates of genetic liability to higher educational attainment on risk of ADHD derived from two-sample MR analyses. | Page: 61 |
| Table S7b | Causal effect estimates of genetic liability to higher educational attainment on risk of ADHD after removing instruments identified to explain more variation in the outcome through Steiger filtering. | Page: 61 |
| Table S8 | Direct causal effect estimates of genetic liability to higher EA and cognitive ability on risk of ADHD, as estimated by MVMR analysis. | Page: 61 |
| Table S9a | Causal effect estimates of genetic liability to higher educational attainment on risk of ASD derived from two-sample MR analyses. | Page: 62 |
| Table S9b | Causal effect estimates of genetic liability to higher educational attainment on risk of ASD after removing instruments identified to explain more variation in the outcome through Steiger filtering. | Page: 62 |
| Table S10 | Direct causal effect estimates of genetic liability to higher EA and cognitive ability on risk of ASD, as estimated by MVMR analysis. | Page: 62 |
|  | References | Page: 63 |

**eMethods S1.** Sensitivity Analyses to test for horizontal pleiotropy and the robustness of the causal effect estimates.

To test for the presence of horizontal pleiotropy (i.e. whereby a genetic variant has independent effects on multiple phenotypes) we used MR-Egger regression^1,2^. MR-Egger (in contrast to IVW) allows the intercept term to be unconstrained. The intercept parameter indicates the overall unbalanced horizontal pleiotropic effect of the SNPs on the outcome (i.e. a direct effect of each SNP on the outcome, independent of the exposure, which would violate MR assumptions), while the slope offers an effect estimate accounting for directional pleiotropy. MR-Egger, as all MR analyses, assumes the gene-exposure association estimates are measured without error (i.e. the no measurement error [NOME] assumption)^1^. We assessed the NOME assumption using an adaptation of the I^2^ statistic^3^ within the two-sample MR context, which is referred to as I^2^_GX_^4^_._ I^2^_GX_ provides an estimate of the degree of regression dilution in the MR-Egger estimate, due to uncertainty in the SNP-exposure estimates. We then used simulation extrapolation (SIMEX) to adjust the MR-Egger estimate for this dilution, as described previously^4^. We conducted weighted median MR which provides an estimate of effect even when only 50% of the genetic variants included in the analysis are valid instruments for the exposure^5^. We performed weighted mode MR which provides an effect estimate based on the assumption that the most common effect of the genetic variants stems from valid instruments^6^.

In the case of the analyses investigating the effects of genetic liability to ASD on educational attainment we additionally performed the robust adjusted profile score MR approach (MR Raps), which provides an effect estimate robust to weak instrument bias and systematic pleiotropy^7^. The approach was chosen by acknowledging the possibility that the relaxed p-value threshold used for instrument inclusion (p-value<=5x10^-7^) might have made the analysis prone to weak instrument bias. Finally, we used Steiger filtering in order to exclude the possibility that the direction of the identified effects was influenced by the large sample size and power of the educational attainment GWAS or the strong genetic correlations between ADHD, ASD and educational attainment^8^. The method assesses whether SNPs used as proxies for the exposure are explaining more variation in the outcome, and therefore an MR assumption is violated: instruments influence the outcome via pathways other than the exposure. The method allows the detection and removal of these variants from subsequent MR analyses.


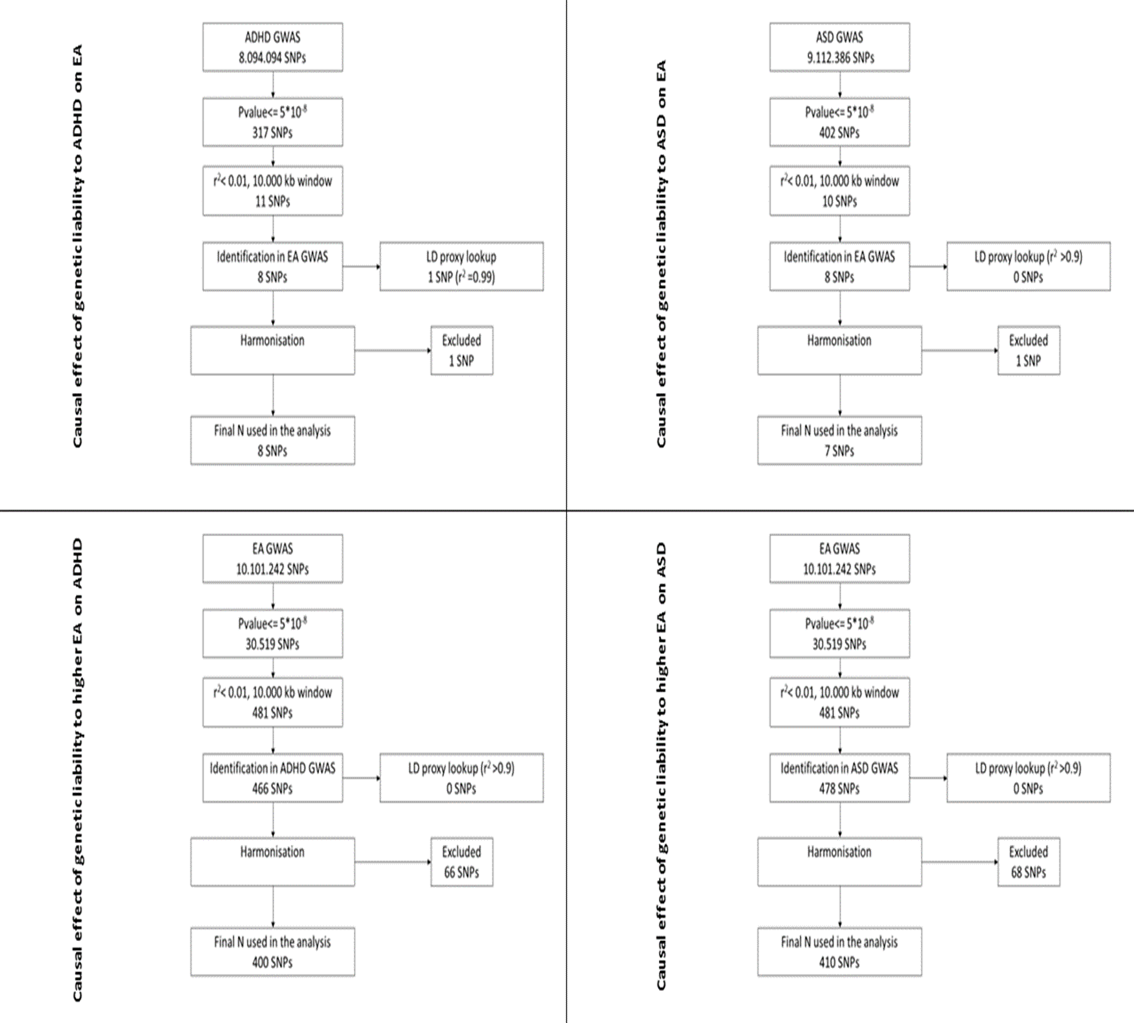


**Figure S1:** Flowcharts visualising the process for instrument definition, extraction and harmonisation for the two-sample MR analyses conducted in the present study.


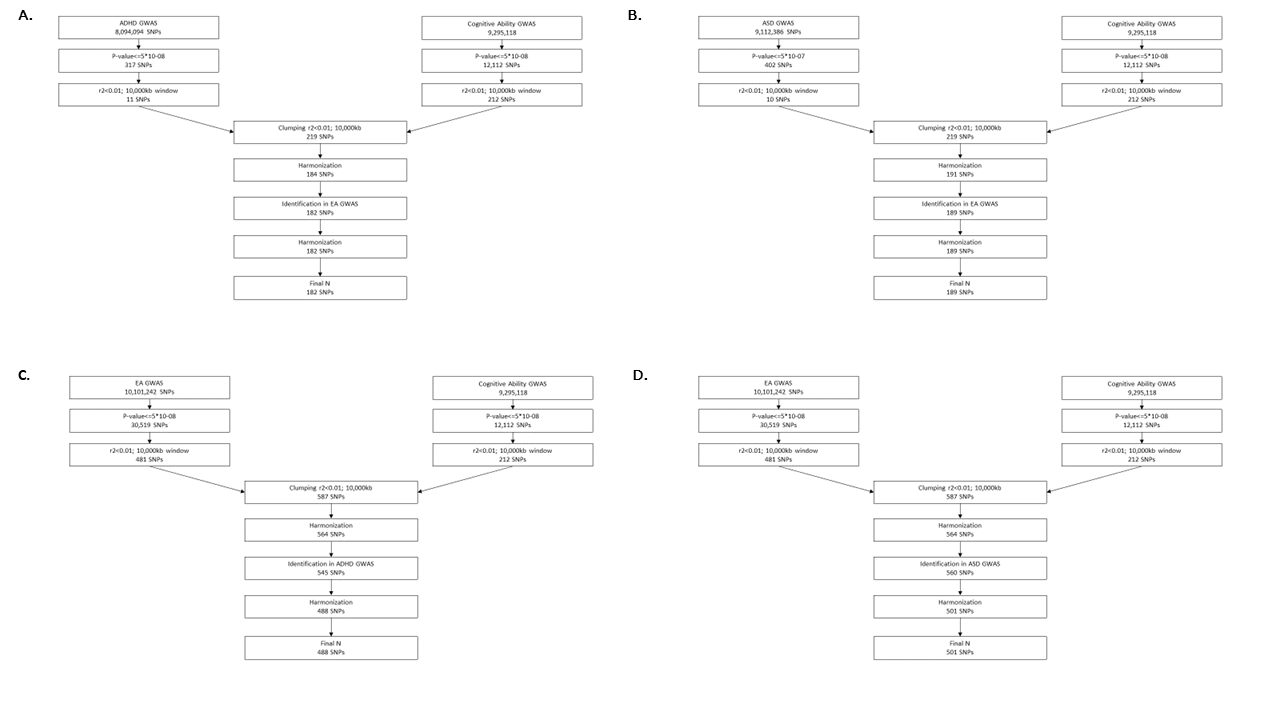


**Figure S2**. Flowcharts visualising the process for instrument definition for the MVMR analyses conducted in the present study.

**Table S1**. Instruments for ADHD, ASD, educational attainment (EA) and cognitive ability used in the MR and MVMR analyses.

| **ADHD instruments** (r2=0.01; kb:10,000; p<=5e-08) | | | | | |
| --- | --- | --- | --- | --- | --- |
| SNP | A1 | A2 | logOR | SE | P |
| rs11420276 | G | GT | 0.107104 | 0.0149 | 6.45E-13 |
| rs1222063 | A | G | 0.096201 | 0.0174 | 3.07E-08 |
| rs4858241 | T | G | 0.082197 | 0.0143 | 8.17E-09 |
| rs28411770 | T | C | 0.086104 | 0.0151 | 1.15E-08 |
| rs4916723 | A | C | -0.0778 | 0.0138 | 1.81E-08 |
| rs10262192 | A | G | 0.074096 | 0.0135 | 3.66E-08 |
| rs74760947 | A | G | -0.17961 | 0.0317 | 1.39E-08 |
| rs11591402 | A | T | -0.0924 | 0.0164 | 1.76E-08 |
| rs1427829 | A | G | 0.082197 | 0.0136 | 1.35E-09 |
| rs8039398 | T | C | -0.08 | 0.0135 | 2.99E-09 |
| rs212178 | A | G | -0.1171 | 0.0205 | 1.20E-08 |

| **ASD instruments** (r2=0.01; kb:10,000; p<=5e-07) | | | | | |
| --- | --- | --- | --- | --- | --- |
| SNP | A1 | A2 | logOR | SE | P |
| rs910805 | A | G | -0.0957 | 0.016 | 2.04E-09 |
| rs111931861 | A | G | -0.2169 | 0.0409 | 1.12E-07 |
| rs11481126 | GA | G | 0.073799 | 0.014 | 1.26E-07 |
| rs325485 | A | G | 0.072804 | 0.0143 | 3.25E-07 |
| rs112635299 | T | G | 0.220997 | 0.0432 | 3.04E-07 |
| rs10099100 | C | G | 0.084304 | 0.0147 | 1.07E-08 |
| rs45595836 | T | C | 0.138996 | 0.0272 | 3.13E-07 |
| rs2391769 | A | G | -0.0769 | 0.0145 | 1.14E-07 |
| rs6701243 | A | C | 0.073501 | 0.0144 | 3.07E-07 |
| rs1452075 | T | C | 0.080704 | 0.0155 | 2.07E-07 |

| **EA instruments** (r2=0.01; kb:10,000; p<=5e-08) | | | | | |
| --- | --- | --- | --- | --- | --- |
| SNP | A1 | A2 | Beta | SE | P |
| rs13090388 | C | T | -0.02852 | 0.00184 | 4.29E-54 |
| rs7029718 | G | A | -0.02439 | 0.00174 | 1.85E-44 |
| rs9372625 | A | G | 0.02383 | 0.00176 | 6.76E-42 |
| rs1334297 | A | G | 0.02449 | 0.00192 | 3.06E-37 |
| rs4700393 | G | A | 0.02086 | 0.0017 | 1.51E-34 |
| rs11123818 | G | A | -0.02081 | 0.00175 | 1.72E-32 |
| rs34316 | C | A | -0.02016 | 0.00177 | 3.35E-30 |
| rs10773002 | T | A | -0.02191 | 0.00197 | 8.68E-29 |
| rs9964724 | C | T | -0.01978 | 0.00183 | 2.66E-27 |
| rs3747631 | G | C | -0.02207 | 0.00208 | 2.97E-26 |
| rs1620977 | G | A | -0.02046 | 0.00195 | 1.14E-25 |
| rs2819336 | T | C | 0.01828 | 0.00177 | 5.46E-25 |
| rs11678980 | G | A | 0.01744 | 0.00172 | 4.29E-24 |
| rs10189857 | G | A | -0.01725 | 0.00171 | 6.70E-24 |
| rs1008078 | T | C | -0.01738 | 0.00173 | 1.20E-23 |
| rs73344830 | G | A | -0.0172 | 0.00172 | 1.95E-23 |
| rs4787457 | G | A | -0.01741 | 0.00176 | 3.73E-23 |
| rs1689510 | G | C | -0.01761 | 0.0018 | 1.40E-22 |
| rs1566085 | T | G | 0.01645 | 0.00171 | 6.90E-22 |
| rs10963297 | G | C | 0.01904 | 0.00198 | 7.36E-22 |
| rs1455350 | T | A | 0.01614 | 0.0017 | 2.61E-21 |
| rs56048629 | T | C | -0.01654 | 0.00176 | 6.76E-21 |
| rs10191758 | A | G | -0.01631 | 0.00175 | 9.60E-21 |
| rs1391438 | T | C | 0.0167 | 0.00183 | 5.79E-20 |
| rs79265434 | A | G | -0.02331 | 0.00262 | 6.08E-19 |
| rs4382592 | T | G | -0.01636 | 0.00185 | 1.01E-18 |
| rs7924036 | T | G | 0.01501 | 0.0017 | 1.07E-18 |
| rs176218 | G | T | -0.01883 | 0.00215 | 1.85E-18 |
| rs9616906 | G | A | -0.01497 | 0.00172 | 2.92E-18 |
| rs6557171 | C | T | 0.01567 | 0.00181 | 4.15E-18 |
| rs66568921 | G | T | 0.01565 | 0.00182 | 7.49E-18 |
| rs1892417 | C | T | -0.01732 | 0.00202 | 1.12E-17 |
| rs74998289 | T | G | 0.01821 | 0.00213 | 1.31E-17 |
| rs35417702 | C | T | 0.01445 | 0.0017 | 1.93E-17 |
| rs1618725 | T | C | 0.01477 | 0.00174 | 2.22E-17 |
| rs11601122 | G | A | -0.01947 | 0.0023 | 2.24E-17 |
| rs10765775 | A | G | 0.01488 | 0.00176 | 2.62E-17 |
| rs12375949 | T | C | -0.01447 | 0.00172 | 3.31E-17 |
| rs62444881 | C | T | -0.01815 | 0.00217 | 5.79E-17 |
| rs62166492 | A | G | 0.02846 | 0.0034 | 6.03E-17 |
| rs9349956 | A | C | -0.01881 | 0.00225 | 6.28E-17 |
| rs3897821 | A | G | 0.01502 | 0.0018 | 8.25E-17 |
| rs2179152 | C | T | 0.01455 | 0.00176 | 1.21E-16 |
| rs12643771 | C | T | -0.01518 | 0.00184 | 1.61E-16 |
| rs55736314 | C | G | -0.01431 | 0.00174 | 1.63E-16 |
| rs2725370 | T | C | -0.01536 | 0.00187 | 1.97E-16 |
| rs12468040 | G | T | -0.01432 | 0.00175 | 2.46E-16 |
| rs72828517 | C | T | 0.01836 | 0.00224 | 2.83E-16 |
| rs406413 | A | T | 0.01695 | 0.00209 | 4.84E-16 |
| rs10875121 | G | C | -0.01834 | 0.00226 | 5.53E-16 |
| rs2245901 | A | G | -0.01403 | 0.00174 | 6.26E-16 |
| rs1054442 | C | A | 0.01426 | 0.00177 | 8.42E-16 |
| rs1964927 | G | A | -0.01423 | 0.00177 | 9.90E-16 |
| rs8020034 | G | A | -0.01782 | 0.00223 | 1.17E-15 |
| rs2971970 | T | G | -0.01654 | 0.00207 | 1.25E-15 |
| rs62184480 | T | C | -0.01528 | 0.00191 | 1.28E-15 |
| rs56391344 | A | G | 0.01571 | 0.00197 | 1.34E-15 |
| rs6493265 | C | T | 0.01385 | 0.00174 | 1.70E-15 |
| rs7788620 | G | A | -0.01635 | 0.00206 | 1.84E-15 |
| rs363096 | C | T | 0.01363 | 0.00172 | 2.04E-15 |
| rs13141210 | C | T | -0.01361 | 0.00172 | 2.26E-15 |
| rs2545798 | T | A | 0.01346 | 0.00171 | 3.11E-15 |
| rs10810099 | A | G | -0.015 | 0.0019 | 3.31E-15 |
| rs1043209 | A | G | 0.01364 | 0.00174 | 4.22E-15 |
| rs55771711 | C | G | 0.01555 | 0.00199 | 5.41E-15 |
| rs2347526 | T | C | -0.01395 | 0.00179 | 6.84E-15 |
| rs635754 | A | G | 0.0134 | 0.00173 | 8.01E-15 |
| rs13010288 | G | T | -0.01953 | 0.00252 | 1.04E-14 |
| rs3026996 | A | C | 0.01537 | 0.00199 | 1.05E-14 |
| rs35309068 | G | T | 0.01321 | 0.00171 | 1.15E-14 |
| rs1569092 | A | G | 0.01807 | 0.00234 | 1.16E-14 |
| rs613872 | G | T | 0.0175 | 0.00227 | 1.20E-14 |
| rs17489649 | A | G | 0.0139 | 0.00181 | 1.57E-14 |
| rs4757957 | C | G | 0.0141 | 0.00184 | 1.81E-14 |
| rs11082975 | G | C | 0.01309 | 0.00172 | 2.52E-14 |
| rs115000530 | T | A | 0.02892 | 0.00381 | 3.30E-14 |
| rs10215082 | A | G | -0.01303 | 0.00172 | 3.33E-14 |
| rs76076331 | C | T | -0.01873 | 0.00248 | 4.40E-14 |
| rs72829857 | G | A | 0.01516 | 0.00202 | 5.39E-14 |
| rs998887 | A | C | 0.01272 | 0.0017 | 7.42E-14 |
| rs59484001 | C | T | 0.02926 | 0.00391 | 7.42E-14 |
| rs10496091 | G | A | 0.01403 | 0.00188 | 7.47E-14 |
| rs2998315 | G | A | 0.01269 | 0.00171 | 1.12E-13 |
| rs11657342 | A | G | 0.01404 | 0.00191 | 1.94E-13 |
| rs790647 | C | A | 0.01482 | 0.00202 | 2.17E-13 |
| rs36083520 | C | T | 0.01629 | 0.00223 | 2.60E-13 |
| rs4810227 | A | G | 0.01272 | 0.00175 | 3.57E-13 |
| rs35475880 | G | T | 0.01511 | 0.00208 | 3.80E-13 |
| rs66671632 | C | T | 0.01838 | 0.00254 | 4.59E-13 |
| rs4073894 | A | G | 0.01524 | 0.00211 | 5.40E-13 |
| rs59123361 | A | G | -0.02094 | 0.00291 | 5.87E-13 |
| rs4726070 | G | A | -0.01251 | 0.00174 | 5.95E-13 |
| rs728054 | G | A | 0.01274 | 0.00177 | 6.65E-13 |
| rs17425572 | G | A | -0.01224 | 0.0017 | 6.89E-13 |
| rs79269403 | G | A | -0.01447 | 0.00204 | 1.17E-12 |
| rs2067854 | A | G | 0.01477 | 0.00209 | 1.38E-12 |
| rs17568389 | A | T | 0.01201 | 0.0017 | 1.53E-12 |
| rs3812281 | C | T | -0.01228 | 0.00174 | 1.58E-12 |
| rs13422673 | C | T | 0.01201 | 0.0017 | 1.74E-12 |
| rs17598675 | C | T | 0.01199 | 0.0017 | 1.75E-12 |
| rs11081529 | T | C | 0.01311 | 0.00186 | 1.82E-12 |
| rs11871429 | A | G | 0.01425 | 0.00202 | 1.92E-12 |
| rs1475974 | C | T | 0.01269 | 0.0018 | 2.02E-12 |
| rs1584469 | T | C | -0.01303 | 0.00185 | 2.10E-12 |
| rs2297600 | G | T | -0.01569 | 0.00223 | 2.16E-12 |
| rs35532491 | T | A | 0.02007 | 0.00286 | 2.42E-12 |
| rs11774212 | T | C | 0.01196 | 0.00171 | 2.74E-12 |
| rs17563464 | A | C | -0.01477 | 0.00212 | 2.89E-12 |
| rs71415374 | T | C | 0.02142 | 0.00307 | 2.93E-12 |
| rs6805241 | C | T | -0.01413 | 0.00203 | 3.09E-12 |
| rs969512 | T | A | 0.01249 | 0.00179 | 3.22E-12 |
| rs6731373 | G | A | 0.01256 | 0.00181 | 3.47E-12 |
| rs16822665 | T | C | 0.01286 | 0.00185 | 3.57E-12 |
| rs10098073 | A | C | -0.01185 | 0.00171 | 3.71E-12 |
| rs11635092 | A | G | -0.01231 | 0.00177 | 3.89E-12 |
| rs6867851 | C | G | -0.012 | 0.00173 | 3.97E-12 |
| rs7920624 | T | A | -0.01181 | 0.0017 | 3.97E-12 |
| rs6666119 | A | G | -0.01269 | 0.00183 | 4.16E-12 |
| rs2964197 | T | C | 0.01177 | 0.0017 | 4.70E-12 |
| rs1051474 | T | C | -0.01301 | 0.00188 | 4.86E-12 |
| rs9536961 | A | G | -0.01242 | 0.0018 | 4.96E-12 |
| rs4352658 | T | C | -0.0212 | 0.00308 | 5.55E-12 |
| rs339054 | G | T | 0.0117 | 0.0017 | 5.90E-12 |
| rs7526112 | T | G | 0.01215 | 0.00177 | 6.10E-12 |
| rs9936270 | T | C | -0.0136 | 0.00198 | 6.43E-12 |
| rs9882532 | C | T | -0.01208 | 0.00177 | 8.17E-12 |
| rs7737905 | T | G | -0.01285 | 0.00188 | 8.34E-12 |
| rs76878669 | C | G | 0.01399 | 0.00205 | 8.67E-12 |
| rs4877516 | T | A | 0.01163 | 0.00171 | 1.02E-11 |
| rs175325 | A | T | -0.01179 | 0.00174 | 1.11E-11 |
| rs10984445 | G | A | -0.01158 | 0.00171 | 1.17E-11 |
| rs35039375 | G | A | -0.01983 | 0.00293 | 1.22E-11 |
| rs301800 | T | C | 0.01516 | 0.00224 | 1.33E-11 |
| rs12170452 | G | A | -0.01153 | 0.00171 | 1.40E-11 |
| rs10862376 | T | A | -0.01616 | 0.00239 | 1.40E-11 |
| rs2287838 | A | G | -0.01152 | 0.00171 | 1.53E-11 |
| rs6774533 | T | C | 0.0126 | 0.00187 | 1.73E-11 |
| rs401687 | G | C | -0.01144 | 0.0017 | 1.86E-11 |
| rs1106090 | A | G | 0.01173 | 0.00175 | 2.09E-11 |
| rs7977614 | G | A | 0.01325 | 0.00198 | 2.09E-11 |
| rs12602286 | T | G | 0.01701 | 0.00255 | 2.37E-11 |
| rs16995054 | C | T | 0.0139 | 0.00208 | 2.52E-11 |
| rs17565975 | A | G | -0.01142 | 0.00171 | 2.56E-11 |
| rs7575637 | A | G | 0.01136 | 0.00171 | 2.86E-11 |
| rs11646221 | T | G | 0.01144 | 0.00172 | 3.01E-11 |
| rs9771228 | T | C | 0.01182 | 0.00178 | 3.07E-11 |
| rs10266047 | G | C | 0.01132 | 0.00171 | 3.27E-11 |
| rs17048855 | A | G | 0.01184 | 0.00179 | 3.27E-11 |
| rs62247449 | C | G | 0.01137 | 0.00171 | 3.31E-11 |
| rs10456918 | A | C | -0.01485 | 0.00224 | 3.67E-11 |
| rs9938678 | A | T | -0.01355 | 0.00205 | 4.12E-11 |
| rs1143770 | C | T | -0.01136 | 0.00172 | 4.31E-11 |
| rs72693550 | C | A | 0.01542 | 0.00234 | 4.57E-11 |
| rs9933256 | A | G | 0.01134 | 0.00172 | 4.57E-11 |
| rs17570033 | G | T | 0.01862 | 0.00283 | 4.65E-11 |
| rs11694904 | T | C | 0.01215 | 0.00185 | 4.78E-11 |
| rs11210934 | G | A | -0.01275 | 0.00194 | 4.84E-11 |
| rs28513670 | G | A | 0.01477 | 0.00225 | 5.06E-11 |
| rs8008382 | T | C | -0.01208 | 0.00185 | 6.12E-11 |
| rs2406253 | G | A | -0.01411 | 0.00216 | 6.40E-11 |
| rs56194430 | T | C | -0.01514 | 0.00232 | 6.65E-11 |
| rs12515541 | G | T | 0.01135 | 0.00174 | 6.95E-11 |
| rs7233920 | A | G | -0.01315 | 0.00202 | 7.13E-11 |
| rs9436866 | C | A | 0.01882 | 0.00289 | 7.45E-11 |
| rs6123924 | A | G | 0.01528 | 0.00235 | 7.55E-11 |
| rs66641143 | T | C | -0.03162 | 0.00486 | 8.04E-11 |
| rs17551064 | G | A | -0.01493 | 0.0023 | 8.62E-11 |
| rs11732657 | A | G | -0.01274 | 0.00197 | 9.54E-11 |
| rs10060023 | C | T | -0.01178 | 0.00182 | 9.59E-11 |
| rs281302 | A | G | -0.0111 | 0.00172 | 9.84E-11 |
| rs2923431 | C | G | 0.0114 | 0.00176 | 9.84E-11 |
| rs2302761 | T | C | 0.01354 | 0.00209 | 1.00E-10 |
| rs74944275 | C | T | -0.02739 | 0.00424 | 1.02E-10 |
| rs12926704 | A | G | -0.01737 | 0.00269 | 1.03E-10 |
| rs9289300 | T | C | -0.01512 | 0.00234 | 1.10E-10 |
| rs1550816 | C | T | -0.01107 | 0.00172 | 1.33E-10 |
| rs2052285 | A | G | 0.01123 | 0.00175 | 1.34E-10 |
| rs192436652 | C | T | 0.03497 | 0.00545 | 1.35E-10 |
| rs9342482 | T | G | 0.01264 | 0.00197 | 1.36E-10 |
| rs11019128 | T | C | -0.01123 | 0.00175 | 1.43E-10 |
| rs837080 | C | T | 0.01092 | 0.0017 | 1.43E-10 |
| rs12764593 | C | G | 0.02293 | 0.00358 | 1.53E-10 |
| rs5763431 | T | C | 0.01125 | 0.00176 | 1.53E-10 |
| rs4652135 | C | A | -0.01219 | 0.0019 | 1.54E-10 |
| rs2256965 | G | A | -0.01128 | 0.00176 | 1.59E-10 |
| rs62177359 | A | C | -0.0304 | 0.00475 | 1.60E-10 |
| rs6573552 | T | C | -0.01086 | 0.0017 | 1.62E-10 |
| rs11663602 | A | C | -0.01213 | 0.0019 | 1.64E-10 |
| rs140711597 | C | G | 0.04026 | 0.0063 | 1.66E-10 |
| rs2447535 | A | G | -0.01181 | 0.00185 | 1.69E-10 |
| rs4972400 | G | A | -0.01156 | 0.00181 | 1.70E-10 |
| rs1291818 | C | T | -0.01085 | 0.0017 | 1.78E-10 |
| rs79523955 | A | G | 0.01802 | 0.00283 | 1.87E-10 |
| rs6513959 | A | G | 0.01177 | 0.00185 | 1.88E-10 |
| rs67890737 | C | A | 0.01141 | 0.00179 | 2.01E-10 |
| rs736282 | C | T | -0.01082 | 0.0017 | 2.07E-10 |
| rs57016874 | C | T | -0.02902 | 0.00457 | 2.09E-10 |
| rs337637 | G | A | -0.01123 | 0.00177 | 2.11E-10 |
| rs268120 | A | G | 0.01244 | 0.00196 | 2.13E-10 |
| rs9529119 | C | G | 0.01295 | 0.00204 | 2.13E-10 |
| rs10887801 | G | T | -0.01087 | 0.00171 | 2.27E-10 |
| rs111852224 | T | C | 0.01663 | 0.00262 | 2.34E-10 |
| rs6429082 | C | T | 0.01078 | 0.0017 | 2.37E-10 |
| rs9556958 | C | T | 0.0108 | 0.0017 | 2.38E-10 |
| rs7803932 | A | G | 0.0143 | 0.00226 | 2.44E-10 |
| rs4384309 | G | A | -0.0109 | 0.00172 | 2.52E-10 |
| rs11023749 | A | G | 0.01132 | 0.0018 | 2.96E-10 |
| rs1558727 | C | T | 0.01069 | 0.0017 | 3.09E-10 |
| rs76608582 | C | A | -0.02798 | 0.00445 | 3.11E-10 |
| rs9503598 | A | G | 0.01079 | 0.00171 | 3.12E-10 |
| rs12332731 | A | T | 0.01374 | 0.00218 | 3.12E-10 |
| rs17060737 | C | T | -0.01192 | 0.0019 | 3.18E-10 |
| rs143163770 | T | C | -0.01625 | 0.00258 | 3.22E-10 |
| rs31940 | G | A | -0.01548 | 0.00246 | 3.24E-10 |
| rs10994777 | G | A | -0.0146 | 0.00232 | 3.36E-10 |
| rs1245829 | A | T | -0.01083 | 0.00173 | 3.51E-10 |
| rs7796203 | G | A | 0.01074 | 0.00171 | 3.60E-10 |
| rs13145650 | C | T | 0.01918 | 0.00306 | 3.80E-10 |
| rs60483752 | G | C | -0.01078 | 0.00172 | 3.89E-10 |
| rs77702819 | G | T | -0.01863 | 0.00298 | 3.92E-10 |
| rs4839155 | T | G | 0.01251 | 0.002 | 3.94E-10 |
| rs2496482 | C | T | -0.01109 | 0.00177 | 4.04E-10 |
| rs13035874 | A | G | -0.01114 | 0.00178 | 4.34E-10 |
| rs1827540 | G | A | -0.0106 | 0.0017 | 4.50E-10 |
| rs13010566 | A | C | -0.0106 | 0.0017 | 4.59E-10 |
| rs12290350 | T | C | 0.013 | 0.00209 | 4.70E-10 |
| rs36119825 | G | A | -0.01063 | 0.00171 | 4.82E-10 |
| rs2478208 | C | G | -0.0106 | 0.0017 | 4.82E-10 |
| rs10460095 | G | A | 0.01066 | 0.00171 | 4.87E-10 |
| rs61757207 | G | A | -0.04941 | 0.00795 | 5.09E-10 |
| rs1952183 | G | A | 0.01055 | 0.0017 | 5.57E-10 |
| rs34485537 | T | C | 0.01075 | 0.00173 | 5.67E-10 |
| rs73301698 | A | G | -0.01291 | 0.00208 | 5.81E-10 |
| rs6821231 | C | T | 0.01217 | 0.00197 | 6.06E-10 |
| rs1979969 | G | T | 0.01208 | 0.00195 | 6.17E-10 |
| rs892612 | C | A | 0.01464 | 0.00237 | 6.63E-10 |
| rs10940921 | G | T | -0.01089 | 0.00177 | 7.00E-10 |
| rs13147223 | A | G | 0.01087 | 0.00176 | 7.09E-10 |
| rs13029509 | A | G | -0.01049 | 0.0017 | 7.17E-10 |
| rs10205801 | A | G | -0.01053 | 0.00171 | 7.17E-10 |
| rs7108020 | C | A | -0.01094 | 0.00178 | 7.35E-10 |
| rs10752262 | C | T | -0.01072 | 0.00174 | 7.80E-10 |
| rs2220926 | T | C | -0.01055 | 0.00172 | 7.99E-10 |
| rs2034670 | G | A | -0.01312 | 0.00214 | 8.09E-10 |
| rs17126938 | T | C | -0.01536 | 0.0025 | 8.14E-10 |
| rs112806496 | G | C | 0.0187 | 0.00305 | 8.28E-10 |
| rs1061801 | A | G | -0.01354 | 0.0022 | 8.34E-10 |
| rs12574281 | A | C | -0.01077 | 0.00176 | 8.85E-10 |
| rs9914918 | G | A | -0.01155 | 0.00189 | 8.90E-10 |
| rs17428076 | C | G | 0.01216 | 0.00198 | 8.90E-10 |
| rs12694681 | G | T | -0.01123 | 0.00183 | 9.06E-10 |
| rs7603132 | G | A | -0.01317 | 0.00215 | 9.17E-10 |
| rs2820314 | A | C | 0.011 | 0.0018 | 9.34E-10 |
| rs4328757 | T | C | 0.01067 | 0.00174 | 9.39E-10 |
| rs12712269 | C | T | 0.01063 | 0.00174 | 9.51E-10 |
| rs3800546 | G | C | -0.01183 | 0.00194 | 9.73E-10 |
| rs11703948 | A | G | -0.01737 | 0.00285 | 1.06E-09 |
| rs12646216 | T | C | 0.01062 | 0.00174 | 1.10E-09 |
| rs145590108 | G | T | -0.02232 | 0.00367 | 1.14E-09 |
| rs4976445 | T | C | 0.012 | 0.00197 | 1.16E-09 |
| rs28373063 | G | C | -0.01389 | 0.00229 | 1.23E-09 |
| rs7257460 | T | C | 0.01145 | 0.00189 | 1.25E-09 |
| rs7332724 | T | C | -0.01149 | 0.00189 | 1.26E-09 |
| rs4860734 | A | G | -0.0114 | 0.00188 | 1.29E-09 |
| rs77025239 | A | G | -0.01422 | 0.00234 | 1.33E-09 |
| rs7188873 | A | G | 0.0106 | 0.00175 | 1.34E-09 |
| rs74545339 | A | G | -0.01626 | 0.00269 | 1.44E-09 |
| rs67885444 | T | C | 0.01406 | 0.00232 | 1.48E-09 |
| rs6122735 | T | C | 0.0105 | 0.00174 | 1.49E-09 |
| rs4442732 | G | A | -0.01063 | 0.00176 | 1.49E-09 |
| rs10772644 | G | C | -0.01614 | 0.00267 | 1.50E-09 |
| rs35316276 | C | T | -0.01173 | 0.00194 | 1.52E-09 |
| rs72677177 | G | A | -0.0105 | 0.00174 | 1.55E-09 |
| rs7321274 | G | A | -0.01275 | 0.00211 | 1.59E-09 |
| rs2706762 | T | C | 0.01484 | 0.00246 | 1.59E-09 |
| rs9704097 | A | C | -0.0103 | 0.00171 | 1.61E-09 |
| rs1105307 | A | G | -0.01173 | 0.00195 | 1.67E-09 |
| rs6959891 | G | A | -0.01136 | 0.00189 | 1.74E-09 |
| rs3768480 | G | C | -0.01038 | 0.00172 | 1.74E-09 |
| rs2885198 | A | G | 0.01025 | 0.0017 | 1.81E-09 |
| rs80171383 | A | G | 0.0145 | 0.00241 | 1.83E-09 |
| rs1671770 | C | A | -0.01342 | 0.00223 | 1.91E-09 |
| rs62157915 | T | C | -0.02091 | 0.00348 | 1.96E-09 |
| rs73496688 | T | A | -0.01429 | 0.00238 | 2.04E-09 |
| rs242093 | A | G | -0.01031 | 0.00172 | 2.07E-09 |
| rs11754551 | C | T | 0.01627 | 0.00272 | 2.08E-09 |
| rs8052297 | T | G | -0.01029 | 0.00172 | 2.10E-09 |
| rs17638867 | T | C | 0.01329 | 0.00222 | 2.11E-09 |
| rs2365376 | C | A | -0.0107 | 0.00179 | 2.15E-09 |
| rs12503522 | C | T | 0.01125 | 0.00188 | 2.24E-09 |
| rs4846724 | G | A | -0.01018 | 0.0017 | 2.26E-09 |
| rs78721320 | G | A | -0.01307 | 0.00219 | 2.28E-09 |
| rs4778058 | C | T | 0.01017 | 0.0017 | 2.40E-09 |
| rs6457996 | C | T | -0.01014 | 0.0017 | 2.43E-09 |
| rs79375112 | G | A | -0.01529 | 0.00257 | 2.63E-09 |
| rs6436555 | C | A | -0.01012 | 0.0017 | 2.70E-09 |
| rs10120798 | A | G | -0.01028 | 0.00173 | 2.74E-09 |
| rs3890802 | A | G | -0.01133 | 0.00191 | 2.74E-09 |
| rs12646297 | G | A | 0.0111 | 0.00187 | 2.76E-09 |
| rs4984541 | A | G | -0.01233 | 0.00207 | 2.77E-09 |
| rs11655029 | T | C | -0.0109 | 0.00183 | 2.84E-09 |
| rs118093058 | T | G | 0.01525 | 0.00257 | 2.87E-09 |
| rs117799466 | G | C | -0.01173 | 0.00198 | 2.91E-09 |
| rs710629 | A | G | 0.01053 | 0.00177 | 2.96E-09 |
| rs730384 | G | A | -0.01016 | 0.00171 | 3.01E-09 |
| rs1569723 | A | C | 0.01168 | 0.00197 | 3.01E-09 |
| rs1925587 | C | T | -0.01016 | 0.00171 | 3.03E-09 |
| rs1527878 | G | A | 0.01182 | 0.00199 | 3.08E-09 |
| rs71646142 | C | T | -0.01286 | 0.00217 | 3.11E-09 |
| rs4984682 | G | C | 0.01202 | 0.00203 | 3.13E-09 |
| rs1880692 | A | G | 0.01008 | 0.0017 | 3.17E-09 |
| rs1427298 | C | T | -0.0102 | 0.00172 | 3.28E-09 |
| rs4964046 | G | A | 0.01053 | 0.00178 | 3.36E-09 |
| rs3796348 | G | A | -0.01032 | 0.00175 | 3.66E-09 |
| rs1866823 | A | G | 0.01009 | 0.00171 | 3.81E-09 |
| rs112969166 | G | C | -0.01008 | 0.00171 | 3.81E-09 |
| rs10856785 | T | C | -0.01132 | 0.00192 | 3.83E-09 |
| rs1599381 | A | G | 0.01002 | 0.0017 | 3.83E-09 |
| rs10519504 | T | G | 0.01381 | 0.00235 | 3.92E-09 |
| rs10795831 | T | G | -0.01223 | 0.00208 | 3.99E-09 |
| rs7597126 | C | T | 0.01009 | 0.00172 | 4.20E-09 |
| rs1931259 | A | G | -0.01237 | 0.00211 | 4.30E-09 |
| rs2414072 | A | T | -0.01005 | 0.00171 | 4.35E-09 |
| rs1405876 | G | T | -0.01038 | 0.00177 | 4.40E-09 |
| rs11708375 | G | C | 0.01376 | 0.00235 | 4.58E-09 |
| rs13130765 | C | G | -0.01014 | 0.00173 | 4.69E-09 |
| rs13163062 | T | C | 0.01007 | 0.00172 | 4.71E-09 |
| rs17110109 | C | T | 0.01023 | 0.00175 | 4.71E-09 |
| rs2833483 | C | T | 0.01974 | 0.00337 | 4.77E-09 |
| rs11620355 | G | A | -0.01756 | 0.003 | 4.77E-09 |
| rs111821073 | C | T | -0.01385 | 0.00237 | 4.85E-09 |
| rs7012546 | C | T | -0.01009 | 0.00172 | 4.93E-09 |
| rs7595950 | C | T | -0.00993 | 0.0017 | 4.99E-09 |
| rs7833201 | C | G | -0.01532 | 0.00262 | 5.05E-09 |
| rs10417097 | G | C | 0.01014 | 0.00174 | 5.17E-09 |
| rs939400 | G | T | 0.01031 | 0.00177 | 5.28E-09 |
| rs7139165 | C | A | -0.01136 | 0.00195 | 5.29E-09 |
| rs35919256 | C | A | -0.01032 | 0.00177 | 5.56E-09 |
| rs9371881 | G | A | -0.01036 | 0.00178 | 5.76E-09 |
| rs4369924 | A | G | 0.01362 | 0.00234 | 5.82E-09 |
| rs1592757 | C | G | -0.01045 | 0.0018 | 5.89E-09 |
| rs12682775 | T | C | -0.01187 | 0.00204 | 5.99E-09 |
| rs17321729 | A | G | 0.01141 | 0.00196 | 6.06E-09 |
| rs6736898 | A | G | 0.01032 | 0.00178 | 6.09E-09 |
| rs9320493 | G | A | -0.01394 | 0.0024 | 6.13E-09 |
| rs34394051 | G | A | 0.01392 | 0.0024 | 6.20E-09 |
| rs6067645 | A | G | -0.01003 | 0.00173 | 6.38E-09 |
| rs4728278 | C | G | 0.01066 | 0.00184 | 6.38E-09 |
| rs12151248 | C | T | 0.01573 | 0.00271 | 6.75E-09 |
| rs4675248 | G | A | 0.01004 | 0.00173 | 6.75E-09 |
| rs62142891 | G | A | -0.01121 | 0.00194 | 6.94E-09 |
| rs7215889 | T | C | 0.01132 | 0.00196 | 7.06E-09 |
| rs9886703 | A | T | -0.01315 | 0.00227 | 7.14E-09 |
| rs663234 | G | C | -0.01005 | 0.00174 | 7.39E-09 |
| rs12118513 | T | A | 0.01215 | 0.0021 | 7.47E-09 |
| rs72840994 | G | T | 0.01247 | 0.00216 | 7.77E-09 |
| rs60096640 | G | A | -0.01574 | 0.00273 | 7.82E-09 |
| rs10906186 | C | T | -0.00991 | 0.00172 | 7.91E-09 |
| rs59480703 | G | C | 0.01237 | 0.00215 | 8.32E-09 |
| rs12724430 | T | G | 0.00979 | 0.0017 | 8.32E-09 |
| rs11259919 | A | G | -0.01093 | 0.0019 | 9.31E-09 |
| rs7136760 | A | G | 0.01018 | 0.00177 | 9.36E-09 |
| rs77128898 | T | C | -0.02769 | 0.00482 | 9.47E-09 |
| rs152603 | G | A | 0.01019 | 0.00177 | 9.47E-09 |
| rs11223560 | G | A | -0.00995 | 0.00173 | 9.68E-09 |
| rs35518360 | A | T | 0.01875 | 0.00327 | 9.79E-09 |
| rs139244147 | G | A | 0.02095 | 0.00365 | 9.90E-09 |
| rs12908232 | A | G | 0.00975 | 0.0017 | 1.00E-08 |
| rs4641552 | C | A | -0.01926 | 0.00336 | 1.02E-08 |
| rs10797055 | G | A | 0.00986 | 0.00172 | 1.04E-08 |
| rs56330207 | A | G | 0.01184 | 0.00207 | 1.05E-08 |
| rs71432775 | A | G | 0.0113 | 0.00198 | 1.06E-08 |
| rs115438240 | G | T | 0.02162 | 0.00378 | 1.08E-08 |
| rs3809634 | A | G | -0.01058 | 0.00185 | 1.09E-08 |
| rs139612798 | C | T | 0.02918 | 0.00511 | 1.11E-08 |
| rs4780563 | G | A | 0.01398 | 0.00245 | 1.12E-08 |
| rs702606 | T | C | 0.01427 | 0.0025 | 1.12E-08 |
| rs6678474 | T | A | -0.02927 | 0.00513 | 1.13E-08 |
| rs12655753 | A | G | -0.02903 | 0.00509 | 1.18E-08 |
| rs7117878 | A | C | -0.01036 | 0.00182 | 1.18E-08 |
| rs743316 | T | C | 0.01185 | 0.00208 | 1.20E-08 |
| rs72486027 | C | T | 0.01123 | 0.00197 | 1.25E-08 |
| rs10879676 | T | C | -0.00982 | 0.00173 | 1.26E-08 |
| rs7625428 | C | T | -0.00989 | 0.00174 | 1.26E-08 |
| rs9386319 | G | A | 0.00991 | 0.00174 | 1.27E-08 |
| rs2343094 | A | G | 0.01029 | 0.00181 | 1.29E-08 |
| rs113182709 | G | A | -0.03225 | 0.00567 | 1.29E-08 |
| rs4760687 | G | A | 0.01039 | 0.00183 | 1.30E-08 |
| rs3781339 | T | C | -0.01247 | 0.00219 | 1.30E-08 |
| rs997123 | C | T | 0.00966 | 0.0017 | 1.32E-08 |
| rs61996546 | C | T | -0.00965 | 0.0017 | 1.34E-08 |
| rs11609711 | C | G | 0.01371 | 0.00241 | 1.35E-08 |
| rs4731413 | G | A | -0.01211 | 0.00213 | 1.37E-08 |
| rs62190914 | T | C | 0.01001 | 0.00176 | 1.38E-08 |
| rs9388490 | T | C | 0.00972 | 0.00171 | 1.43E-08 |
| rs9557378 | A | G | -0.01094 | 0.00193 | 1.45E-08 |
| rs9513416 | G | A | 0.01316 | 0.00232 | 1.46E-08 |
| rs2702575 | C | T | 0.00989 | 0.00175 | 1.56E-08 |
| rs1335482 | C | T | -0.0096 | 0.0017 | 1.63E-08 |
| rs28513882 | G | A | 0.01246 | 0.00221 | 1.63E-08 |
| rs2182505 | T | C | 0.01086 | 0.00192 | 1.64E-08 |
| rs28514598 | G | A | -0.01003 | 0.00178 | 1.67E-08 |
| rs818415 | T | G | -0.01235 | 0.00219 | 1.72E-08 |
| rs11623285 | G | T | 0.01413 | 0.00251 | 1.72E-08 |
| rs62172885 | T | C | -0.01012 | 0.00179 | 1.73E-08 |
| rs10402747 | C | T | -0.0097 | 0.00172 | 1.73E-08 |
| rs9386787 | A | G | -0.00958 | 0.0017 | 1.82E-08 |
| rs72686126 | C | T | 0.01862 | 0.00331 | 1.88E-08 |
| rs622169 | C | T | -0.00999 | 0.00178 | 1.89E-08 |
| rs2002058 | C | T | 0.01211 | 0.00216 | 1.97E-08 |
| rs2195086 | T | G | 0.01282 | 0.00228 | 2.00E-08 |
| rs12778624 | T | G | 0.01099 | 0.00196 | 2.02E-08 |
| rs7943853 | C | T | -0.01128 | 0.00201 | 2.06E-08 |
| rs1007731 | C | A | 0.0151 | 0.00269 | 2.07E-08 |
| rs2283076 | A | G | 0.01143 | 0.00204 | 2.07E-08 |
| rs75708852 | A | C | -0.02792 | 0.00498 | 2.09E-08 |
| rs1364626 | C | T | -0.00956 | 0.00171 | 2.15E-08 |
| rs2141277 | A | G | 0.00952 | 0.0017 | 2.16E-08 |
| rs4895650 | T | C | 0.00965 | 0.00172 | 2.16E-08 |
| rs10145520 | T | G | -0.01191 | 0.00213 | 2.17E-08 |
| rs62439690 | G | A | 0.01087 | 0.00194 | 2.18E-08 |
| rs143386970 | T | C | 0.016 | 0.00286 | 2.20E-08 |
| rs563954 | A | G | -0.00976 | 0.00175 | 2.29E-08 |
| rs4144624 | C | T | 0.01338 | 0.00239 | 2.30E-08 |
| rs1167827 | G | A | -0.00969 | 0.00173 | 2.31E-08 |
| rs74701752 | G | T | -0.01591 | 0.00285 | 2.38E-08 |
| rs11725086 | T | C | 0.00973 | 0.00174 | 2.40E-08 |
| rs55641816 | T | C | -0.01462 | 0.00262 | 2.40E-08 |
| rs112687095 | G | A | -0.01325 | 0.00238 | 2.42E-08 |
| rs12716848 | A | G | -0.00969 | 0.00174 | 2.43E-08 |
| rs6440008 | T | C | -0.00976 | 0.00175 | 2.44E-08 |
| rs1381247 | C | T | -0.01013 | 0.00182 | 2.46E-08 |
| rs2787101 | C | T | -0.00968 | 0.00174 | 2.50E-08 |
| rs16854920 | T | C | -0.01007 | 0.00181 | 2.51E-08 |
| rs7928622 | T | A | 0.01011 | 0.00181 | 2.52E-08 |
| rs62155873 | T | C | -0.01441 | 0.00259 | 2.58E-08 |
| rs6535149 | T | C | -0.01051 | 0.00189 | 2.62E-08 |
| rs35104491 | A | G | 0.01218 | 0.00219 | 2.62E-08 |
| rs1947114 | G | A | 0.01071 | 0.00192 | 2.64E-08 |
| rs77835879 | A | G | 0.01601 | 0.00288 | 2.68E-08 |
| rs2554835 | G | A | -0.00974 | 0.00175 | 2.69E-08 |
| rs277828 | C | A | 0.01091 | 0.00196 | 2.71E-08 |
| rs10616 | T | C | 0.0103 | 0.00185 | 2.74E-08 |
| rs1408284 | C | G | -0.01388 | 0.0025 | 2.74E-08 |
| rs7928017 | C | A | -0.00953 | 0.00172 | 2.83E-08 |
| rs62174974 | G | A | 0.01191 | 0.00215 | 2.83E-08 |
| rs72972965 | C | A | -0.01015 | 0.00183 | 2.84E-08 |
| rs11681861 | G | T | -0.01435 | 0.00259 | 2.88E-08 |
| rs28661002 | T | C | 0.01099 | 0.00198 | 2.89E-08 |
| rs12364080 | T | C | -0.01389 | 0.0025 | 2.89E-08 |
| rs795230 | T | C | 0.00952 | 0.00172 | 2.97E-08 |
| rs2336721 | C | T | -0.00996 | 0.0018 | 3.05E-08 |
| rs1566504 | T | C | 0.01126 | 0.00203 | 3.05E-08 |
| rs1960603 | C | G | 0.01096 | 0.00198 | 3.07E-08 |
| rs4858670 | C | T | 0.0101 | 0.00183 | 3.22E-08 |
| rs2589091 | G | A | 0.00949 | 0.00172 | 3.26E-08 |
| rs925161 | G | C | 0.00941 | 0.0017 | 3.26E-08 |
| rs2898191 | A | C | 0.01041 | 0.00188 | 3.29E-08 |
| rs73874335 | C | T | 0.0199 | 0.00361 | 3.40E-08 |
| rs59953820 | A | T | -0.01595 | 0.00289 | 3.50E-08 |
| rs10853455 | T | G | -0.01163 | 0.00211 | 3.61E-08 |
| rs11627087 | A | G | 0.01788 | 0.00325 | 3.71E-08 |
| rs4904523 | G | A | 0.00936 | 0.0017 | 3.71E-08 |
| rs75755471 | A | G | -0.01891 | 0.00344 | 3.79E-08 |
| rs72802200 | A | G | 0.04604 | 0.00838 | 3.87E-08 |
| rs12273435 | G | A | 0.01152 | 0.0021 | 3.96E-08 |
| rs13091704 | A | C | -0.0108 | 0.00197 | 4.11E-08 |
| rs1758747 | A | G | 0.0101 | 0.00184 | 4.13E-08 |
| rs273438 | G | A | 0.00937 | 0.00171 | 4.15E-08 |
| rs7278859 | A | T | -0.01013 | 0.00185 | 4.15E-08 |
| rs1918394 | C | T | -0.01261 | 0.0023 | 4.15E-08 |
| rs12030427 | G | A | -0.01085 | 0.00198 | 4.34E-08 |
| rs4766424 | G | C | -0.0141 | 0.00258 | 4.43E-08 |
| rs4733264 | C | G | -0.00954 | 0.00174 | 4.53E-08 |
| rs1461515 | G | A | -0.0093 | 0.0017 | 4.55E-08 |
| rs6871635 | A | G | -0.00946 | 0.00173 | 4.63E-08 |
| rs34410 | G | C | 0.00931 | 0.0017 | 4.70E-08 |
| rs10433551 | A | G | 0.01448 | 0.00265 | 4.73E-08 |
| rs535307 | A | G | 0.01004 | 0.00184 | 4.73E-08 |
| rs9384679 | T | C | -0.00959 | 0.00176 | 4.88E-08 |
| rs7016302 | G | C | 0.01243 | 0.00228 | 4.98E-08 |

| **Cognitive Ability instruments** (r2=0.01; kb:10,000;p<=5e-08) | | | | | |
| --- | --- | --- | --- | --- | --- |
| SNP | A1 | A2 | Beta | SE | P |
| rs10917152 | T | C | 0.024213 | 0.004049 | 2.23E-09 |
| rs7546297 | A | G | -0.01998 | 0.002818 | 1.33E-12 |
| rs12035012 | A | C | -0.02699 | 0.003312 | 3.68E-16 |
| rs3791134 | A | G | 0.016237 | 0.002839 | 1.07E-08 |
| rs4660749 | T | G | -0.02884 | 0.004446 | 8.72E-11 |
| rs1831539 | T | C | -0.0172 | 0.002762 | 4.72E-10 |
| rs2420551 | A | T | -0.0286 | 0.004344 | 4.60E-11 |
| rs12124523 | T | C | 0.032567 | 0.004887 | 2.67E-11 |
| rs3128341 | T | C | -0.03173 | 0.003417 | 1.63E-20 |
| rs6668048 | T | C | -0.02146 | 0.002734 | 4.24E-15 |
| rs9324380 | C | G | 0.02378 | 0.004267 | 2.50E-08 |
| rs11804556 | A | G | 0.034049 | 0.005481 | 5.24E-10 |
| rs1528204 | T | C | -0.01819 | 0.002774 | 5.47E-11 |
| rs1144593 | A | G | -0.01911 | 0.002987 | 1.57E-10 |
| rs112780312 | A | G | -0.01828 | 0.003099 | 3.66E-09 |
| rs34320898 | C | G | 0.022869 | 0.003844 | 2.70E-09 |
| rs199928 | T | C | 0.020051 | 0.003624 | 3.15E-08 |
| rs2678210 | T | C | 0.018786 | 0.003046 | 6.97E-10 |
| rs10779271 | A | G | 0.016375 | 0.002925 | 2.17E-08 |
| rs12470949 | T | C | -0.01717 | 0.003021 | 1.32E-08 |
| rs967569 | T | C | -0.01798 | 0.002927 | 8.21E-10 |
| rs2955280 | T | C | -0.01491 | 0.002734 | 4.90E-08 |
| rs62131236 | T | C | -0.01825 | 0.003342 | 4.80E-08 |
| rs7557525 | T | C | 0.015927 | 0.002902 | 4.04E-08 |
| rs58593843 | A | G | -0.02768 | 0.004652 | 2.67E-09 |
| rs10189857 | A | G | 0.018995 | 0.00275 | 4.91E-12 |
| rs2576835 | A | G | -0.01941 | 0.003206 | 1.40E-09 |
| rs4852252 | T | C | -0.02079 | 0.002747 | 3.84E-14 |
| rs11898362 | A | G | -0.01798 | 0.003008 | 2.25E-09 |
| rs11678106 | T | C | 0.016086 | 0.002745 | 4.62E-09 |
| rs2309812 | T | C | 0.022835 | 0.002845 | 9.95E-16 |
| rs60262711 | T | C | 0.015949 | 0.002825 | 1.65E-08 |
| rs2558096 | T | G | -0.01563 | 0.002774 | 1.74E-08 |
| rs10189912 | A | G | -0.01934 | 0.002853 | 1.22E-11 |
| rs3106666 | A | G | -0.01659 | 0.00278 | 2.42E-09 |
| rs6436555 | A | C | 0.01905 | 0.002746 | 3.99E-12 |
| rs10192369 | A | G | -0.01605 | 0.002744 | 4.91E-09 |
| rs62194171 | T | G | -0.01544 | 0.002831 | 4.95E-08 |
| rs1267042 | T | C | -0.01654 | 0.002996 | 3.40E-08 |
| rs2268894 | T | C | 0.020785 | 0.002749 | 3.98E-14 |
| rs3956504 | A | C | 0.017902 | 0.003052 | 4.47E-09 |
| rs13421971 | A | T | 0.01738 | 0.002893 | 1.88E-09 |
| rs3749034 | A | G | -0.01926 | 0.003322 | 6.74E-09 |
| rs62181012 | T | C | 0.021146 | 0.003511 | 1.73E-09 |
| rs62198803 | A | G | 0.019065 | 0.003225 | 3.40E-09 |
| rs7573001 | C | G | -0.01625 | 0.00286 | 1.32E-08 |
| rs1455344 | A | G | -0.01618 | 0.002769 | 5.20E-09 |
| rs35731967 | T | C | 0.021838 | 0.003658 | 2.38E-09 |
| rs13024268 | A | G | -0.01666 | 0.002879 | 7.15E-09 |
| rs73139272 | T | G | -0.02484 | 0.004057 | 9.24E-10 |
| rs6550835 | A | G | -0.02481 | 0.002929 | 2.44E-17 |
| rs1589652 | A | G | 0.017094 | 0.002759 | 5.82E-10 |
| rs2352974 | T | C | -0.03084 | 0.002751 | 3.69E-29 |
| rs4687625 | T | C | 0.019343 | 0.002748 | 1.92E-12 |
| rs4485754 | A | G | 0.018878 | 0.00334 | 1.59E-08 |
| rs11720523 | A | C | 0.018326 | 0.002773 | 3.89E-11 |
| rs6770622 | A | G | -0.04496 | 0.006864 | 5.76E-11 |
| rs7652296 | A | G | 0.016533 | 0.002799 | 3.51E-09 |
| rs3860537 | T | C | 0.018854 | 0.003402 | 2.99E-08 |
| rs13071190 | T | C | 0.018095 | 0.002917 | 5.55E-10 |
| rs59142272 | A | G | 0.022696 | 0.003685 | 7.32E-10 |
| rs10804681 | A | T | 0.021055 | 0.003797 | 2.94E-08 |
| rs12646225 | T | C | 0.025128 | 0.004215 | 2.51E-09 |
| rs2295499 | T | C | -0.0164 | 0.002753 | 2.59E-09 |
| rs4484297 | C | G | 0.018267 | 0.00316 | 7.45E-09 |
| rs11932971 | T | C | 0.027484 | 0.00381 | 5.46E-13 |
| rs34811474 | A | G | 0.028996 | 0.003594 | 7.15E-16 |
| rs67482514 | C | G | -0.01786 | 0.003229 | 3.21E-08 |
| rs6819372 | A | G | -0.0198 | 0.002729 | 4.02E-13 |
| rs1972860 | A | G | -0.01756 | 0.00293 | 2.09E-09 |
| rs4459994 | A | C | 0.018552 | 0.00329 | 1.71E-08 |
| rs34592089 | A | G | -0.05699 | 0.006462 | 1.15E-18 |
| rs2726491 | A | G | -0.02828 | 0.002857 | 4.17E-23 |
| rs6840804 | A | G | -0.01659 | 0.002966 | 2.25E-08 |
| rs6535809 | A | G | 0.019647 | 0.002734 | 6.65E-13 |
| rs17826816 | A | G | 0.018375 | 0.003257 | 1.68E-08 |
| rs1840847 | A | G | 0.016342 | 0.002883 | 1.44E-08 |
| rs75973558 | A | G | 0.025636 | 0.004465 | 9.42E-09 |
| rs13165296 | A | C | 0.019636 | 0.00352 | 2.44E-08 |
| rs36033 | T | C | 0.015969 | 0.002788 | 1.02E-08 |
| rs1812587 | T | G | -0.01734 | 0.002767 | 3.68E-10 |
| rs80170948 | T | G | 0.045381 | 0.007378 | 7.69E-10 |
| rs34316 | A | C | 0.021049 | 0.002767 | 2.82E-14 |
| rs166820 | A | G | 0.024334 | 0.003599 | 1.37E-11 |
| rs4308464 | C | G | -0.01837 | 0.002853 | 1.22E-10 |
| rs76160968 | A | G | -0.04171 | 0.007252 | 8.84E-09 |
| rs10477894 | A | G | -0.01621 | 0.002908 | 2.49E-08 |
| rs1438660 | A | T | 0.015846 | 0.002874 | 3.52E-08 |
| rs1145123 | T | C | 0.020557 | 0.002772 | 1.20E-13 |
| rs405321 | A | G | -0.01643 | 0.002975 | 3.32E-08 |
| rs4463213 | A | G | 0.019065 | 0.002732 | 3.00E-12 |
| rs31768 | A | T | 0.018177 | 0.003054 | 2.65E-09 |
| rs6860963 | T | C | 0.020262 | 0.003476 | 5.57E-09 |
| rs2450333 | A | G | -0.01883 | 0.002799 | 1.73E-11 |
| rs9503599 | T | C | -0.01711 | 0.002785 | 8.05E-10 |
| rs566237 | A | G | -0.01872 | 0.002935 | 1.82E-10 |
| rs6459098 | T | C | -0.01615 | 0.002933 | 3.67E-08 |
| rs6903716 | A | G | 0.017758 | 0.002975 | 2.39E-09 |
| rs1233578 | A | G | -0.0238 | 0.003903 | 1.08E-09 |
| rs1280049 | A | C | 0.014993 | 0.002729 | 3.92E-08 |
| rs12190777 | A | G | 0.01703 | 0.003092 | 3.63E-08 |
| rs1906252 | A | C | 0.031662 | 0.002741 | 7.48E-31 |
| rs3823036 | T | C | -0.01899 | 0.002929 | 9.11E-11 |
| rs9384679 | T | C | -0.02672 | 0.002783 | 7.94E-22 |
| rs13212044 | T | G | -0.01837 | 0.003242 | 1.46E-08 |
| rs287879 | A | G | -0.01887 | 0.003075 | 8.47E-10 |
| rs4725065 | A | G | -0.01653 | 0.002736 | 1.52E-09 |
| rs115064 | T | C | 0.016096 | 0.002814 | 1.07E-08 |
| rs1580019 | A | T | 0.016257 | 0.002876 | 1.59E-08 |
| rs799444 | T | C | 0.018415 | 0.002759 | 2.48E-11 |
| rs13223152 | A | G | 0.017645 | 0.002784 | 2.34E-10 |
| rs56150095 | A | C | -0.02197 | 0.002747 | 1.28E-15 |
| rs12535854 | C | G | -0.01823 | 0.002953 | 6.73E-10 |
| rs2402857 | A | G | 0.015446 | 0.00278 | 2.76E-08 |
| rs4731392 | A | G | -0.02174 | 0.002975 | 2.69E-13 |
| rs1043595 | A | G | 0.018957 | 0.003123 | 1.27E-09 |
| rs1362739 | A | C | 0.020945 | 0.002734 | 1.83E-14 |
| rs13253386 | T | G | -0.02013 | 0.002748 | 2.37E-13 |
| rs1473634 | A | G | -0.01809 | 0.002978 | 1.25E-09 |
| rs10954779 | T | C | -0.01638 | 0.002762 | 3.04E-09 |
| rs13276212 | T | G | 0.015071 | 0.002755 | 4.48E-08 |
| rs2920940 | T | C | -0.02474 | 0.003252 | 2.76E-14 |
| rs2111490 | A | G | 0.015491 | 0.002753 | 1.83E-08 |
| rs1106761 | A | G | -0.01775 | 0.002899 | 9.12E-10 |
| rs4976976 | A | G | 0.017317 | 0.002777 | 4.53E-10 |
| rs2721173 | T | C | -0.01623 | 0.002734 | 2.89E-09 |
| rs11793831 | T | G | 0.027834 | 0.002804 | 3.25E-23 |
| rs702222 | T | C | -0.01983 | 0.002872 | 5.02E-12 |
| rs28620532 | A | G | -0.01635 | 0.002889 | 1.51E-08 |
| rs1057687 | A | G | -0.01953 | 0.003481 | 2.02E-08 |
| rs913264 | T | C | 0.019725 | 0.003026 | 7.09E-11 |
| rs2987390 | C | G | -0.0178 | 0.003123 | 1.19E-08 |
| rs7069887 | A | C | 0.022532 | 0.003898 | 7.44E-09 |
| rs2393967 | A | C | -0.01871 | 0.002963 | 2.70E-10 |
| rs1891273 | T | C | 0.015414 | 0.002786 | 3.17E-08 |
| rs1408579 | T | C | 0.016049 | 0.002748 | 5.23E-09 |
| rs3740422 | C | G | -0.0241 | 0.002912 | 1.25E-16 |
| rs3896224 | A | G | -0.01531 | 0.002772 | 3.29E-08 |
| rs35608616 | A | G | -0.01809 | 0.002937 | 7.33E-10 |
| rs7921305 | A | G | 0.018192 | 0.00323 | 1.77E-08 |
| rs11605348 | A | G | -0.01661 | 0.002896 | 9.73E-09 |
| rs7941785 | A | G | 0.015512 | 0.002841 | 4.75E-08 |
| rs2373353 | A | G | -0.01632 | 0.002887 | 1.56E-08 |
| rs2508713 | A | T | 0.016531 | 0.002841 | 5.92E-09 |
| rs7116046 | T | C | 0.015707 | 0.002842 | 3.27E-08 |
| rs2885208 | T | C | 0.018939 | 0.003464 | 4.58E-08 |
| rs17128425 | A | T | 0.025557 | 0.004544 | 1.87E-08 |
| rs329672 | T | C | 0.01743 | 0.002853 | 1.00E-09 |
| rs55754731 | T | C | 0.021369 | 0.003675 | 6.06E-09 |
| rs1054442 | A | C | -0.02146 | 0.002816 | 2.52E-14 |
| rs1962047 | A | G | -0.01953 | 0.002863 | 8.89E-12 |
| rs6539284 | T | C | -0.01948 | 0.002827 | 5.56E-12 |
| rs7312919 | C | G | 0.018146 | 0.002915 | 4.83E-10 |
| rs1727307 | A | G | 0.017817 | 0.003007 | 3.10E-09 |
| rs9569206 | A | G | -0.01541 | 0.002824 | 4.85E-08 |
| rs3843954 | C | G | -0.02076 | 0.003343 | 5.31E-10 |
| rs9516855 | A | G | 0.033427 | 0.006096 | 4.19E-08 |
| rs2478286 | C | G | -0.02579 | 0.003127 | 1.64E-16 |
| rs8006700 | A | T | -0.01823 | 0.00293 | 4.96E-10 |
| rs176217 | T | C | 0.026141 | 0.003989 | 5.64E-11 |
| rs971681 | T | C | -0.01675 | 0.002807 | 2.44E-09 |
| rs2239647 | A | C | -0.02054 | 0.002766 | 1.14E-13 |
| rs11622558 | T | C | -0.01751 | 0.002824 | 5.66E-10 |
| rs35760956 | A | G | 0.019798 | 0.002823 | 2.35E-12 |
| rs17106817 | T | C | 0.016911 | 0.003025 | 2.26E-08 |
| rs1007934 | A | G | 0.016077 | 0.002806 | 1.00E-08 |
| rs17698580 | T | C | 0.019033 | 0.003177 | 2.09E-09 |
| rs2071407 | T | C | -0.02197 | 0.002859 | 1.52E-14 |
| rs11634187 | T | G | 0.022032 | 0.003857 | 1.12E-08 |
| rs55881236 | T | C | -0.01541 | 0.002794 | 3.48E-08 |
| rs7172979 | T | G | 0.060634 | 0.009084 | 2.47E-11 |
| rs72739469 | T | C | -0.03436 | 0.005648 | 1.18E-09 |
| rs8025964 | A | G | 0.017031 | 0.002749 | 5.78E-10 |
| rs1369429 | T | C | 0.017633 | 0.002896 | 1.15E-09 |
| rs11076962 | T | C | 0.016936 | 0.003042 | 2.57E-08 |
| rs11646221 | T | G | 0.017735 | 0.002772 | 1.57E-10 |
| rs72774059 | A | C | 0.02713 | 0.00459 | 3.41E-09 |
| rs2457192 | A | C | -0.01975 | 0.003131 | 2.84E-10 |
| rs62029752 | A | G | 0.019616 | 0.003149 | 4.68E-10 |
| rs72773563 | A | G | -0.02181 | 0.003773 | 7.40E-09 |
| rs9788857 | A | C | -0.02024 | 0.003495 | 7.03E-09 |
| rs34172651 | T | C | -0.0211 | 0.002962 | 1.06E-12 |
| rs2008514 | A | G | -0.02868 | 0.002799 | 1.25E-24 |
| rs2647995 | T | C | -0.01975 | 0.003044 | 8.68E-11 |
| rs8054299 | C | G | -0.02301 | 0.002927 | 3.84E-15 |
| rs12446238 | A | G | 0.016054 | 0.002742 | 4.80E-09 |
| rs9888986 | A | G | -0.0235 | 0.004262 | 3.52E-08 |
| rs7196032 | T | C | 0.015242 | 0.002786 | 4.47E-08 |
| rs8051038 | A | G | 0.018924 | 0.003146 | 1.78E-09 |
| rs2285640 | A | G | 0.017514 | 0.002765 | 2.38E-10 |
| rs4793161 | A | G | -0.01772 | 0.00325 | 4.97E-08 |
| rs17698176 | T | G | -0.02011 | 0.003566 | 1.70E-08 |
| rs11079849 | T | C | 0.016548 | 0.00296 | 2.26E-08 |
| rs16951547 | T | G | -0.01925 | 0.0031 | 5.31E-10 |
| rs66954617 | A | G | -0.02088 | 0.002834 | 1.72E-13 |
| rs71367283 | A | C | 0.055974 | 0.008746 | 1.55E-10 |
| rs6508220 | A | G | -0.02275 | 0.002737 | 9.56E-17 |
| rs76608582 | A | C | 0.042223 | 0.007581 | 2.55E-08 |
| rs17002025 | A | G | 0.025598 | 0.004261 | 1.89E-09 |
| rs10411958 | T | C | 0.016409 | 0.002759 | 2.71E-09 |
| rs2072490 | T | C | 0.016996 | 0.002745 | 5.93E-10 |
| rs7248006 | T | C | -0.01918 | 0.00282 | 1.05E-11 |
| rs144026674 | T | C | 0.041307 | 0.007468 | 3.19E-08 |
| rs889169 | A | G | 0.016071 | 0.002892 | 2.75E-08 |
| rs73068339 | C | G | 0.018858 | 0.003046 | 5.96E-10 |
| rs78084033 | A | C | -0.02288 | 0.004049 | 1.62E-08 |
| rs6019535 | A | G | 0.025105 | 0.002976 | 3.28E-17 |
| rs2836921 | A | G | 0.020346 | 0.002963 | 6.54E-12 |
| rs5753383 | A | G | 0.01591 | 0.002919 | 5.00E-08 |
| rs4396807 | C | G | -0.01573 | 0.002855 | 3.58E-08 |
| rs5750830 | A | C | 0.022891 | 0.003127 | 2.46E-13 |
| rs62236533 | A | G | 0.035363 | 0.004977 | 1.20E-12 |

**Table S2.** Harmonised instruments used in the MR analyses

| **Table S2a**.Harmonised instruments used in the MR analyses investigating the causal effect of genetic liability to ADHD on educational attainment | | | | | | | | | | | | | | |
| --- | --- | --- | --- | --- | --- | --- | --- | --- | --- | --- | --- | --- | --- | --- |
| **SNP** | **A1.ADHD** | **A2.ADHD** | **A1.EA** | **A2.EA** | **logOR.ADHD** | **SE.ADHD** | **P.ADHD** | **Beta.EA** | **SE.EA** | **P.EA** |  |  |  |  |
| rs10262192 | A | G | A | G | 0.074096 | 0.0135 | 3.66E-08 | -0.00426 | 0.0017 | 0.006107 |  |  |  |  |
| rs12410444 | A | G | A | G | 0.105297 | 0.0148 | 1.16E-12 | -0.01875 | 0.00185 | 1.93E-24 | *LD proxy for rs11420276 (r2=0.99) | | | |
| rs1427829 | A | G | A | G | 0.082197 | 0.0136 | 1.35E-09 | -0.0075 | 0.00171 | 5.77E-06 |  |  |  |  |
| rs212178 | A | G | A | G | -0.1171 | 0.0205 | 1.20E-08 | 0.01252 | 0.00274 | 2.45E-06 |  |  |  |  |
| rs4858241 | T | G | T | G | 0.082197 | 0.0143 | 8.17E-09 | -0.0052 | 0.00176 | 0.001566 |  |  |  |  |
| rs4916723 | A | C | A | C | -0.0778 | 0.0138 | 1.81E-08 | 0.01218 | 0.00172 | 7.14E-13 |  |  |  |  |
| rs74760947 | A | G | A | G | -0.17961 | 0.0317 | 1.39E-08 | -0.00278 | 0.00398 | 0.242435 |  |  |  |  |
| rs8039398 | T | C | T | C | -0.08 | 0.0135 | 2.99E-09 | 0.011 | 0.00171 | 6.27E-11 |  |  |  |  |
| rs11591402 | A | T | A | T | -0.0924 | 0.0164 | 1.76E-08 | 0.00598 | 0.00208 | 0.00202 | *Removed during harmonisation | | | |

| **Table S2b**.Harmonised instruments used in the MR analyses investigating the causal effect of genetic liability to ASD on educational attainment | | | | | | | | | | | | | | |
| --- | --- | --- | --- | --- | --- | --- | --- | --- | --- | --- | --- | --- | --- | --- |
| **SNP** | **A1.ASD** | **A2.ASD** | **A1.EA** | **A2.EA** | **logOR.ASD** | **SE.ASD** | **P.ASD** | **Beta.EA** | **SE.EA** | **P.EA** |  |  |  |  |
| rs112635299 | T | G | T | G | 0.220997 | 0.0432 | 3.04E-07 | 0.018 | 0.00621 | 0.00376 |  |  |  |  |
| rs1452075 | T | C | T | C | 0.080704 | 0.0155 | 2.07E-07 | 0.01226 | 0.00193 | 2.19E-10 |  |  |  |  |
| rs2391769 | A | G | A | G | -0.0769 | 0.0145 | 1.14E-07 | 0.00164 | 0.00178 | 0.356 |  |  |  |  |
| rs325485 | A | G | A | G | 0.072804 | 0.0143 | 3.25E-07 | -0.00732 | 0.00174 | 2.64E-05 |  |  |  |  |
| rs45595836 | T | C | T | C | 0.138996 | 0.0272 | 3.13E-07 | -0.00061 | 0.00344 | 0.86 |  |  |  |  |
| rs6701243 | A | C | A | C | 0.073501 | 0.0144 | 3.07E-07 | -0.00353 | 0.00182 | 0.0518 |  |  |  |  |
| rs910805 | A | G | A | G | -0.0957 | 0.016 | 2.04E-09 | 0.00082 | 0.00201 | 0.683 |  |  |  |  |
| rs10099100 | C | G | C | G | 0.084304 | 0.0147 | 1.07E-08 | 0.00023 | 0.0018 | 0.899 | *Removed during harmonisation | | | |

| **Table S2c.** Harmonised instruments used in the MR analyses investigating the causal effect of genetic liability to higher educational attainment on ADHD | | | | | | | | | | | | | | |
| --- | --- | --- | --- | --- | --- | --- | --- | --- | --- | --- | --- | --- | --- | --- |
| **SNP** | **A1.EA** | **A2.EA** | **A1.ADHD** | **A2.ADHD** | **Beta.EA** | **SE.EA** | **P.EA** | **logOR.ADHD** | **SE.ADHD** | **P.ADHD** |  |  |  |  |
| rs10060023 | C | T | C | T | -0.01178 | 0.00182 | 9.59E-11 | 0.002002 | 0.0149 | 0.8921 |  |  |  |  |
| rs1007731 | C | A | C | A | 0.0151 | 0.00269 | 2.07E-08 | -0.0177 | 0.0252 | 0.4824 |  |  |  |  |
| rs1008078 | T | C | T | C | -0.01738 | 0.00173 | 1.20E-23 | 0.030199 | 0.0137 | 0.02783 |  |  |  |  |
| rs10098073 | A | C | A | C | -0.01185 | 0.00171 | 3.71E-12 | -0.0199 | 0.0135 | 0.1403 |  |  |  |  |
| rs10120798 | A | G | A | G | -0.01028 | 0.00173 | 2.74E-09 | 0.048104 | 0.0136 | 0.000422 |  |  |  |  |
| rs10145520 | T | G | T | G | -0.01191 | 0.00213 | 2.17E-08 | 0.025405 | 0.0174 | 0.145 |  |  |  |  |
| rs10189857 | G | A | G | A | -0.01725 | 0.00171 | 6.70E-24 | 0.027505 | 0.0138 | 0.04592 |  |  |  |  |
| rs10191758 | A | G | A | G | -0.01631 | 0.00175 | 9.60E-21 | 0.001599 | 0.014 | 0.9081 |  |  |  |  |
| rs10205801 | A | G | A | G | -0.01053 | 0.00171 | 7.17E-10 | 0.049304 | 0.0136 | 0.000277 |  |  |  |  |
| rs10215082 | A | G | A | G | -0.01303 | 0.00172 | 3.33E-14 | 0.047399 | 0.0138 | 0.000598 |  |  |  |  |
| rs10402747 | C | T | C | T | -0.0097 | 0.00172 | 1.73E-08 | 0.011901 | 0.0146 | 0.4143 |  |  |  |  |
| rs1043209 | A | G | A | G | 0.01364 | 0.00174 | 4.22E-15 | 0.017899 | 0.0139 | 0.1954 |  |  |  |  |
| rs10433551 | A | G | A | G | 0.01448 | 0.00265 | 4.73E-08 | -0.0605 | 0.0228 | 0.007843 |  |  |  |  |
| rs10456918 | A | C | A | C | -0.01485 | 0.00224 | 3.67E-11 | 0.020704 | 0.0176 | 0.2392 |  |  |  |  |
| rs10460095 | G | A | G | A | 0.01066 | 0.00171 | 4.87E-10 | -0.0403 | 0.0137 | 0.003143 |  |  |  |  |
| rs10496091 | G | A | G | A | 0.01403 | 0.00188 | 7.47E-14 | -0.0125 | 0.0151 | 0.4076 |  |  |  |  |
| rs1051474 | T | C | T | C | -0.01301 | 0.00188 | 4.86E-12 | 0.0148 | 0.0151 | 0.327 |  |  |  |  |
| rs10519504 | T | G | T | G | 0.01381 | 0.00235 | 3.92E-09 | 0.019096 | 0.0188 | 0.3092 |  |  |  |  |
| rs1054442 | C | A | C | A | 0.01426 | 0.00177 | 8.42E-16 | -0.0496 | 0.0143 | 0.000517 |  |  |  |  |
| rs10616 | T | C | T | C | 0.0103 | 0.00185 | 2.74E-08 | 0.002597 | 0.0146 | 0.8581 |  |  |  |  |
| rs1061801 | A | G | A | G | -0.01354 | 0.0022 | 8.34E-10 | 0.026603 | 0.0178 | 0.1348 |  |  |  |  |
| rs10752262 | C | T | C | T | -0.01072 | 0.00174 | 7.80E-10 | 0.041604 | 0.0145 | 0.004139 |  |  |  |  |
| rs10765775 | A | G | A | G | 0.01488 | 0.00176 | 2.62E-17 | 0.003703 | 0.014 | 0.7926 |  |  |  |  |
| rs10795831 | T | G | T | G | -0.01223 | 0.00208 | 3.99E-09 | 0.006399 | 0.0162 | 0.6922 |  |  |  |  |
| rs10797055 | G | A | G | A | 0.00986 | 0.00172 | 1.04E-08 | -0.0141 | 0.0138 | 0.3083 |  |  |  |  |
| rs10810099 | A | G | A | G | -0.015 | 0.0019 | 3.31E-15 | 0.030898 | 0.0157 | 0.04912 |  |  |  |  |
| rs10853455 | T | G | T | G | -0.01163 | 0.00211 | 3.61E-08 | 0.023697 | 0.0164 | 0.1498 |  |  |  |  |
| rs10856785 | T | C | T | C | -0.01132 | 0.00192 | 3.83E-09 | 0.009396 | 0.0152 | 0.5377 |  |  |  |  |
| rs10879676 | T | C | T | C | -0.00982 | 0.00173 | 1.26E-08 | 0.008097 | 0.0135 | 0.5505 |  |  |  |  |
| rs10887801 | G | T | G | T | -0.01087 | 0.00171 | 2.27E-10 | 0.001001 | 0.0135 | 0.9403 |  |  |  |  |
| rs10906186 | C | T | C | T | -0.00991 | 0.00172 | 7.91E-09 | 0.019203 | 0.0136 | 0.1578 |  |  |  |  |
| rs10940921 | G | T | G | T | -0.01089 | 0.00177 | 7.00E-10 | 0.001802 | 0.0136 | 0.8953 |  |  |  |  |
| rs10984445 | G | A | G | A | -0.01158 | 0.00171 | 1.17E-11 | 0.004601 | 0.0136 | 0.7361 |  |  |  |  |
| rs10994777 | G | A | G | A | -0.0146 | 0.00232 | 3.36E-10 | 0.002804 | 0.0182 | 0.8774 |  |  |  |  |
| rs11019128 | T | C | T | C | -0.01123 | 0.00175 | 1.43E-10 | 0.037103 | 0.0139 | 0.007651 |  |  |  |  |
| rs11023749 | A | G | A | G | 0.01132 | 0.0018 | 2.96E-10 | 0.019597 | 0.0143 | 0.1704 |  |  |  |  |
| rs1105307 | A | G | A | G | -0.01173 | 0.00195 | 1.67E-09 | 0.019901 | 0.0153 | 0.194 |  |  |  |  |
| rs1106090 | A | G | A | G | 0.01173 | 0.00175 | 2.09E-11 | 0.0004 | 0.0139 | 0.9777 |  |  |  |  |
| rs11081529 | T | C | T | C | 0.01311 | 0.00186 | 1.82E-12 | -0.0163 | 0.015 | 0.2783 |  |  |  |  |
| rs11123818 | G | A | G | A | -0.02081 | 0.00175 | 1.72E-32 | 0.024498 | 0.014 | 0.07968 |  |  |  |  |
| rs111821073 | C | T | C | T | -0.01385 | 0.00237 | 4.85E-09 | 0.004701 | 0.0185 | 0.7989 |  |  |  |  |
| rs111852224 | T | C | T | C | 0.01663 | 0.00262 | 2.34E-10 | -0.0009 | 0.0214 | 0.9655 |  |  |  |  |
| rs11210934 | G | A | G | A | -0.01275 | 0.00194 | 4.84E-11 | 0.082903 | 0.0153 | 6.26E-08 |  |  |  |  |
| rs11223560 | G | A | G | A | -0.00995 | 0.00173 | 9.68E-09 | 0.032802 | 0.014 | 0.01868 |  |  |  |  |
| rs11259919 | A | G | A | G | -0.01093 | 0.0019 | 9.31E-09 | -0.0041 | 0.0151 | 0.7832 |  |  |  |  |
| rs113182709 | G | A | G | A | -0.03225 | 0.00567 | 1.29E-08 | 0.020101 | 0.054 | 0.7093 |  |  |  |  |
| rs1143770 | C | T | C | T | -0.01136 | 0.00172 | 4.31E-11 | 0.030799 | 0.0137 | 0.02452 |  |  |  |  |
| rs115438240 | G | T | G | T | 0.02162 | 0.00378 | 1.08E-08 | -0.0002 | 0.0305 | 0.995 |  |  |  |  |
| rs11601122 | G | A | G | A | -0.01947 | 0.0023 | 2.24E-17 | 0.036 | 0.0179 | 0.04458 |  |  |  |  |
| rs11620355 | G | A | G | A | -0.01756 | 0.003 | 4.77E-09 | 0.013501 | 0.0259 | 0.6027 |  |  |  |  |
| rs11623285 | G | T | G | T | 0.01413 | 0.00251 | 1.72E-08 | -0.0246 | 0.0229 | 0.2833 |  |  |  |  |
| rs11627087 | A | G | A | G | 0.01788 | 0.00325 | 3.71E-08 | -0.01521 | 0.0235 | 0.5187 |  |  |  |  |
| rs11635092 | A | G | A | G | -0.01231 | 0.00177 | 3.89E-12 | 0.002796 | 0.016 | 0.8599 |  |  |  |  |
| rs11646221 | T | G | T | G | 0.01144 | 0.00172 | 3.01E-11 | -0.0057 | 0.0137 | 0.6778 |  |  |  |  |
| rs11655029 | T | C | T | C | -0.0109 | 0.00183 | 2.84E-09 | -0.0265 | 0.0146 | 0.06964 |  |  |  |  |
| rs11657342 | A | G | A | G | 0.01404 | 0.00191 | 1.94E-13 | 0.0063 | 0.0167 | 0.7048 |  |  |  |  |
| rs11663602 | A | C | A | C | -0.01213 | 0.0019 | 1.64E-10 | 0.046101 | 0.0152 | 0.002445 |  |  |  |  |
| rs1167827 | G | A | G | A | -0.00969 | 0.00173 | 2.31E-08 | 0.029398 | 0.014 | 0.03624 |  |  |  |  |
| rs11678980 | G | A | G | A | 0.01744 | 0.00172 | 4.29E-24 | -0.0465 | 0.0162 | 0.004183 |  |  |  |  |
| rs11681861 | G | T | G | T | -0.01435 | 0.00259 | 2.88E-08 | 0.017095 | 0.0207 | 0.4094 |  |  |  |  |
| rs11694904 | T | C | T | C | 0.01215 | 0.00185 | 4.78E-11 | 0.002497 | 0.015 | 0.8659 |  |  |  |  |
| rs11703948 | A | G | A | G | -0.01737 | 0.00285 | 1.06E-09 | -0.0438 | 0.0225 | 0.05219 |  |  |  |  |
| rs11725086 | T | C | T | C | 0.00973 | 0.00174 | 2.40E-08 | -0.0187 | 0.0137 | 0.1721 |  |  |  |  |
| rs11732657 | A | G | A | G | -0.01274 | 0.00197 | 9.54E-11 | -0.0027 | 0.0159 | 0.8656 |  |  |  |  |
| rs11754551 | C | T | C | T | 0.01627 | 0.00272 | 2.08E-09 | 0.033298 | 0.0219 | 0.1288 |  |  |  |  |
| rs118093058 | T | G | T | G | 0.01525 | 0.00257 | 2.87E-09 | 0.006797 | 0.0213 | 0.7512 |  |  |  |  |
| rs11871429 | A | G | A | G | 0.01425 | 0.00202 | 1.92E-12 | -0.0034 | 0.0158 | 0.8305 |  |  |  |  |
| rs12030427 | G | A | G | A | -0.01085 | 0.00198 | 4.34E-08 | -0.0064 | 0.0169 | 0.7039 |  |  |  |  |
| rs12151248 | C | T | C | T | 0.01573 | 0.00271 | 6.75E-09 | 0.005002 | 0.0215 | 0.816 |  |  |  |  |
| rs12170452 | G | A | G | A | -0.01153 | 0.00171 | 1.40E-11 | -0.0023 | 0.0137 | 0.8691 |  |  |  |  |
| rs12273435 | G | A | G | A | 0.01152 | 0.0021 | 3.96E-08 | -0.0386 | 0.0183 | 0.03465 |  |  |  |  |
| rs12290350 | T | C | T | C | 0.013 | 0.00209 | 4.70E-10 | -0.0273 | 0.0167 | 0.1028 |  |  |  |  |
| rs12364080 | T | C | T | C | -0.01389 | 0.0025 | 2.89E-08 | -0.0138 | 0.0204 | 0.4982 |  |  |  |  |
| rs12375949 | T | C | T | C | -0.01447 | 0.00172 | 3.31E-17 | 0.028004 | 0.0137 | 0.04063 |  |  |  |  |
| rs12468040 | G | T | G | T | -0.01432 | 0.00175 | 2.46E-16 | 0.043795 | 0.0141 | 0.001872 |  |  |  |  |
| rs12503522 | C | T | C | T | 0.01125 | 0.00188 | 2.24E-09 | -0.011 | 0.0148 | 0.4585 |  |  |  |  |
| rs12515541 | G | T | G | T | 0.01135 | 0.00174 | 6.95E-11 | -0.0092 | 0.014 | 0.5129 |  |  |  |  |
| rs12574281 | A | C | A | C | -0.01077 | 0.00176 | 8.85E-10 | 0.013104 | 0.0143 | 0.3609 |  |  |  |  |
| rs12602286 | T | G | T | G | 0.01701 | 0.00255 | 2.37E-11 | 0.013903 | 0.0204 | 0.497 |  |  |  |  |
| rs12643771 | C | T | C | T | -0.01518 | 0.00184 | 1.61E-16 | 0.047396 | 0.0156 | 0.002392 |  |  |  |  |
| rs12646216 | T | C | T | C | 0.01062 | 0.00174 | 1.10E-09 | -0.0077 | 0.014 | 0.5828 |  |  |  |  |
| rs12646297 | G | A | G | A | 0.0111 | 0.00187 | 2.76E-09 | -0.0166 | 0.0152 | 0.2755 |  |  |  |  |
| rs12655753 | A | G | A | G | -0.02903 | 0.00509 | 1.18E-08 | 0.092096 | 0.0423 | 0.02954 |  |  |  |  |
| rs12682775 | T | C | T | C | -0.01187 | 0.00204 | 5.99E-09 | -0.0003 | 0.0164 | 0.9848 |  |  |  |  |
| rs12694681 | G | T | G | T | -0.01123 | 0.00183 | 9.06E-10 | 0.024703 | 0.0146 | 0.08975 |  |  |  |  |
| rs12712269 | C | T | C | T | 0.01063 | 0.00174 | 9.51E-10 | -0.0059 | 0.0139 | 0.6726 |  |  |  |  |
| rs12716848 | A | G | A | G | -0.00969 | 0.00174 | 2.43E-08 | 0.023199 | 0.0138 | 0.09294 |  |  |  |  |
| rs12724430 | T | G | T | G | 0.00979 | 0.0017 | 8.32E-09 | 0.015095 | 0.0135 | 0.2634 |  |  |  |  |
| rs12778624 | T | G | T | G | 0.01099 | 0.00196 | 2.02E-08 | -0.0325 | 0.0152 | 0.03255 |  |  |  |  |
| rs12908232 | A | G | A | G | 0.00975 | 0.0017 | 1.00E-08 | 0.006598 | 0.0135 | 0.6256 |  |  |  |  |
| rs1291818 | C | T | C | T | -0.01085 | 0.0017 | 1.78E-10 | 0.030903 | 0.0141 | 0.02873 |  |  |  |  |
| rs12926704 | A | G | A | G | -0.01737 | 0.00269 | 1.03E-10 | 0.020205 | 0.0208 | 0.3303 |  |  |  |  |
| rs13010288 | G | T | G | T | -0.01953 | 0.00252 | 1.04E-14 | 0.044297 | 0.0195 | 0.0234 |  |  |  |  |
| rs13010566 | A | C | A | C | -0.0106 | 0.0017 | 4.59E-10 | -0.0089 | 0.0135 | 0.5107 |  |  |  |  |
| rs13029509 | A | G | A | G | -0.01049 | 0.0017 | 7.17E-10 | 0.034498 | 0.0135 | 0.01088 |  |  |  |  |
| rs13035874 | A | G | A | G | -0.01114 | 0.00178 | 4.34E-10 | -0.0016 | 0.0143 | 0.9087 |  |  |  |  |
| rs13090388 | C | T | C | T | -0.02852 | 0.00184 | 4.29E-54 | 0.0009 | 0.0148 | 0.9523 |  |  |  |  |
| rs13091704 | A | C | A | C | -0.0108 | 0.00197 | 4.11E-08 | 0.021096 | 0.0159 | 0.1847 |  |  |  |  |
| rs13141210 | C | T | C | T | -0.01361 | 0.00172 | 2.26E-15 | 0.017604 | 0.0139 | 0.2042 |  |  |  |  |
| rs13145650 | C | T | C | T | 0.01918 | 0.00306 | 3.80E-10 | 0.020499 | 0.0242 | 0.3975 |  |  |  |  |
| rs13163062 | T | C | T | C | 0.01007 | 0.00172 | 4.71E-09 | -0.0006 | 0.0138 | 0.9639 |  |  |  |  |
| rs1334297 | A | G | A | G | 0.02449 | 0.00192 | 3.06E-37 | -0.0527 | 0.0148 | 0.000379 |  |  |  |  |
| rs1335482 | C | T | C | T | -0.0096 | 0.0017 | 1.63E-08 | 0.005797 | 0.0136 | 0.6686 |  |  |  |  |
| rs13422673 | C | T | C | T | 0.01201 | 0.0017 | 1.74E-12 | 0.0009 | 0.0135 | 0.9443 |  |  |  |  |
| rs1364626 | C | T | C | T | -0.00956 | 0.00171 | 2.15E-08 | -0.0009 | 0.0136 | 0.9452 |  |  |  |  |
| rs1381247 | C | T | C | T | -0.01013 | 0.00182 | 2.46E-08 | 0.034302 | 0.0157 | 0.02897 |  |  |  |  |
| rs1391438 | T | C | T | C | 0.0167 | 0.00183 | 5.79E-20 | -0.0124 | 0.0144 | 0.3905 |  |  |  |  |
| rs139244147 | G | A | G | A | 0.02095 | 0.00365 | 9.90E-09 | -0.0032 | 0.03 | 0.914 |  |  |  |  |
| rs1405876 | G | T | G | T | -0.01038 | 0.00177 | 4.40E-09 | 0.018001 | 0.014 | 0.2001 |  |  |  |  |
| rs1427298 | C | T | C | T | -0.0102 | 0.00172 | 3.28E-09 | -0.0056 | 0.0139 | 0.687 |  |  |  |  |
| rs143163770 | T | C | T | C | -0.01625 | 0.00258 | 3.22E-10 | 0.037498 | 0.0221 | 0.08925 |  |  |  |  |
| rs143386970 | T | C | T | C | 0.016 | 0.00286 | 2.20E-08 | -0.0078 | 0.0241 | 0.7473 |  |  |  |  |
| rs145590108 | G | T | G | T | -0.02232 | 0.00367 | 1.14E-09 | -0.0181 | 0.0259 | 0.4842 |  |  |  |  |
| rs1461515 | G | A | G | A | -0.0093 | 0.0017 | 4.55E-08 | 0.006803 | 0.0135 | 0.6152 |  |  |  |  |
| rs1475974 | C | T | C | T | 0.01269 | 0.0018 | 2.02E-12 | -0.0334 | 0.0145 | 0.02095 |  |  |  |  |
| rs152603 | G | A | G | A | 0.01019 | 0.00177 | 9.47E-09 | -0.0383 | 0.0144 | 0.007931 |  |  |  |  |
| rs1527878 | G | A | G | A | 0.01182 | 0.00199 | 3.08E-09 | -0.0405 | 0.0158 | 0.01032 |  |  |  |  |
| rs1550816 | C | T | C | T | -0.01107 | 0.00172 | 1.33E-10 | -0.0204 | 0.0141 | 0.1464 |  |  |  |  |
| rs1558727 | C | T | C | T | 0.01069 | 0.0017 | 3.09E-10 | -0.001 | 0.0138 | 0.9439 |  |  |  |  |
| rs1566085 | T | G | T | G | 0.01645 | 0.00171 | 6.90E-22 | 0.003404 | 0.0139 | 0.8039 |  |  |  |  |
| rs1566504 | T | C | T | C | 0.01126 | 0.00203 | 3.05E-08 | -0.0098 | 0.0165 | 0.554 |  |  |  |  |
| rs1569092 | A | G | A | G | 0.01807 | 0.00234 | 1.16E-14 | -0.008 | 0.0195 | 0.6801 |  |  |  |  |
| rs1569723 | A | C | A | C | 0.01168 | 0.00197 | 3.01E-09 | -0.0671 | 0.0156 | 1.71E-05 |  |  |  |  |
| rs1584469 | T | C | T | C | -0.01303 | 0.00185 | 2.10E-12 | 0.008999 | 0.0147 | 0.5428 |  |  |  |  |
| rs1599381 | A | G | A | G | 0.01002 | 0.0017 | 3.83E-09 | -0.0225 | 0.0136 | 0.09766 |  |  |  |  |
| rs1618725 | T | C | T | C | 0.01477 | 0.00174 | 2.22E-17 | 0.029403 | 0.0135 | 0.02883 |  |  |  |  |
| rs1620977 | G | A | G | A | -0.02046 | 0.00195 | 1.14E-25 | 0.0007 | 0.0164 | 0.9647 |  |  |  |  |
| rs1671770 | C | A | C | A | -0.01342 | 0.00223 | 1.91E-09 | 0.017696 | 0.018 | 0.3255 |  |  |  |  |
| rs16822665 | T | C | T | C | 0.01286 | 0.00185 | 3.57E-12 | -0.0052 | 0.0144 | 0.7162 |  |  |  |  |
| rs16854920 | T | C | T | C | -0.01007 | 0.00181 | 2.51E-08 | 0.033299 | 0.0153 | 0.02924 |  |  |  |  |
| rs16995054 | C | T | C | T | 0.0139 | 0.00208 | 2.52E-11 | 0.025605 | 0.016 | 0.1099 |  |  |  |  |
| rs17048855 | A | G | A | G | 0.01184 | 0.00179 | 3.27E-11 | -0.0176 | 0.0146 | 0.2263 |  |  |  |  |
| rs17060737 | C | T | C | T | -0.01192 | 0.0019 | 3.18E-10 | 0.017696 | 0.0149 | 0.2343 |  |  |  |  |
| rs17110109 | C | T | C | T | 0.01023 | 0.00175 | 4.71E-09 | -0.0145 | 0.0142 | 0.3064 |  |  |  |  |
| rs17126938 | T | C | T | C | -0.01536 | 0.0025 | 8.14E-10 | -0.0134 | 0.0197 | 0.4961 |  |  |  |  |
| rs17321729 | A | G | A | G | 0.01141 | 0.00196 | 6.06E-09 | -0.005 | 0.0161 | 0.7564 |  |  |  |  |
| rs17425572 | G | A | G | A | -0.01224 | 0.0017 | 6.89E-13 | 0.011799 | 0.0136 | 0.3858 |  |  |  |  |
| rs17489649 | A | G | A | G | 0.0139 | 0.00181 | 1.57E-14 | -0.016 | 0.0143 | 0.265 |  |  |  |  |
| rs17551064 | G | A | G | A | -0.01493 | 0.0023 | 8.62E-11 | 0.002303 | 0.0182 | 0.901 |  |  |  |  |
| rs17563464 | A | C | A | C | -0.01477 | 0.00212 | 2.89E-12 | 0.018203 | 0.0193 | 0.3457 |  |  |  |  |
| rs17565975 | A | G | A | G | -0.01142 | 0.00171 | 2.56E-11 | 0.015204 | 0.0138 | 0.2734 |  |  |  |  |
| rs17570033 | G | T | G | T | 0.01862 | 0.00283 | 4.65E-11 | -0.0076 | 0.0218 | 0.7278 |  |  |  |  |
| rs1758747 | A | G | A | G | 0.0101 | 0.00184 | 4.13E-08 | -0.0319 | 0.0149 | 0.03205 |  |  |  |  |
| rs17598675 | C | T | C | T | 0.01199 | 0.0017 | 1.75E-12 | -0.0195 | 0.0138 | 0.1577 |  |  |  |  |
| rs176218 | G | T | G | T | -0.01883 | 0.00215 | 1.85E-18 | 0.012204 | 0.0171 | 0.475 |  |  |  |  |
| rs17638867 | T | C | T | C | 0.01329 | 0.00222 | 2.11E-09 | -0.0106 | 0.0173 | 0.5408 |  |  |  |  |
| rs1827540 | G | A | G | A | -0.0106 | 0.0017 | 4.50E-10 | 0.013703 | 0.0134 | 0.3074 |  |  |  |  |
| rs1866823 | A | G | A | G | 0.01009 | 0.00171 | 3.81E-09 | -0.0013 | 0.0137 | 0.927 |  |  |  |  |
| rs1880692 | A | G | A | G | 0.01008 | 0.0017 | 3.17E-09 | -0.0151 | 0.0137 | 0.2685 |  |  |  |  |
| rs1892417 | C | T | C | T | -0.01732 | 0.00202 | 1.12E-17 | -0.0263 | 0.0159 | 0.09704 |  |  |  |  |
| rs1918394 | C | T | C | T | -0.01261 | 0.0023 | 4.15E-08 | 0.024498 | 0.0179 | 0.172 |  |  |  |  |
| rs192436652 | C | T | C | T | 0.03497 | 0.00545 | 1.35E-10 | -0.0187 | 0.0459 | 0.6832 |  |  |  |  |
| rs1925587 | C | T | C | T | -0.01016 | 0.00171 | 3.03E-09 | 0.027104 | 0.0136 | 0.04583 |  |  |  |  |
| rs1931259 | A | G | A | G | -0.01237 | 0.00211 | 4.30E-09 | -0.0007 | 0.0164 | 0.9636 |  |  |  |  |
| rs1947114 | G | A | G | A | 0.01071 | 0.00192 | 2.64E-08 | -0.043 | 0.0153 | 0.004817 |  |  |  |  |
| rs1952183 | G | A | G | A | 0.01055 | 0.0017 | 5.57E-10 | -0.0089 | 0.0139 | 0.5227 |  |  |  |  |
| rs1964927 | G | A | G | A | -0.01423 | 0.00177 | 9.90E-16 | 0.016597 | 0.0142 | 0.2444 |  |  |  |  |
| rs1979969 | G | T | G | T | 0.01208 | 0.00195 | 6.17E-10 | -0.0143 | 0.015 | 0.3402 |  |  |  |  |
| rs2002058 | C | T | C | T | 0.01211 | 0.00216 | 1.97E-08 | -0.0187 | 0.0174 | 0.2833 |  |  |  |  |
| rs2034670 | G | A | G | A | -0.01312 | 0.00214 | 8.09E-10 | 0.005204 | 0.0171 | 0.7596 |  |  |  |  |
| rs2052285 | A | G | A | G | 0.01123 | 0.00175 | 1.34E-10 | -0.0094 | 0.015 | 0.5296 |  |  |  |  |
| rs2067854 | A | G | A | G | 0.01477 | 0.00209 | 1.38E-12 | -0.0263 | 0.0167 | 0.1156 |  |  |  |  |
| rs2141277 | A | G | A | G | 0.00952 | 0.0017 | 2.16E-08 | -0.03381 | 0.0135 | 0.01249 |  |  |  |  |
| rs2179152 | C | T | C | T | 0.01455 | 0.00176 | 1.21E-16 | -0.0119 | 0.0138 | 0.3881 |  |  |  |  |
| rs2182505 | T | C | T | C | 0.01086 | 0.00192 | 1.64E-08 | 0.002896 | 0.015 | 0.8481 |  |  |  |  |
| rs2195086 | T | G | T | G | 0.01282 | 0.00228 | 2.00E-08 | 0.0161 | 0.0188 | 0.3935 |  |  |  |  |
| rs2220926 | T | C | T | C | -0.01055 | 0.00172 | 7.99E-10 | -0.002 | 0.0136 | 0.8838 |  |  |  |  |
| rs2245901 | A | G | A | G | -0.01403 | 0.00174 | 6.26E-16 | 0.023199 | 0.0138 | 0.09278 |  |  |  |  |
| rs2256965 | G | A | G | A | -0.01128 | 0.00176 | 1.59E-10 | 0.010202 | 0.0139 | 0.4636 |  |  |  |  |
| rs2283076 | A | G | A | G | 0.01143 | 0.00204 | 2.07E-08 | 0.018704 | 0.0166 | 0.26 |  |  |  |  |
| rs2287838 | A | G | A | G | -0.01152 | 0.00171 | 1.53E-11 | -0.0164 | 0.0138 | 0.2335 |  |  |  |  |
| rs2297600 | G | T | G | T | -0.01569 | 0.00223 | 2.16E-12 | 0.060196 | 0.0177 | 0.000678 |  |  |  |  |
| rs2302761 | T | C | T | C | 0.01354 | 0.00209 | 1.00E-10 | -0.0302 | 0.0167 | 0.07012 |  |  |  |  |
| rs2336721 | C | T | C | T | -0.00996 | 0.0018 | 3.05E-08 | 0.018602 | 0.0157 | 0.2367 |  |  |  |  |
| rs2343094 | A | G | A | G | 0.01029 | 0.00181 | 1.29E-08 | 0.007204 | 0.0142 | 0.6104 |  |  |  |  |
| rs2347526 | T | C | T | C | -0.01395 | 0.00179 | 6.84E-15 | 0.034701 | 0.0143 | 0.01535 |  |  |  |  |
| rs2365376 | C | A | C | A | -0.0107 | 0.00179 | 2.15E-09 | 0.029295 | 0.0144 | 0.04139 |  |  |  |  |
| rs2406253 | G | A | G | A | -0.01411 | 0.00216 | 6.40E-11 | 0.013501 | 0.0171 | 0.4316 |  |  |  |  |
| rs242093 | A | G | A | G | -0.01031 | 0.00172 | 2.07E-09 | 0.014297 | 0.0139 | 0.3051 |  |  |  |  |
| rs2447535 | A | G | A | G | -0.01181 | 0.00185 | 1.69E-10 | 0.022202 | 0.0146 | 0.1292 |  |  |  |  |
| rs2496482 | C | T | C | T | -0.01109 | 0.00177 | 4.04E-10 | 0.005797 | 0.0144 | 0.6868 |  |  |  |  |
| rs2554835 | G | A | G | A | -0.00974 | 0.00175 | 2.69E-08 | -0.0292 | 0.0145 | 0.04402 |  |  |  |  |
| rs2589091 | G | A | G | A | 0.00949 | 0.00172 | 3.26E-08 | 0.023105 | 0.0137 | 0.09228 |  |  |  |  |
| rs268120 | A | G | A | G | 0.01244 | 0.00196 | 2.13E-10 | -0.0343 | 0.0153 | 0.02487 |  |  |  |  |
| rs2702575 | C | T | C | T | 0.00989 | 0.00175 | 1.56E-08 | -0.0022 | 0.0139 | 0.8752 |  |  |  |  |
| rs2706762 | T | C | T | C | 0.01484 | 0.00246 | 1.59E-09 | 0.001898 | 0.0201 | 0.9243 |  |  |  |  |
| rs2725370 | T | C | T | C | -0.01536 | 0.00187 | 1.97E-16 | 0.032496 | 0.0148 | 0.02824 |  |  |  |  |
| rs273438 | G | A | G | A | 0.00937 | 0.00171 | 4.15E-08 | 0.012103 | 0.0135 | 0.3698 |  |  |  |  |
| rs277828 | C | A | C | A | 0.01091 | 0.00196 | 2.71E-08 | -0.0213 | 0.0162 | 0.1869 |  |  |  |  |
| rs2787101 | C | T | C | T | -0.00968 | 0.00174 | 2.50E-08 | 0.038595 | 0.0138 | 0.005238 |  |  |  |  |
| rs281302 | A | G | A | G | -0.0111 | 0.00172 | 9.84E-11 | 0.030597 | 0.0139 | 0.02709 |  |  |  |  |
| rs2819336 | T | C | T | C | 0.01828 | 0.00177 | 5.46E-25 | -0.089 | 0.0141 | 2.83E-10 |  |  |  |  |
| rs2820314 | A | C | A | C | 0.011 | 0.0018 | 9.34E-10 | -0.0371 | 0.0143 | 0.009695 |  |  |  |  |
| rs2833483 | C | T | C | T | 0.01974 | 0.00337 | 4.77E-09 | -0.0131 | 0.028 | 0.641 |  |  |  |  |
| rs28513670 | G | A | G | A | 0.01477 | 0.00225 | 5.06E-11 | -0.0224 | 0.0178 | 0.2085 |  |  |  |  |
| rs28513882 | G | A | G | A | 0.01246 | 0.00221 | 1.63E-08 | -0.0282 | 0.0179 | 0.1153 |  |  |  |  |
| rs28514598 | G | A | G | A | -0.01003 | 0.00178 | 1.67E-08 | 0.033505 | 0.0143 | 0.01898 |  |  |  |  |
| rs28661002 | T | C | T | C | 0.01099 | 0.00198 | 2.89E-08 | 0.035502 | 0.0157 | 0.02399 |  |  |  |  |
| rs2885198 | A | G | A | G | 0.01025 | 0.0017 | 1.81E-09 | -0.0148 | 0.0138 | 0.284 |  |  |  |  |
| rs2898191 | A | C | A | C | 0.01041 | 0.00188 | 3.29E-08 | -0.006 | 0.0147 | 0.6815 |  |  |  |  |
| rs2964197 | T | C | T | C | 0.01177 | 0.0017 | 4.70E-12 | -0.0218 | 0.0135 | 0.107 |  |  |  |  |
| rs2971970 | T | G | T | G | -0.01654 | 0.00207 | 1.25E-15 | 0.046196 | 0.0165 | 0.005098 |  |  |  |  |
| rs2998315 | G | A | G | A | 0.01269 | 0.00171 | 1.12E-13 | -0.0147 | 0.0141 | 0.2999 |  |  |  |  |
| rs301800 | T | C | T | C | 0.01516 | 0.00224 | 1.33E-11 | -0.0087 | 0.0182 | 0.6308 |  |  |  |  |
| rs3026996 | A | C | A | C | 0.01537 | 0.00199 | 1.05E-14 | 0.007899 | 0.0162 | 0.6262 |  |  |  |  |
| rs31940 | G | A | G | A | -0.01548 | 0.00246 | 3.24E-10 | 0.007498 | 0.0199 | 0.7067 |  |  |  |  |
| rs337637 | G | A | G | A | -0.01123 | 0.00177 | 2.11E-10 | 0.011901 | 0.0141 | 0.3964 |  |  |  |  |
| rs339054 | G | T | G | T | 0.0117 | 0.0017 | 5.90E-12 | -0.021 | 0.0136 | 0.1239 |  |  |  |  |
| rs34316 | C | A | C | A | -0.02016 | 0.00177 | 3.35E-30 | 0.046201 | 0.0139 | 0.000857 |  |  |  |  |
| rs34394051 | G | A | G | A | 0.01392 | 0.0024 | 6.20E-09 | -0.0437 | 0.0187 | 0.0191 |  |  |  |  |
| rs34485537 | T | C | T | C | 0.01075 | 0.00173 | 5.67E-10 | -0.0187 | 0.014 | 0.1826 |  |  |  |  |
| rs35039375 | G | A | G | A | -0.01983 | 0.00293 | 1.22E-11 | 0.001902 | 0.024 | 0.9361 |  |  |  |  |
| rs35104491 | A | G | A | G | 0.01218 | 0.00219 | 2.62E-08 | 0.031896 | 0.0171 | 0.06193 |  |  |  |  |
| rs35309068 | G | T | G | T | 0.01321 | 0.00171 | 1.15E-14 | -0.0211 | 0.0137 | 0.1239 |  |  |  |  |
| rs35316276 | C | T | C | T | -0.01173 | 0.00194 | 1.52E-09 | 0.008304 | 0.0157 | 0.5951 |  |  |  |  |
| rs35417702 | C | T | C | T | 0.01445 | 0.0017 | 1.93E-17 | -0.0581 | 0.0135 | 1.65E-05 |  |  |  |  |
| rs35475880 | G | T | G | T | 0.01511 | 0.00208 | 3.80E-13 | 0.011405 | 0.0167 | 0.4948 |  |  |  |  |
| rs35919256 | C | A | C | A | -0.01032 | 0.00177 | 5.56E-09 | 0.044903 | 0.0141 | 0.001447 |  |  |  |  |
| rs36083520 | C | T | C | T | 0.01629 | 0.00223 | 2.60E-13 | -0.0119 | 0.0179 | 0.5076 |  |  |  |  |
| rs36119825 | G | A | G | A | -0.01063 | 0.00171 | 4.82E-10 | 0.017502 | 0.0135 | 0.1963 |  |  |  |  |
| rs363096 | C | T | C | T | 0.01363 | 0.00172 | 2.04E-15 | -0.0356 | 0.0135 | 0.008441 |  |  |  |  |
| rs3781339 | T | C | T | C | -0.01247 | 0.00219 | 1.30E-08 | 0.034305 | 0.0173 | 0.04671 |  |  |  |  |
| rs3796348 | G | A | G | A | -0.01032 | 0.00175 | 3.66E-09 | 0.040801 | 0.0145 | 0.004865 |  |  |  |  |
| rs3809634 | A | G | A | G | -0.01058 | 0.00185 | 1.09E-08 | 0.031499 | 0.0146 | 0.03046 |  |  |  |  |
| rs3812281 | C | T | C | T | -0.01228 | 0.00174 | 1.58E-12 | 0.0229 | 0.0138 | 0.09719 |  |  |  |  |
| rs3890802 | A | G | A | G | -0.01133 | 0.00191 | 2.74E-09 | 0.021497 | 0.0148 | 0.1473 |  |  |  |  |
| rs3897821 | A | G | A | G | 0.01502 | 0.0018 | 8.25E-17 | -0.0234 | 0.0142 | 0.09846 |  |  |  |  |
| rs4073894 | A | G | A | G | 0.01524 | 0.00211 | 5.40E-13 | -0.0435 | 0.0172 | 0.01126 |  |  |  |  |
| rs4144624 | C | T | C | T | 0.01338 | 0.00239 | 2.30E-08 | -0.0255 | 0.0198 | 0.1974 |  |  |  |  |
| rs4328757 | T | C | T | C | 0.01067 | 0.00174 | 9.39E-10 | -0.0114 | 0.0137 | 0.406 |  |  |  |  |
| rs4352658 | T | C | T | C | -0.0212 | 0.00308 | 5.55E-12 | 0.055898 | 0.0255 | 0.02864 |  |  |  |  |
| rs4369924 | A | G | A | G | 0.01362 | 0.00234 | 5.82E-09 | -0.0094 | 0.0198 | 0.6362 |  |  |  |  |
| rs4382592 | T | G | T | G | -0.01636 | 0.00185 | 1.01E-18 | 0.037999 | 0.0145 | 0.008832 |  |  |  |  |
| rs4384309 | G | A | G | A | -0.0109 | 0.00172 | 2.52E-10 | 0.009899 | 0.0142 | 0.4832 |  |  |  |  |
| rs4442732 | G | A | G | A | -0.01063 | 0.00176 | 1.49E-09 | -0.0474 | 0.0138 | 0.000592 |  |  |  |  |
| rs4641552 | C | A | C | A | -0.01926 | 0.00336 | 1.02E-08 | 0.069404 | 0.0276 | 0.01204 |  |  |  |  |
| rs4652135 | C | A | C | A | -0.01219 | 0.0019 | 1.54E-10 | 0.010101 | 0.0149 | 0.499 |  |  |  |  |
| rs4675248 | G | A | G | A | 0.01004 | 0.00173 | 6.75E-09 | -0.0204 | 0.0139 | 0.1433 |  |  |  |  |
| rs4700393 | G | A | G | A | 0.02086 | 0.0017 | 1.51E-34 | -0.0072 | 0.0135 | 0.594 |  |  |  |  |
| rs4726070 | G | A | G | A | -0.01251 | 0.00174 | 5.95E-13 | 0.039698 | 0.0136 | 0.003669 |  |  |  |  |
| rs4731413 | G | A | G | A | -0.01211 | 0.00213 | 1.37E-08 | -0.0117 | 0.0179 | 0.5154 |  |  |  |  |
| rs4760687 | G | A | G | A | 0.01039 | 0.00183 | 1.30E-08 | -0.0395 | 0.0148 | 0.007393 |  |  |  |  |
| rs4778058 | C | T | C | T | 0.01017 | 0.0017 | 2.40E-09 | -0.0039 | 0.0137 | 0.7779 |  |  |  |  |
| rs4780563 | G | A | G | A | 0.01398 | 0.00245 | 1.12E-08 | -0.0348 | 0.0204 | 0.08784 |  |  |  |  |
| rs4787457 | G | A | G | A | -0.01741 | 0.00176 | 3.73E-23 | 0.0077 | 0.0139 | 0.5782 |  |  |  |  |
| rs4810227 | A | G | A | G | 0.01272 | 0.00175 | 3.57E-13 | -0.0057 | 0.0139 | 0.6822 |  |  |  |  |
| rs4839155 | T | G | T | G | 0.01251 | 0.002 | 3.94E-10 | -0.0205 | 0.0157 | 0.1915 |  |  |  |  |
| rs4846724 | G | A | G | A | -0.01018 | 0.0017 | 2.26E-09 | -0.0069 | 0.0135 | 0.6093 |  |  |  |  |
| rs4858670 | C | T | C | T | 0.0101 | 0.00183 | 3.22E-08 | -0.0104 | 0.0144 | 0.4706 |  |  |  |  |
| rs4860734 | A | G | A | G | -0.0114 | 0.00188 | 1.29E-09 | 0.006002 | 0.0153 | 0.6922 |  |  |  |  |
| rs4895650 | T | C | T | C | 0.00965 | 0.00172 | 2.16E-08 | 0.012195 | 0.014 | 0.3819 |  |  |  |  |
| rs4904523 | G | A | G | A | 0.00936 | 0.0017 | 3.71E-08 | -0.0068 | 0.0136 | 0.6143 |  |  |  |  |
| rs4964046 | G | A | G | A | 0.01053 | 0.00178 | 3.36E-09 | -0.0247 | 0.0142 | 0.08106 |  |  |  |  |
| rs4972400 | G | A | G | A | -0.01156 | 0.00181 | 1.70E-10 | -0.013 | 0.0149 | 0.3834 |  |  |  |  |
| rs4976445 | T | C | T | C | 0.012 | 0.00197 | 1.16E-09 | -0.0309 | 0.0156 | 0.04703 |  |  |  |  |
| rs535307 | A | G | A | G | 0.01004 | 0.00184 | 4.73E-08 | -0.007 | 0.0151 | 0.6425 |  |  |  |  |
| rs55641816 | T | C | T | C | -0.01462 | 0.00262 | 2.40E-08 | 0.036303 | 0.0193 | 0.06042 |  |  |  |  |
| rs56048629 | T | C | T | C | -0.01654 | 0.00176 | 6.76E-21 | 0.037903 | 0.0142 | 0.007581 |  |  |  |  |
| rs56194430 | T | C | T | C | -0.01514 | 0.00232 | 6.65E-11 | -0.0098 | 0.0202 | 0.628 |  |  |  |  |
| rs56330207 | A | G | A | G | 0.01184 | 0.00207 | 1.05E-08 | -0.024 | 0.0167 | 0.1511 |  |  |  |  |
| rs56391344 | A | G | A | G | 0.01571 | 0.00197 | 1.34E-15 | -0.034 | 0.0158 | 0.03098 |  |  |  |  |
| rs563954 | A | G | A | G | -0.00976 | 0.00175 | 2.29E-08 | 0.007502 | 0.0136 | 0.5791 |  |  |  |  |
| rs57016874 | C | T | C | T | -0.02902 | 0.00457 | 2.09E-10 | 0.057396 | 0.0418 | 0.1694 |  |  |  |  |
| rs5763431 | T | C | T | C | 0.01125 | 0.00176 | 1.53E-10 | 0.0118 | 0.0139 | 0.3928 |  |  |  |  |
| rs59123361 | A | G | A | G | -0.02094 | 0.00291 | 5.87E-13 | 0.035396 | 0.0236 | 0.1346 |  |  |  |  |
| rs59484001 | C | T | C | T | 0.02926 | 0.00391 | 7.42E-14 | -0.0714 | 0.0338 | 0.03487 |  |  |  |  |
| rs60096640 | G | A | G | A | -0.01574 | 0.00273 | 7.82E-09 | 0.041395 | 0.0208 | 0.04659 |  |  |  |  |
| rs6122735 | T | C | T | C | 0.0105 | 0.00174 | 1.49E-09 | -0.0252 | 0.0137 | 0.06574 |  |  |  |  |
| rs6123924 | A | G | A | G | 0.01528 | 0.00235 | 7.55E-11 | -0.0076 | 0.0183 | 0.6769 |  |  |  |  |
| rs613872 | G | T | G | T | 0.0175 | 0.00227 | 1.20E-14 | -0.0145 | 0.018 | 0.4204 |  |  |  |  |
| rs61757207 | G | A | G | A | -0.04941 | 0.00795 | 5.09E-10 | 0.032999 | 0.0665 | 0.6195 |  |  |  |  |
| rs61996546 | C | T | C | T | -0.00965 | 0.0017 | 1.34E-08 | -0.0061 | 0.0137 | 0.6585 |  |  |  |  |
| rs62142891 | G | A | G | A | -0.01121 | 0.00194 | 6.94E-09 | 0.022102 | 0.0155 | 0.1538 |  |  |  |  |
| rs62155873 | T | C | T | C | -0.01441 | 0.00259 | 2.58E-08 | 0.010099 | 0.0209 | 0.6284 |  |  |  |  |
| rs62157915 | T | C | T | C | -0.02091 | 0.00348 | 1.96E-09 | 0.0252 | 0.0276 | 0.3611 |  |  |  |  |
| rs62166492 | A | G | A | G | 0.02846 | 0.0034 | 6.03E-17 | -0.0416 | 0.0261 | 0.111 |  |  |  |  |
| rs62172885 | T | C | T | C | -0.01012 | 0.00179 | 1.73E-08 | 0.0161 | 0.0142 | 0.2555 |  |  |  |  |
| rs62174974 | G | A | G | A | 0.01191 | 0.00215 | 2.83E-08 | -0.0524 | 0.017 | 0.002071 |  |  |  |  |
| rs62177359 | A | C | A | C | -0.0304 | 0.00475 | 1.60E-10 | 0.036602 | 0.0331 | 0.2679 |  |  |  |  |
| rs62184480 | T | C | T | C | -0.01528 | 0.00191 | 1.28E-15 | 0.035 | 0.015 | 0.01961 |  |  |  |  |
| rs62190914 | T | C | T | C | 0.01001 | 0.00176 | 1.38E-08 | -0.0102 | 0.0139 | 0.4639 |  |  |  |  |
| rs62439690 | G | A | G | A | 0.01087 | 0.00194 | 2.18E-08 | -0.0067 | 0.0159 | 0.6763 |  |  |  |  |
| rs62444881 | C | T | C | T | -0.01815 | 0.00217 | 5.79E-17 | 0.062301 | 0.0172 | 0.000291 |  |  |  |  |
| rs635754 | A | G | A | G | 0.0134 | 0.00173 | 8.01E-15 | 0.033996 | 0.0136 | 0.01259 |  |  |  |  |
| rs6429082 | C | T | C | T | 0.01078 | 0.0017 | 2.37E-10 | -0.008 | 0.0135 | 0.553 |  |  |  |  |
| rs6436555 | C | A | C | A | -0.01012 | 0.0017 | 2.70E-09 | 0.003105 | 0.0137 | 0.8182 |  |  |  |  |
| rs6440008 | T | C | T | C | -0.00976 | 0.00175 | 2.44E-08 | 0.017604 | 0.0145 | 0.2243 |  |  |  |  |
| rs6457996 | C | T | C | T | -0.01014 | 0.0017 | 2.43E-09 | 0.036996 | 0.0137 | 0.007008 |  |  |  |  |
| rs6493265 | C | T | C | T | 0.01385 | 0.00174 | 1.70E-15 | -0.0286 | 0.0139 | 0.03908 |  |  |  |  |
| rs6513959 | A | G | A | G | 0.01177 | 0.00185 | 1.88E-10 | -0.0066 | 0.0148 | 0.6528 |  |  |  |  |
| rs6535149 | T | C | T | C | -0.01051 | 0.00189 | 2.62E-08 | 0.0063 | 0.0165 | 0.7016 |  |  |  |  |
| rs6557171 | C | T | C | T | 0.01567 | 0.00181 | 4.15E-18 | -0.0483 | 0.0146 | 0.000911 |  |  |  |  |
| rs6573552 | T | C | T | C | -0.01086 | 0.0017 | 1.62E-10 | -0.0089 | 0.0135 | 0.5083 |  |  |  |  |
| rs66568921 | G | T | G | T | 0.01565 | 0.00182 | 7.49E-18 | -0.0408 | 0.0141 | 0.003921 |  |  |  |  |
| rs66641143 | T | C | T | C | -0.03162 | 0.00486 | 8.04E-11 | 0.038201 | 0.0383 | 0.3193 |  |  |  |  |
| rs6666119 | A | G | A | G | -0.01269 | 0.00183 | 4.16E-12 | 0.016198 | 0.0146 | 0.2671 |  |  |  |  |
| rs66671632 | C | T | C | T | 0.01838 | 0.00254 | 4.59E-13 | -0.0251 | 0.021 | 0.2317 |  |  |  |  |
| rs6731373 | G | A | G | A | 0.01256 | 0.00181 | 3.47E-12 | -0.0084 | 0.0147 | 0.5698 |  |  |  |  |
| rs6736898 | A | G | A | G | 0.01032 | 0.00178 | 6.09E-09 | -0.0472 | 0.0141 | 0.000832 |  |  |  |  |
| rs6774533 | T | C | T | C | 0.0126 | 0.00187 | 1.73E-11 | 0.020205 | 0.0153 | 0.1866 |  |  |  |  |
| rs67885444 | T | C | T | C | 0.01406 | 0.00232 | 1.48E-09 | -0.0069 | 0.019 | 0.7174 |  |  |  |  |
| rs67890737 | C | A | C | A | 0.01141 | 0.00179 | 2.01E-10 | -0.037 | 0.0141 | 0.008449 |  |  |  |  |
| rs6805241 | C | T | C | T | -0.01413 | 0.00203 | 3.09E-12 | 0.030995 | 0.0163 | 0.05716 |  |  |  |  |
| rs6821231 | C | T | C | T | 0.01217 | 0.00197 | 6.06E-10 | -0.0343 | 0.0158 | 0.03013 |  |  |  |  |
| rs6871635 | A | G | A | G | -0.00946 | 0.00173 | 4.63E-08 | 0.032099 | 0.0141 | 0.02269 |  |  |  |  |
| rs6959891 | G | A | G | A | -0.01136 | 0.00189 | 1.74E-09 | 0.008597 | 0.0148 | 0.5624 |  |  |  |  |
| rs7012546 | C | T | C | T | -0.01009 | 0.00172 | 4.93E-09 | 0.009596 | 0.0137 | 0.4841 |  |  |  |  |
| rs702606 | T | C | T | C | 0.01427 | 0.0025 | 1.12E-08 | -0.0008 | 0.0211 | 0.9683 |  |  |  |  |
| rs7029718 | G | A | G | A | -0.02439 | 0.00174 | 1.85E-44 | 0.001601 | 0.014 | 0.9067 |  |  |  |  |
| rs710629 | A | G | A | G | 0.01053 | 0.00177 | 2.96E-09 | -0.0188 | 0.014 | 0.1783 |  |  |  |  |
| rs7108020 | C | A | C | A | -0.01094 | 0.00178 | 7.35E-10 | 0.0044 | 0.0144 | 0.7591 |  |  |  |  |
| rs7117878 | A | C | A | C | -0.01036 | 0.00182 | 1.18E-08 | 0.043902 | 0.0144 | 0.002307 |  |  |  |  |
| rs7136760 | A | G | A | G | 0.01018 | 0.00177 | 9.36E-09 | 0.003902 | 0.0143 | 0.7845 |  |  |  |  |
| rs7139165 | C | A | C | A | -0.01136 | 0.00195 | 5.29E-09 | 0.042 | 0.0146 | 0.003875 |  |  |  |  |
| rs71415374 | T | C | T | C | 0.02142 | 0.00307 | 2.93E-12 | -0.0097 | 0.0237 | 0.6841 |  |  |  |  |
| rs71432775 | A | G | A | G | 0.0113 | 0.00198 | 1.06E-08 | -0.0164 | 0.0153 | 0.2851 |  |  |  |  |
| rs71646142 | C | T | C | T | -0.01286 | 0.00217 | 3.11E-09 | 0.0003 | 0.0174 | 0.9855 |  |  |  |  |
| rs7188873 | A | G | A | G | 0.0106 | 0.00175 | 1.34E-09 | -0.0192 | 0.0138 | 0.1637 |  |  |  |  |
| rs7215889 | T | C | T | C | 0.01132 | 0.00196 | 7.06E-09 | -0.0168 | 0.0155 | 0.2788 |  |  |  |  |
| rs7233920 | A | G | A | G | -0.01315 | 0.00202 | 7.13E-11 | 0.028004 | 0.0163 | 0.08551 |  |  |  |  |
| rs72486027 | C | T | C | T | 0.01123 | 0.00197 | 1.25E-08 | 0.019897 | 0.0154 | 0.1963 |  |  |  |  |
| rs7257460 | T | C | T | C | 0.01145 | 0.00189 | 1.25E-09 | -0.0515 | 0.0147 | 0.000467 |  |  |  |  |
| rs72677177 | G | A | G | A | -0.0105 | 0.00174 | 1.55E-09 | -0.0037 | 0.0138 | 0.788 |  |  |  |  |
| rs72686126 | C | T | C | T | 0.01862 | 0.00331 | 1.88E-08 | 0.0142 | 0.0309 | 0.647 |  |  |  |  |
| rs72693550 | C | A | C | A | 0.01542 | 0.00234 | 4.57E-11 | 0.002603 | 0.0215 | 0.9032 |  |  |  |  |
| rs728054 | G | A | G | A | 0.01274 | 0.00177 | 6.65E-13 | -0.0188 | 0.0143 | 0.1878 |  |  |  |  |
| rs72828517 | C | T | C | T | 0.01836 | 0.00224 | 2.83E-16 | 0.038699 | 0.0179 | 0.0301 |  |  |  |  |
| rs72829857 | G | A | G | A | 0.01516 | 0.00202 | 5.39E-14 | -0.031 | 0.016 | 0.05191 |  |  |  |  |
| rs72840994 | G | T | G | T | 0.01247 | 0.00216 | 7.77E-09 | -0.0058 | 0.0172 | 0.737 |  |  |  |  |
| rs72972965 | C | A | C | A | -0.01015 | 0.00183 | 2.84E-08 | -0.024 | 0.0138 | 0.08152 |  |  |  |  |
| rs730384 | G | A | G | A | -0.01016 | 0.00171 | 3.01E-09 | 0.030098 | 0.0138 | 0.02863 |  |  |  |  |
| rs7321274 | G | A | G | A | -0.01275 | 0.00211 | 1.59E-09 | 0.019295 | 0.0164 | 0.2393 |  |  |  |  |
| rs73301698 | A | G | A | G | -0.01291 | 0.00208 | 5.81E-10 | 0.031101 | 0.0168 | 0.06469 |  |  |  |  |
| rs7332724 | T | C | T | C | -0.01149 | 0.00189 | 1.26E-09 | 0.036197 | 0.0149 | 0.01511 |  |  |  |  |
| rs73344830 | G | A | G | A | -0.0172 | 0.00172 | 1.95E-23 | 0.029995 | 0.0139 | 0.0309 |  |  |  |  |
| rs736282 | C | T | C | T | -0.01082 | 0.0017 | 2.07E-10 | 0.005696 | 0.0136 | 0.6759 |  |  |  |  |
| rs73874335 | C | T | C | T | 0.0199 | 0.00361 | 3.40E-08 | -0.0453 | 0.0293 | 0.1227 |  |  |  |  |
| rs743316 | T | C | T | C | 0.01185 | 0.00208 | 1.20E-08 | -0.0267 | 0.0164 | 0.103 |  |  |  |  |
| rs74545339 | A | G | A | G | -0.01626 | 0.00269 | 1.44E-09 | -0.0252 | 0.0213 | 0.2367 |  |  |  |  |
| rs74701752 | G | T | G | T | -0.01591 | 0.00285 | 2.38E-08 | 0.036104 | 0.0223 | 0.1049 |  |  |  |  |
| rs74944275 | C | T | C | T | -0.02739 | 0.00424 | 1.02E-10 | 0.028204 | 0.0346 | 0.4151 |  |  |  |  |
| rs74998289 | T | G | T | G | 0.01821 | 0.00213 | 1.31E-17 | -0.046 | 0.0169 | 0.006517 |  |  |  |  |
| rs7526112 | T | G | T | G | 0.01215 | 0.00177 | 6.10E-12 | -0.0071 | 0.0142 | 0.6147 |  |  |  |  |
| rs75708852 | A | C | A | C | -0.02792 | 0.00498 | 2.09E-08 | 0.032002 | 0.0359 | 0.3728 |  |  |  |  |
| rs75755471 | A | G | A | G | -0.01891 | 0.00344 | 3.79E-08 | 0.007998 | 0.0272 | 0.7681 |  |  |  |  |
| rs7575637 | A | G | A | G | 0.01136 | 0.00171 | 2.86E-11 | 0.017398 | 0.0136 | 0.2008 |  |  |  |  |
| rs7595950 | C | T | C | T | -0.00993 | 0.0017 | 4.99E-09 | 0.0127 | 0.0136 | 0.3475 |  |  |  |  |
| rs7597126 | C | T | C | T | 0.01009 | 0.00172 | 4.20E-09 | -0.0094 | 0.0139 | 0.4966 |  |  |  |  |
| rs7603132 | G | A | G | A | -0.01317 | 0.00215 | 9.17E-10 | 0.041604 | 0.0175 | 0.01774 |  |  |  |  |
| rs76076331 | C | T | C | T | -0.01873 | 0.00248 | 4.40E-14 | 0.087302 | 0.0198 | 1.07E-05 |  |  |  |  |
| rs7625428 | C | T | C | T | -0.00989 | 0.00174 | 1.26E-08 | -0.0111 | 0.014 | 0.4267 |  |  |  |  |
| rs77025239 | A | G | A | G | -0.01422 | 0.00234 | 1.33E-09 | 0.005196 | 0.0185 | 0.7806 |  |  |  |  |
| rs7737905 | T | G | T | G | -0.01285 | 0.00188 | 8.34E-12 | 0.038297 | 0.0152 | 0.01153 |  |  |  |  |
| rs77702819 | G | T | G | T | -0.01863 | 0.00298 | 3.92E-10 | 0.033402 | 0.0266 | 0.2092 |  |  |  |  |
| rs77835879 | A | G | A | G | 0.01601 | 0.00288 | 2.68E-08 | 0.005803 | 0.0257 | 0.8202 |  |  |  |  |
| rs7788620 | G | A | G | A | -0.01635 | 0.00206 | 1.84E-15 | 0.045196 | 0.0159 | 0.004419 |  |  |  |  |
| rs7796203 | G | A | G | A | 0.01074 | 0.00171 | 3.60E-10 | -0.0177 | 0.0138 | 0.1991 |  |  |  |  |
| rs7803932 | A | G | A | G | 0.0143 | 0.00226 | 2.44E-10 | -0.0268 | 0.0184 | 0.1457 |  |  |  |  |
| rs790647 | C | A | C | A | 0.01482 | 0.00202 | 2.17E-13 | -0.0379 | 0.0161 | 0.0184 |  |  |  |  |
| rs7924036 | T | G | T | G | 0.01501 | 0.0017 | 1.07E-18 | -0.0155 | 0.0135 | 0.2511 |  |  |  |  |
| rs79265434 | A | G | A | G | -0.02331 | 0.00262 | 6.08E-19 | -0.0479 | 0.0217 | 0.02749 |  |  |  |  |
| rs79269403 | G | A | G | A | -0.01447 | 0.00204 | 1.17E-12 | 0.015601 | 0.0167 | 0.3506 |  |  |  |  |
| rs7928017 | C | A | C | A | -0.00953 | 0.00172 | 2.83E-08 | 0.025205 | 0.014 | 0.07177 |  |  |  |  |
| rs79375112 | G | A | G | A | -0.01529 | 0.00257 | 2.63E-09 | 0.006099 | 0.021 | 0.7716 |  |  |  |  |
| rs7943853 | C | T | C | T | -0.01128 | 0.00201 | 2.06E-08 | 0 | 0.0161 | 0.9993 |  |  |  |  |
| rs795230 | T | C | T | C | 0.00952 | 0.00172 | 2.97E-08 | 0.006995 | 0.0135 | 0.6058 |  |  |  |  |
| rs79523955 | A | G | A | G | 0.01802 | 0.00283 | 1.87E-10 | -0.0301 | 0.0228 | 0.1854 |  |  |  |  |
| rs8008382 | T | C | T | C | -0.01208 | 0.00185 | 6.12E-11 | -0.0215 | 0.0147 | 0.1445 |  |  |  |  |
| rs80171383 | A | G | A | G | 0.0145 | 0.00241 | 1.83E-09 | -0.0548 | 0.02 | 0.006232 |  |  |  |  |
| rs8020034 | G | A | G | A | -0.01782 | 0.00223 | 1.17E-15 | 0.017004 | 0.0179 | 0.3421 |  |  |  |  |
| rs8052297 | T | G | T | G | -0.01029 | 0.00172 | 2.10E-09 | 0.011296 | 0.0137 | 0.4088 |  |  |  |  |
| rs818415 | T | G | T | G | -0.01235 | 0.00219 | 1.72E-08 | 0.014297 | 0.0176 | 0.4158 |  |  |  |  |
| rs837080 | C | T | C | T | 0.01092 | 0.0017 | 1.43E-10 | 0.006501 | 0.0135 | 0.6321 |  |  |  |  |
| rs892612 | C | A | C | A | 0.01464 | 0.00237 | 6.63E-10 | -0.0015 | 0.0188 | 0.9377 |  |  |  |  |
| rs9289300 | T | C | T | C | -0.01512 | 0.00234 | 1.10E-10 | 0.033502 | 0.0188 | 0.07473 |  |  |  |  |
| rs9320493 | G | A | G | A | -0.01394 | 0.0024 | 6.13E-09 | -0.0045 | 0.0197 | 0.818 |  |  |  |  |
| rs9342482 | T | G | T | G | 0.01264 | 0.00197 | 1.36E-10 | 0.018596 | 0.0156 | 0.231 |  |  |  |  |
| rs9349956 | A | C | A | C | -0.01881 | 0.00225 | 6.28E-17 | -0.0071 | 0.0188 | 0.7039 |  |  |  |  |
| rs9371881 | G | A | G | A | -0.01036 | 0.00178 | 5.76E-09 | 0.013298 | 0.0143 | 0.3499 |  |  |  |  |
| rs9372625 | A | G | A | G | 0.02383 | 0.00176 | 6.76E-42 | -0.0177 | 0.0142 | 0.2135 |  |  |  |  |
| rs9384679 | T | C | T | C | -0.00959 | 0.00176 | 4.88E-08 | -0.0162 | 0.0141 | 0.2481 |  |  |  |  |
| rs9386319 | G | A | G | A | 0.00991 | 0.00174 | 1.27E-08 | -0.0375 | 0.0138 | 0.006542 |  |  |  |  |
| rs9386787 | A | G | A | G | -0.00958 | 0.0017 | 1.82E-08 | 0.011405 | 0.0135 | 0.4 |  |  |  |  |
| rs9388490 | T | C | T | C | 0.00972 | 0.00171 | 1.43E-08 | -0.036 | 0.0136 | 0.008059 |  |  |  |  |
| rs939400 | G | T | G | T | 0.01031 | 0.00177 | 5.28E-09 | -0.0256 | 0.0139 | 0.06564 |  |  |  |  |
| rs9436866 | C | A | C | A | 0.01882 | 0.00289 | 7.45E-11 | 0.009404 | 0.0239 | 0.695 |  |  |  |  |
| rs9503598 | A | G | A | G | 0.01079 | 0.00171 | 3.12E-10 | 0.009098 | 0.0138 | 0.5118 |  |  |  |  |
| rs9513416 | G | A | G | A | 0.01316 | 0.00232 | 1.46E-08 | -0.0127 | 0.0188 | 0.4976 |  |  |  |  |
| rs9536961 | A | G | A | G | -0.01242 | 0.0018 | 4.96E-12 | 0.023003 | 0.0148 | 0.1194 |  |  |  |  |
| rs9556958 | C | T | C | T | 0.0108 | 0.0017 | 2.38E-10 | -0.005 | 0.0137 | 0.7129 |  |  |  |  |
| rs9557378 | A | G | A | G | -0.01094 | 0.00193 | 1.45E-08 | 0.013804 | 0.0151 | 0.3603 |  |  |  |  |
| rs9616906 | G | A | G | A | -0.01497 | 0.00172 | 2.92E-18 | 0.0242 | 0.0136 | 0.07445 |  |  |  |  |
| rs9704097 | A | C | A | C | -0.0103 | 0.00171 | 1.61E-09 | 0.010198 | 0.0135 | 0.4484 |  |  |  |  |
| rs9771228 | T | C | T | C | 0.01182 | 0.00178 | 3.07E-11 | -0.036 | 0.0142 | 0.01107 |  |  |  |  |
| rs9882532 | C | T | C | T | -0.01208 | 0.00177 | 8.17E-12 | 0.042396 | 0.0139 | 0.002354 |  |  |  |  |
| rs9914918 | G | A | G | A | -0.01155 | 0.00189 | 8.90E-10 | 0.027104 | 0.0151 | 0.07359 |  |  |  |  |
| rs9936270 | T | C | T | C | -0.0136 | 0.00198 | 6.43E-12 | 0.030704 | 0.0158 | 0.05264 |  |  |  |  |
| rs9964724 | C | T | C | T | -0.01978 | 0.00183 | 2.66E-27 | 0.034695 | 0.0144 | 0.01551 |  |  |  |  |
| rs997123 | C | T | C | T | 0.00966 | 0.0017 | 1.32E-08 | -0.0067 | 0.0136 | 0.62 |  |  |  |  |
| rs998887 | A | C | A | C | 0.01272 | 0.0017 | 7.42E-14 | -0.0346 | 0.0135 | 0.01034 |  |  |  |  |
| rs10120798 | A | G | A | G | -0.01028 | 0.00173 | 2.74E-09 | 0.048104 | 0.0136 | 0.000422 | *Removed from Steiger filtering | | | |
| rs10205801 | A | G | A | G | -0.01053 | 0.00171 | 7.17E-10 | 0.049304 | 0.0136 | 0.000277 |  |  |  |  |
| rs10215082 | A | G | A | G | -0.01303 | 0.00172 | 3.33E-14 | 0.047399 | 0.0138 | 0.000598 |  |  |  |  |
| rs10433551 | A | G | A | G | 0.01448 | 0.00265 | 4.73E-08 | -0.0605 | 0.0228 | 0.007843 |  |  |  |  |
| rs10460095 | G | A | G | A | 0.01066 | 0.00171 | 4.87E-10 | -0.0403 | 0.0137 | 0.003143 |  |  |  |  |
| rs1054442 | C | A | C | A | 0.01426 | 0.00177 | 8.42E-16 | -0.0496 | 0.0143 | 0.000517 |  |  |  |  |
| rs10752262 | C | T | C | T | -0.01072 | 0.00174 | 7.80E-10 | 0.041604 | 0.0145 | 0.004139 |  |  |  |  |
| rs11019128 | T | C | T | C | -0.01123 | 0.00175 | 1.43E-10 | 0.037103 | 0.0139 | 0.007651 |  |  |  |  |
| rs11210934 | G | A | G | A | -0.01275 | 0.00194 | 4.84E-11 | 0.082903 | 0.0153 | 6.26E-08 |  |  |  |  |
| rs11223560 | G | A | G | A | -0.00995 | 0.00173 | 9.68E-09 | 0.032802 | 0.014 | 0.01868 |  |  |  |  |
| rs1143770 | C | T | C | T | -0.01136 | 0.00172 | 4.31E-11 | 0.030799 | 0.0137 | 0.02452 |  |  |  |  |
| rs11663602 | A | C | A | C | -0.01213 | 0.0019 | 1.64E-10 | 0.046101 | 0.0152 | 0.002445 |  |  |  |  |
| rs1167827 | G | A | G | A | -0.00969 | 0.00173 | 2.31E-08 | 0.029398 | 0.014 | 0.03624 |  |  |  |  |
| rs11678980 | G | A | G | A | 0.01744 | 0.00172 | 4.29E-24 | -0.0465 | 0.0162 | 0.004183 |  |  |  |  |
| rs12273435 | G | A | G | A | 0.01152 | 0.0021 | 3.96E-08 | -0.0386 | 0.0183 | 0.03465 |  |  |  |  |
| rs12468040 | G | T | G | T | -0.01432 | 0.00175 | 2.46E-16 | 0.043795 | 0.0141 | 0.001872 |  |  |  |  |
| rs12643771 | C | T | C | T | -0.01518 | 0.00184 | 1.61E-16 | 0.047396 | 0.0156 | 0.002392 |  |  |  |  |
| rs12655753 | A | G | A | G | -0.02903 | 0.00509 | 1.18E-08 | 0.092096 | 0.0423 | 0.02954 |  |  |  |  |
| rs12778624 | T | G | T | G | 0.01099 | 0.00196 | 2.02E-08 | -0.0325 | 0.0152 | 0.03255 |  |  |  |  |
| rs1291818 | C | T | C | T | -0.01085 | 0.0017 | 1.78E-10 | 0.030903 | 0.0141 | 0.02873 |  |  |  |  |
| rs13029509 | A | G | A | G | -0.01049 | 0.0017 | 7.17E-10 | 0.034498 | 0.0135 | 0.01088 |  |  |  |  |
| rs1381247 | C | T | C | T | -0.01013 | 0.00182 | 2.46E-08 | 0.034302 | 0.0157 | 0.02897 |  |  |  |  |
| rs1475974 | C | T | C | T | 0.01269 | 0.0018 | 2.02E-12 | -0.0334 | 0.0145 | 0.02095 |  |  |  |  |
| rs152603 | G | A | G | A | 0.01019 | 0.00177 | 9.47E-09 | -0.0383 | 0.0144 | 0.007931 |  |  |  |  |
| rs1527878 | G | A | G | A | 0.01182 | 0.00199 | 3.08E-09 | -0.0405 | 0.0158 | 0.01032 |  |  |  |  |
| rs1569723 | A | C | A | C | 0.01168 | 0.00197 | 3.01E-09 | -0.0671 | 0.0156 | 1.71E-05 |  |  |  |  |
| rs16854920 | T | C | T | C | -0.01007 | 0.00181 | 2.51E-08 | 0.033299 | 0.0153 | 0.02924 |  |  |  |  |
| rs1758747 | A | G | A | G | 0.0101 | 0.00184 | 4.13E-08 | -0.0319 | 0.0149 | 0.03205 |  |  |  |  |
| rs1925587 | C | T | C | T | -0.01016 | 0.00171 | 3.03E-09 | 0.027104 | 0.0136 | 0.04583 |  |  |  |  |
| rs1947114 | G | A | G | A | 0.01071 | 0.00192 | 2.64E-08 | -0.043 | 0.0153 | 0.004817 |  |  |  |  |
| rs2141277 | A | G | A | G | 0.00952 | 0.0017 | 2.16E-08 | -0.03381 | 0.0135 | 0.01249 |  |  |  |  |
| rs2297600 | G | T | G | T | -0.01569 | 0.00223 | 2.16E-12 | 0.060196 | 0.0177 | 0.000678 |  |  |  |  |
| rs2365376 | C | A | C | A | -0.0107 | 0.00179 | 2.15E-09 | 0.029295 | 0.0144 | 0.04139 |  |  |  |  |
| rs2554835 | G | A | G | A | -0.00974 | 0.00175 | 2.69E-08 | -0.0292 | 0.0145 | 0.04402 |  |  |  |  |
| rs268120 | A | G | A | G | 0.01244 | 0.00196 | 2.13E-10 | -0.0343 | 0.0153 | 0.02487 |  |  |  |  |
| rs2787101 | C | T | C | T | -0.00968 | 0.00174 | 2.50E-08 | 0.038595 | 0.0138 | 0.005238 |  |  |  |  |
| rs281302 | A | G | A | G | -0.0111 | 0.00172 | 9.84E-11 | 0.030597 | 0.0139 | 0.02709 |  |  |  |  |
| rs2819336 | T | C | T | C | 0.01828 | 0.00177 | 5.46E-25 | -0.089 | 0.0141 | 2.83E-10 |  |  |  |  |
| rs2820314 | A | C | A | C | 0.011 | 0.0018 | 9.34E-10 | -0.0371 | 0.0143 | 0.009695 |  |  |  |  |
| rs28661002 | T | C | T | C | 0.01099 | 0.00198 | 2.89E-08 | 0.035502 | 0.0157 | 0.02399 |  |  |  |  |
| rs2971970 | T | G | T | G | -0.01654 | 0.00207 | 1.25E-15 | 0.046196 | 0.0165 | 0.005098 |  |  |  |  |
| rs34394051 | G | A | G | A | 0.01392 | 0.0024 | 6.20E-09 | -0.0437 | 0.0187 | 0.0191 |  |  |  |  |
| rs35417702 | C | T | C | T | 0.01445 | 0.0017 | 1.93E-17 | -0.0581 | 0.0135 | 1.65E-05 |  |  |  |  |
| rs35919256 | C | A | C | A | -0.01032 | 0.00177 | 5.56E-09 | 0.044903 | 0.0141 | 0.001447 |  |  |  |  |
| rs363096 | C | T | C | T | 0.01363 | 0.00172 | 2.04E-15 | -0.0356 | 0.0135 | 0.008441 |  |  |  |  |
| rs3781339 | T | C | T | C | -0.01247 | 0.00219 | 1.30E-08 | 0.034305 | 0.0173 | 0.04671 |  |  |  |  |
| rs3796348 | G | A | G | A | -0.01032 | 0.00175 | 3.66E-09 | 0.040801 | 0.0145 | 0.004865 |  |  |  |  |
| rs3809634 | A | G | A | G | -0.01058 | 0.00185 | 1.09E-08 | 0.031499 | 0.0146 | 0.03046 |  |  |  |  |
| rs4073894 | A | G | A | G | 0.01524 | 0.00211 | 5.40E-13 | -0.0435 | 0.0172 | 0.01126 |  |  |  |  |
| rs4352658 | T | C | T | C | -0.0212 | 0.00308 | 5.55E-12 | 0.055898 | 0.0255 | 0.02864 |  |  |  |  |
| rs4442732 | G | A | G | A | -0.01063 | 0.00176 | 1.49E-09 | -0.0474 | 0.0138 | 0.000592 |  |  |  |  |
| rs4641552 | C | A | C | A | -0.01926 | 0.00336 | 1.02E-08 | 0.069404 | 0.0276 | 0.01204 |  |  |  |  |
| rs4726070 | G | A | G | A | -0.01251 | 0.00174 | 5.95E-13 | 0.039698 | 0.0136 | 0.003669 |  |  |  |  |
| rs4760687 | G | A | G | A | 0.01039 | 0.00183 | 1.30E-08 | -0.0395 | 0.0148 | 0.007393 |  |  |  |  |
| rs4976445 | T | C | T | C | 0.012 | 0.00197 | 1.16E-09 | -0.0309 | 0.0156 | 0.04703 |  |  |  |  |
| rs60096640 | G | A | G | A | -0.01574 | 0.00273 | 7.82E-09 | 0.041395 | 0.0208 | 0.04659 |  |  |  |  |
| rs62174974 | G | A | G | A | 0.01191 | 0.00215 | 2.83E-08 | -0.0524 | 0.017 | 0.002071 |  |  |  |  |
| rs62444881 | C | T | C | T | -0.01815 | 0.00217 | 5.79E-17 | 0.062301 | 0.0172 | 0.000291 |  |  |  |  |
| rs6457996 | C | T | C | T | -0.01014 | 0.0017 | 2.43E-09 | 0.036996 | 0.0137 | 0.007008 |  |  |  |  |
| rs6557171 | C | T | C | T | 0.01567 | 0.00181 | 4.15E-18 | -0.0483 | 0.0146 | 0.000911 |  |  |  |  |
| rs66568921 | G | T | G | T | 0.01565 | 0.00182 | 7.49E-18 | -0.0408 | 0.0141 | 0.003921 |  |  |  |  |
| rs6736898 | A | G | A | G | 0.01032 | 0.00178 | 6.09E-09 | -0.0472 | 0.0141 | 0.000832 |  |  |  |  |
| rs67890737 | C | A | C | A | 0.01141 | 0.00179 | 2.01E-10 | -0.037 | 0.0141 | 0.008449 |  |  |  |  |
| rs6821231 | C | T | C | T | 0.01217 | 0.00197 | 6.06E-10 | -0.0343 | 0.0158 | 0.03013 |  |  |  |  |
| rs6871635 | A | G | A | G | -0.00946 | 0.00173 | 4.63E-08 | 0.032099 | 0.0141 | 0.02269 |  |  |  |  |
| rs7117878 | A | C | A | C | -0.01036 | 0.00182 | 1.18E-08 | 0.043902 | 0.0144 | 0.002307 |  |  |  |  |
| rs7139165 | C | A | C | A | -0.01136 | 0.00195 | 5.29E-09 | 0.042 | 0.0146 | 0.003875 |  |  |  |  |
| rs7257460 | T | C | T | C | 0.01145 | 0.00189 | 1.25E-09 | -0.0515 | 0.0147 | 0.000467 |  |  |  |  |
| rs730384 | G | A | G | A | -0.01016 | 0.00171 | 3.01E-09 | 0.030098 | 0.0138 | 0.02863 |  |  |  |  |
| rs7332724 | T | C | T | C | -0.01149 | 0.00189 | 1.26E-09 | 0.036197 | 0.0149 | 0.01511 |  |  |  |  |
| rs7603132 | G | A | G | A | -0.01317 | 0.00215 | 9.17E-10 | 0.041604 | 0.0175 | 0.01774 |  |  |  |  |
| rs76076331 | C | T | C | T | -0.01873 | 0.00248 | 4.40E-14 | 0.087302 | 0.0198 | 1.07E-05 |  |  |  |  |
| rs7737905 | T | G | T | G | -0.01285 | 0.00188 | 8.34E-12 | 0.038297 | 0.0152 | 0.01153 |  |  |  |  |
| rs7788620 | G | A | G | A | -0.01635 | 0.00206 | 1.84E-15 | 0.045196 | 0.0159 | 0.004419 |  |  |  |  |
| rs7928017 | C | A | C | A | -0.00953 | 0.00172 | 2.83E-08 | 0.025205 | 0.014 | 0.07177 |  |  |  |  |
| rs80171383 | A | G | A | G | 0.0145 | 0.00241 | 1.83E-09 | -0.0548 | 0.02 | 0.006232 |  |  |  |  |
| rs9386319 | G | A | G | A | 0.00991 | 0.00174 | 1.27E-08 | -0.0375 | 0.0138 | 0.006542 |  |  |  |  |
| rs9388490 | T | C | T | C | 0.00972 | 0.00171 | 1.43E-08 | -0.036 | 0.0136 | 0.008059 |  |  |  |  |
| rs9771228 | T | C | T | C | 0.01182 | 0.00178 | 3.07E-11 | -0.036 | 0.0142 | 0.01107 |  |  |  |  |
| rs9882532 | C | T | C | T | -0.01208 | 0.00177 | 8.17E-12 | 0.042396 | 0.0139 | 0.002354 |  |  |  |  |
| rs998887 | A | C | A | C | 0.01272 | 0.0017 | 7.42E-14 | -0.0346 | 0.0135 | 0.01034 |  |  |  |  |
| rs10266047 | G | C | G | C | 0.01132 | 0.00171 | 3.27E-11 | -0.0095 | 0.0135 | 0.4814 | *Removed during harmonisation | | | |
| rs10417097 | G | C | G | C | 0.01014 | 0.00174 | 5.17E-09 | -0.0371 | 0.0142 | 0.008927 |  |  |  |  |
| rs10772644 | G | C | G | C | -0.01614 | 0.00267 | 1.50E-09 | -0.01 | 0.0209 | 0.6332 |  |  |  |  |
| rs10773002 | T | A | T | A | -0.02191 | 0.00197 | 8.68E-29 | 0.030098 | 0.0159 | 0.05786 |  |  |  |  |
| rs10862376 | T | A | T | A | -0.01616 | 0.00239 | 1.40E-11 | 0.0432 | 0.0193 | 0.02502 |  |  |  |  |
| rs10875121 | G | C | G | C | -0.01834 | 0.00226 | 5.53E-16 | 0.031697 | 0.0177 | 0.07429 |  |  |  |  |
| rs10963297 | G | C | G | C | 0.01904 | 0.00198 | 7.36E-22 | 0.001101 | 0.0161 | 0.9461 |  |  |  |  |
| rs11082975 | G | C | G | C | 0.01309 | 0.00172 | 2.52E-14 | -0.0336 | 0.0135 | 0.01308 |  |  |  |  |
| rs112806496 | G | C | G | C | 0.0187 | 0.00305 | 8.28E-10 | -0.0086 | 0.0257 | 0.737 |  |  |  |  |
| rs112969166 | G | C | G | C | -0.01008 | 0.00171 | 3.81E-09 | 0.0044 | 0.0135 | 0.7443 |  |  |  |  |
| rs115000530 | T | A | T | A | 0.02892 | 0.00381 | 3.30E-14 | 0.019101 | 0.0315 | 0.5447 |  |  |  |  |
| rs11609711 | C | G | C | G | 0.01371 | 0.00241 | 1.35E-08 | 0.002996 | 0.0198 | 0.8791 |  |  |  |  |
| rs11708375 | G | C | G | C | 0.01376 | 0.00235 | 4.58E-09 | -0.016 | 0.0199 | 0.421 |  |  |  |  |
| rs117799466 | G | C | G | C | -0.01173 | 0.00198 | 2.91E-09 | -0.0285 | 0.017 | 0.09424 |  |  |  |  |
| rs12118513 | T | A | T | A | 0.01215 | 0.0021 | 7.47E-09 | -0.0214 | 0.0177 | 0.2265 |  |  |  |  |
| rs12332731 | A | T | A | T | 0.01374 | 0.00218 | 3.12E-10 | -0.0507 | 0.0177 | 0.004252 |  |  |  |  |
| rs1245829 | A | T | A | T | -0.01083 | 0.00173 | 3.51E-10 | 0.025502 | 0.0141 | 0.07102 |  |  |  |  |
| rs12764593 | C | G | C | G | 0.02293 | 0.00358 | 1.53E-10 | -0.0377 | 0.0306 | 0.2183 |  |  |  |  |
| rs13130765 | C | G | C | G | -0.01014 | 0.00173 | 4.69E-09 | 0.008295 | 0.016 | 0.6039 |  |  |  |  |
| rs1408284 | C | G | C | G | -0.01388 | 0.0025 | 2.74E-08 | 0.001699 | 0.0207 | 0.9328 |  |  |  |  |
| rs1455350 | T | A | T | A | 0.01614 | 0.0017 | 2.61E-21 | -0.0218 | 0.0136 | 0.1086 |  |  |  |  |
| rs1592757 | C | G | C | G | -0.01045 | 0.0018 | 5.89E-09 | 0.075098 | 0.0141 | 1.08E-07 |  |  |  |  |
| rs1689510 | G | C | G | C | -0.01761 | 0.0018 | 1.40E-22 | 0.026601 | 0.0145 | 0.06714 |  |  |  |  |
| rs17428076 | C | G | C | G | 0.01216 | 0.00198 | 8.90E-10 | -0.0182 | 0.0156 | 0.2435 |  |  |  |  |
| rs175325 | A | T | A | T | -0.01179 | 0.00174 | 1.11E-11 | -0.0105 | 0.0138 | 0.4458 |  |  |  |  |
| rs17568389 | A | T | A | T | 0.01201 | 0.0017 | 1.53E-12 | -0.009 | 0.0135 | 0.5033 |  |  |  |  |
| rs1960603 | C | G | C | G | 0.01096 | 0.00198 | 3.07E-08 | -0.0085 | 0.0159 | 0.5943 |  |  |  |  |
| rs2414072 | A | T | A | T | -0.01005 | 0.00171 | 4.35E-09 | 0.016198 | 0.0136 | 0.2329 |  |  |  |  |
| rs2478208 | C | G | C | G | -0.0106 | 0.0017 | 4.82E-10 | 0.009098 | 0.0136 | 0.5042 |  |  |  |  |
| rs2545798 | T | A | T | A | 0.01346 | 0.00171 | 3.11E-15 | 0.0008 | 0.0144 | 0.9541 |  |  |  |  |
| rs28373063 | G | C | G | C | -0.01389 | 0.00229 | 1.23E-09 | 0.043503 | 0.019 | 0.02188 |  |  |  |  |
| rs2923431 | C | G | C | G | 0.0114 | 0.00176 | 9.84E-11 | -0.0145 | 0.0139 | 0.2979 |  |  |  |  |
| rs34410 | G | C | G | C | 0.00931 | 0.0017 | 4.70E-08 | -0.0479 | 0.0136 | 0.00042 |  |  |  |  |
| rs35518360 | A | T | A | T | 0.01875 | 0.00327 | 9.79E-09 | -0.0029 | 0.0292 | 0.9205 |  |  |  |  |
| rs3747631 | G | C | G | C | -0.02207 | 0.00208 | 2.97E-26 | 0.0382 | 0.0165 | 0.02102 |  |  |  |  |
| rs3768480 | G | C | G | C | -0.01038 | 0.00172 | 1.74E-09 | 0.011 | 0.0138 | 0.4236 |  |  |  |  |
| rs3800546 | G | C | G | C | -0.01183 | 0.00194 | 9.73E-10 | -0.0088 | 0.0155 | 0.5715 |  |  |  |  |
| rs401687 | G | C | G | C | -0.01144 | 0.0017 | 1.86E-11 | 0.020305 | 0.0135 | 0.1318 |  |  |  |  |
| rs406413 | A | T | A | T | 0.01695 | 0.00209 | 4.84E-16 | -0.0029 | 0.0162 | 0.8578 |  |  |  |  |
| rs4728278 | C | G | C | G | 0.01066 | 0.00184 | 6.38E-09 | -0.0215 | 0.0149 | 0.1481 |  |  |  |  |
| rs4733264 | C | G | C | G | -0.00954 | 0.00174 | 4.53E-08 | -0.0044 | 0.0137 | 0.7506 |  |  |  |  |
| rs4757957 | C | G | C | G | 0.0141 | 0.00184 | 1.81E-14 | -0.0364 | 0.0146 | 0.01275 |  |  |  |  |
| rs4766424 | G | C | G | C | -0.0141 | 0.00258 | 4.43E-08 | 0.003105 | 0.0206 | 0.8804 |  |  |  |  |
| rs4877516 | T | A | T | A | 0.01163 | 0.00171 | 1.02E-11 | -0.0084 | 0.0136 | 0.5389 |  |  |  |  |
| rs4984682 | G | C | G | C | 0.01202 | 0.00203 | 3.13E-09 | -0.0504 | 0.0175 | 0.003906 |  |  |  |  |
| rs55736314 | C | G | C | G | -0.01431 | 0.00174 | 1.63E-16 | 0.041698 | 0.014 | 0.002897 |  |  |  |  |
| rs55771711 | C | G | C | G | 0.01555 | 0.00199 | 5.41E-15 | -0.0059 | 0.0163 | 0.7155 |  |  |  |  |
| rs59480703 | G | C | G | C | 0.01237 | 0.00215 | 8.32E-09 | -0.0289 | 0.0169 | 0.08745 |  |  |  |  |
| rs59953820 | A | T | A | T | -0.01595 | 0.00289 | 3.50E-08 | -0.0223 | 0.0236 | 0.3437 |  |  |  |  |
| rs60483752 | G | C | G | C | -0.01078 | 0.00172 | 3.89E-10 | 0.016201 | 0.014 | 0.2455 |  |  |  |  |
| rs62247449 | C | G | C | G | 0.01137 | 0.00171 | 3.31E-11 | 0.014997 | 0.0138 | 0.2768 |  |  |  |  |
| rs663234 | G | C | G | C | -0.01005 | 0.00174 | 7.39E-09 | 0.010505 | 0.0139 | 0.4512 |  |  |  |  |
| rs6678474 | T | A | T | A | -0.02927 | 0.00513 | 1.13E-08 | 0.058297 | 0.043 | 0.175 |  |  |  |  |
| rs6867851 | C | G | C | G | -0.012 | 0.00173 | 3.97E-12 | 0.018998 | 0.0142 | 0.181 |  |  |  |  |
| rs7016302 | G | C | G | C | 0.01243 | 0.00228 | 4.98E-08 | 0.001301 | 0.0182 | 0.9431 |  |  |  |  |
| rs7278859 | A | T | A | T | -0.01013 | 0.00185 | 4.15E-08 | 0.036399 | 0.0146 | 0.01266 |  |  |  |  |
| rs73496688 | T | A | T | A | -0.01429 | 0.00238 | 2.04E-09 | 0.007901 | 0.0193 | 0.6815 |  |  |  |  |
| rs76878669 | C | G | C | G | 0.01399 | 0.00205 | 8.67E-12 | 0.010999 | 0.0158 | 0.4865 |  |  |  |  |
| rs7833201 | C | G | C | G | -0.01532 | 0.00262 | 5.05E-09 | 0.016601 | 0.0216 | 0.4434 |  |  |  |  |
| rs7920624 | T | A | T | A | -0.01181 | 0.0017 | 3.97E-12 | 0.014099 | 0.0135 | 0.2941 |  |  |  |  |
| rs7928622 | T | A | T | A | 0.01011 | 0.00181 | 2.52E-08 | 0.006602 | 0.0149 | 0.6547 |  |  |  |  |
| rs925161 | G | C | G | C | 0.00941 | 0.0017 | 3.26E-08 | -0.0122 | 0.0135 | 0.367 |  |  |  |  |
| rs9529119 | C | G | C | G | 0.01295 | 0.00204 | 2.13E-10 | -0.0182 | 0.0167 | 0.2745 |  |  |  |  |
| rs969512 | T | A | T | A | 0.01249 | 0.00179 | 3.22E-12 | -0.0209 | 0.0143 | 0.142 |  |  |  |  |
| rs9886703 | A | T | A | T | -0.01315 | 0.00227 | 7.14E-09 | 0.029598 | 0.0187 | 0.1134 |  |  |  |  |
| rs9938678 | A | T | A | T | -0.01355 | 0.00205 | 4.12E-11 | 0.021898 | 0.0169 | 0.1941 |  |  |  |  |

| **Table S2c.** Harmonised instruments used in the MR analyses investigating the causal effect of genetic liability to higher educational attainment on ASD | | | | | | | | | | | | | | |
| --- | --- | --- | --- | --- | --- | --- | --- | --- | --- | --- | --- | --- | --- | --- |
| **SNP** | **A1.EA** | **A2.EA** | **A1.ASD** | **A2.ASD** | **Beta.EA** | **SE.EA** | **P.EA** | **logOR.ASD** | **SE.ASD** | **P.ASD** |  |  |  |  |
| rs10060023 | C | T | C | T | -0.01178 | 0.00182 | 9.59E-11 | -0.0026 | 0.015 | 0.8641 |  |  |  |  |
| rs1007731 | C | A | C | A | 0.0151 | 0.00269 | 2.07E-08 | 0.024805 | 0.0242 | 0.3053 |  |  |  |  |
| rs1008078 | T | C | T | C | -0.01738 | 0.00173 | 1.20E-23 | -0.0336 | 0.0141 | 0.017 |  |  |  |  |
| rs10098073 | A | C | A | C | -0.01185 | 0.00171 | 3.71E-12 | 0.004102 | 0.0139 | 0.7686 |  |  |  |  |
| rs10120798 | A | G | A | G | -0.01028 | 0.00173 | 2.74E-09 | 0.002597 | 0.014 | 0.851 |  |  |  |  |
| rs10145520 | T | G | T | G | -0.01191 | 0.00213 | 2.17E-08 | 0.002696 | 0.0175 | 0.8763 |  |  |  |  |
| rs10189857 | G | A | G | A | -0.01725 | 0.00171 | 6.70E-24 | -0.0043 | 0.0139 | 0.7584 |  |  |  |  |
| rs10191758 | A | G | A | G | -0.01631 | 0.00175 | 9.60E-21 | -0.0226 | 0.0142 | 0.1098 |  |  |  |  |
| rs1043209 | A | G | A | G | 0.01364 | 0.00174 | 4.22E-15 | -0.0054 | 0.0142 | 0.7013 |  |  |  |  |
| rs10433551 | A | G | A | G | 0.01448 | 0.00265 | 4.73E-08 | 0.002297 | 0.0219 | 0.9165 |  |  |  |  |
| rs10456918 | A | C | A | C | -0.01485 | 0.00224 | 3.67E-11 | 0.006995 | 0.0181 | 0.6963 |  |  |  |  |
| rs10460095 | G | A | G | A | 0.01066 | 0.00171 | 4.87E-10 | -0.0002 | 0.014 | 0.9912 |  |  |  |  |
| rs10496091 | G | A | G | A | 0.01403 | 0.00188 | 7.47E-14 | -0.0256 | 0.0154 | 0.09636 |  |  |  |  |
| rs1051474 | T | C | T | C | -0.01301 | 0.00188 | 4.86E-12 | -0.003 | 0.0154 | 0.8438 |  |  |  |  |
| rs10519504 | T | G | T | G | 0.01381 | 0.00235 | 3.92E-09 | -0.0026 | 0.0192 | 0.893 |  |  |  |  |
| rs1054442 | C | A | C | A | 0.01426 | 0.00177 | 8.42E-16 | 0.0008 | 0.0144 | 0.9559 |  |  |  |  |
| rs10616 | T | C | T | C | 0.0103 | 0.00185 | 2.74E-08 | -0.0067 | 0.015 | 0.657 |  |  |  |  |
| rs1061801 | A | G | A | G | -0.01354 | 0.0022 | 8.34E-10 | -0.0013 | 0.0182 | 0.9431 |  |  |  |  |
| rs10752262 | C | T | C | T | -0.01072 | 0.00174 | 7.80E-10 | -0.0017 | 0.0144 | 0.9035 |  |  |  |  |
| rs10765775 | A | G | A | G | 0.01488 | 0.00176 | 2.62E-17 | 0.019705 | 0.0141 | 0.1633 |  |  |  |  |
| rs10795831 | T | G | T | G | -0.01223 | 0.00208 | 3.99E-09 | -0.0044 | 0.0166 | 0.7927 |  |  |  |  |
| rs10797055 | G | A | G | A | 0.00986 | 0.00172 | 1.04E-08 | 0.0229 | 0.0139 | 0.09986 |  |  |  |  |
| rs10810099 | A | G | A | G | -0.015 | 0.0019 | 3.31E-15 | -0.0165 | 0.0159 | 0.2993 |  |  |  |  |
| rs10853455 | T | G | T | G | -0.01163 | 0.00211 | 3.61E-08 | -0.0033 | 0.0169 | 0.8464 |  |  |  |  |
| rs10856785 | T | C | T | C | -0.01132 | 0.00192 | 3.83E-09 | -0.0022 | 0.0156 | 0.8895 |  |  |  |  |
| rs10879676 | T | C | T | C | -0.00982 | 0.00173 | 1.26E-08 | 0.024 | 0.0139 | 0.08393 |  |  |  |  |
| rs10887801 | G | T | G | T | -0.01087 | 0.00171 | 2.27E-10 | -0.0011 | 0.0139 | 0.9379 |  |  |  |  |
| rs10906186 | C | T | C | T | -0.00991 | 0.00172 | 7.91E-09 | 0.011 | 0.0139 | 0.4303 |  |  |  |  |
| rs10940921 | G | T | G | T | -0.01089 | 0.00177 | 7.00E-10 | -0.0028 | 0.0139 | 0.8396 |  |  |  |  |
| rs10984445 | G | A | G | A | -0.01158 | 0.00171 | 1.17E-11 | 0.008002 | 0.0139 | 0.5628 |  |  |  |  |
| rs10994777 | G | A | G | A | -0.0146 | 0.00232 | 3.36E-10 | -0.0106 | 0.0186 | 0.5699 |  |  |  |  |
| rs11019128 | T | C | T | C | -0.01123 | 0.00175 | 1.43E-10 | -0.0055 | 0.0143 | 0.7002 |  |  |  |  |
| rs11023749 | A | G | A | G | 0.01132 | 0.0018 | 2.96E-10 | 0.013202 | 0.0146 | 0.3657 |  |  |  |  |
| rs1105307 | A | G | A | G | -0.01173 | 0.00195 | 1.67E-09 | 0.018596 | 0.0157 | 0.2383 |  |  |  |  |
| rs1106090 | A | G | A | G | 0.01173 | 0.00175 | 2.09E-11 | 0.007403 | 0.0143 | 0.6032 |  |  |  |  |
| rs11123818 | G | A | G | A | -0.02081 | 0.00175 | 1.72E-32 | -0.0125 | 0.0142 | 0.3783 |  |  |  |  |
| rs111821073 | C | T | C | T | -0.01385 | 0.00237 | 4.85E-09 | 0.014697 | 0.0189 | 0.4371 |  |  |  |  |
| rs111852224 | T | C | T | C | 0.01663 | 0.00262 | 2.34E-10 | 0.026798 | 0.0218 | 0.2176 |  |  |  |  |
| rs11210934 | G | A | G | A | -0.01275 | 0.00194 | 4.84E-11 | 0.031305 | 0.0159 | 0.04833 |  |  |  |  |
| rs11223560 | G | A | G | A | -0.00995 | 0.00173 | 9.68E-09 | 0.021898 | 0.0142 | 0.1237 |  |  |  |  |
| rs11259919 | A | G | A | G | -0.01093 | 0.0019 | 9.31E-09 | 0.021204 | 0.0154 | 0.1683 |  |  |  |  |
| rs112687095 | G | A | G | A | -0.01325 | 0.00238 | 2.42E-08 | 0.023996 | 0.021 | 0.2527 |  |  |  |  |
| rs113182709 | G | A | G | A | -0.03225 | 0.00567 | 1.29E-08 | 0.0242 | 0.0533 | 0.6497 |  |  |  |  |
| rs1143770 | C | T | C | T | -0.01136 | 0.00172 | 4.31E-11 | 0.009303 | 0.014 | 0.5078 |  |  |  |  |
| rs115438240 | G | T | G | T | 0.02162 | 0.00378 | 1.08E-08 | 0.028297 | 0.0307 | 0.3558 |  |  |  |  |
| rs11601122 | G | A | G | A | -0.01947 | 0.0023 | 2.24E-17 | -0.0119 | 0.0182 | 0.5137 |  |  |  |  |
| rs11620355 | G | A | G | A | -0.01756 | 0.003 | 4.77E-09 | -0.0021 | 0.026 | 0.9344 |  |  |  |  |
| rs11627087 | A | G | A | G | 0.01788 | 0.00325 | 3.71E-08 | 0.045604 | 0.0246 | 0.06383 |  |  |  |  |
| rs11635092 | A | G | A | G | -0.01231 | 0.00177 | 3.89E-12 | -0.0251 | 0.0156 | 0.1071 |  |  |  |  |
| rs11646221 | T | G | T | G | 0.01144 | 0.00172 | 3.01E-11 | -0.0029 | 0.0141 | 0.836 |  |  |  |  |
| rs11655029 | T | C | T | C | -0.0109 | 0.00183 | 2.84E-09 | -0.02481 | 0.0149 | 0.09551 |  |  |  |  |
| rs11657342 | A | G | A | G | 0.01404 | 0.00191 | 1.94E-13 | -0.0002 | 0.0153 | 0.9872 |  |  |  |  |
| rs1167827 | G | A | G | A | -0.00969 | 0.00173 | 2.31E-08 | 0.0168 | 0.0141 | 0.2332 |  |  |  |  |
| rs11678980 | G | A | G | A | 0.01744 | 0.00172 | 4.29E-24 | 0.008103 | 0.0147 | 0.5785 |  |  |  |  |
| rs11681861 | G | T | G | T | -0.01435 | 0.00259 | 2.88E-08 | -1.00E-04 | 0.0217 | 0.9958 |  |  |  |  |
| rs11694904 | T | C | T | C | 0.01215 | 0.00185 | 4.78E-11 | 0.025005 | 0.0153 | 0.1026 |  |  |  |  |
| rs11703948 | A | G | A | G | -0.01737 | 0.00285 | 1.06E-09 | -0.0128 | 0.0234 | 0.584 |  |  |  |  |
| rs11725086 | T | C | T | C | 0.00973 | 0.00174 | 2.40E-08 | 0.006797 | 0.014 | 0.6286 |  |  |  |  |
| rs11732657 | A | G | A | G | -0.01274 | 0.00197 | 9.54E-11 | -0.0013 | 0.016 | 0.9375 |  |  |  |  |
| rs118093058 | T | G | T | G | 0.01525 | 0.00257 | 2.87E-09 | -0.0004 | 0.0211 | 0.9864 |  |  |  |  |
| rs11871429 | A | G | A | G | 0.01425 | 0.00202 | 1.92E-12 | 0 | 0.0163 | 0.998 |  |  |  |  |
| rs12151248 | C | T | C | T | 0.01573 | 0.00271 | 6.75E-09 | 0.019295 | 0.0219 | 0.379 |  |  |  |  |
| rs12170452 | G | A | G | A | -0.01153 | 0.00171 | 1.40E-11 | -0.0149 | 0.0141 | 0.2909 |  |  |  |  |
| rs12273435 | G | A | G | A | 0.01152 | 0.0021 | 3.96E-08 | -0.0162 | 0.0185 | 0.381 |  |  |  |  |
| rs12290350 | T | C | T | C | 0.013 | 0.00209 | 4.70E-10 | -0.0149 | 0.017 | 0.3816 |  |  |  |  |
| rs12364080 | T | C | T | C | -0.01389 | 0.0025 | 2.89E-08 | -0.0074 | 0.021 | 0.7222 |  |  |  |  |
| rs12375949 | T | C | T | C | -0.01447 | 0.00172 | 3.31E-17 | 0.025005 | 0.014 | 0.07417 |  |  |  |  |
| rs12468040 | G | T | G | T | -0.01432 | 0.00175 | 2.46E-16 | -0.0194 | 0.0144 | 0.178 |  |  |  |  |
| rs12503522 | C | T | C | T | 0.01125 | 0.00188 | 2.24E-09 | 0.0127 | 0.0153 | 0.404 |  |  |  |  |
| rs12515541 | G | T | G | T | 0.01135 | 0.00174 | 6.95E-11 | 0.020703 | 0.0144 | 0.1498 |  |  |  |  |
| rs12574281 | A | C | A | C | -0.01077 | 0.00176 | 8.85E-10 | -0.0163 | 0.0145 | 0.2604 |  |  |  |  |
| rs12602286 | T | G | T | G | 0.01701 | 0.00255 | 2.37E-11 | 0.017103 | 0.0208 | 0.411 |  |  |  |  |
| rs12643771 | C | T | C | T | -0.01518 | 0.00184 | 1.61E-16 | 0.007599 | 0.0155 | 0.6228 |  |  |  |  |
| rs12646216 | T | C | T | C | 0.01062 | 0.00174 | 1.10E-09 | -0.0128 | 0.0143 | 0.3721 |  |  |  |  |
| rs12646297 | G | A | G | A | 0.0111 | 0.00187 | 2.76E-09 | 0.013501 | 0.0153 | 0.3768 |  |  |  |  |
| rs12655753 | A | G | A | G | -0.02903 | 0.00509 | 1.18E-08 | 0.037402 | 0.0433 | 0.388 |  |  |  |  |
| rs12682775 | T | C | T | C | -0.01187 | 0.00204 | 5.99E-09 | 0.0189 | 0.0169 | 0.2631 |  |  |  |  |
| rs12694681 | G | T | G | T | -0.01123 | 0.00183 | 9.06E-10 | 0.010798 | 0.015 | 0.4702 |  |  |  |  |
| rs12712269 | C | T | C | T | 0.01063 | 0.00174 | 9.51E-10 | -0.0066 | 0.0142 | 0.6413 |  |  |  |  |
| rs12716848 | A | G | A | G | -0.00969 | 0.00174 | 2.43E-08 | 0.005803 | 0.0142 | 0.6843 |  |  |  |  |
| rs12724430 | T | G | T | G | 0.00979 | 0.0017 | 8.32E-09 | 0.013597 | 0.0139 | 0.3262 |  |  |  |  |
| rs12778624 | T | G | T | G | 0.01099 | 0.00196 | 2.02E-08 | 0.004997 | 0.0156 | 0.7487 |  |  |  |  |
| rs1291818 | C | T | C | T | -0.01085 | 0.0017 | 1.78E-10 | -0.0028 | 0.0143 | 0.845 |  |  |  |  |
| rs12926704 | A | G | A | G | -0.01737 | 0.00269 | 1.03E-10 | 0.008999 | 0.0213 | 0.6711 |  |  |  |  |
| rs13010288 | G | T | G | T | -0.01953 | 0.00252 | 1.04E-14 | 0.012599 | 0.0201 | 0.5293 |  |  |  |  |
| rs13010566 | A | C | A | C | -0.0106 | 0.0017 | 4.59E-10 | 0.0005 | 0.0138 | 0.9691 |  |  |  |  |
| rs13029509 | A | G | A | G | -0.01049 | 0.0017 | 7.17E-10 | 0.020303 | 0.0139 | 0.1457 |  |  |  |  |
| rs13035874 | A | G | A | G | -0.01114 | 0.00178 | 4.34E-10 | 0.015696 | 0.0147 | 0.2852 |  |  |  |  |
| rs13090388 | C | T | C | T | -0.02852 | 0.00184 | 4.29E-54 | -0.0283 | 0.0151 | 0.06069 |  |  |  |  |
| rs13091704 | A | C | A | C | -0.0108 | 0.00197 | 4.11E-08 | -0.0043 | 0.0161 | 0.7907 |  |  |  |  |
| rs13141210 | C | T | C | T | -0.01361 | 0.00172 | 2.26E-15 | -0.0077 | 0.0142 | 0.5886 |  |  |  |  |
| rs13145650 | C | T | C | T | 0.01918 | 0.00306 | 3.80E-10 | 0.042104 | 0.0248 | 0.08983 |  |  |  |  |
| rs13147223 | A | G | A | G | 0.01087 | 0.00176 | 7.09E-10 | 0.007403 | 0.0155 | 0.6332 |  |  |  |  |
| rs1334297 | A | G | A | G | 0.02449 | 0.00192 | 3.06E-37 | -0.0243 | 0.0154 | 0.1141 |  |  |  |  |
| rs1335482 | C | T | C | T | -0.0096 | 0.0017 | 1.63E-08 | -1.00E-04 | 0.014 | 0.9961 |  |  |  |  |
| rs13422673 | C | T | C | T | 0.01201 | 0.0017 | 1.74E-12 | 0.016404 | 0.0139 | 0.2388 |  |  |  |  |
| rs1391438 | T | C | T | C | 0.0167 | 0.00183 | 5.79E-20 | 0.0003 | 0.0148 | 0.9865 |  |  |  |  |
| rs139244147 | G | A | G | A | 0.02095 | 0.00365 | 9.90E-09 | -0.02 | 0.0307 | 0.5141 |  |  |  |  |
| rs139612798 | C | T | C | T | 0.02918 | 0.00511 | 1.11E-08 | 0.022696 | 0.0485 | 0.6392 |  |  |  |  |
| rs1405876 | G | T | G | T | -0.01038 | 0.00177 | 4.40E-09 | -0.0069 | 0.0144 | 0.6327 |  |  |  |  |
| rs143386970 | T | C | T | C | 0.016 | 0.00286 | 2.20E-08 | 0.004997 | 0.0247 | 0.8387 |  |  |  |  |
| rs145590108 | G | T | G | T | -0.02232 | 0.00367 | 1.14E-09 | -0.0189 | 0.027 | 0.4838 |  |  |  |  |
| rs1475974 | C | T | C | T | 0.01269 | 0.0018 | 2.02E-12 | -0.0006 | 0.0147 | 0.9652 |  |  |  |  |
| rs1527878 | G | A | G | A | 0.01182 | 0.00199 | 3.08E-09 | -0.0253 | 0.0162 | 0.1176 |  |  |  |  |
| rs1550816 | C | T | C | T | -0.01107 | 0.00172 | 1.33E-10 | 0.005304 | 0.0142 | 0.7082 |  |  |  |  |
| rs1558727 | C | T | C | T | 0.01069 | 0.0017 | 3.09E-10 | 0.003496 | 0.0139 | 0.7993 |  |  |  |  |
| rs1566504 | T | C | T | C | 0.01126 | 0.00203 | 3.05E-08 | 0.011296 | 0.0169 | 0.5035 |  |  |  |  |
| rs1569092 | A | G | A | G | 0.01807 | 0.00234 | 1.16E-14 | 0.010999 | 0.0201 | 0.584 |  |  |  |  |
| rs1569723 | A | C | A | C | 0.01168 | 0.00197 | 3.01E-09 | 0.004201 | 0.0161 | 0.7934 |  |  |  |  |
| rs1584469 | T | C | T | C | -0.01303 | 0.00185 | 2.10E-12 | 0.012097 | 0.0151 | 0.4248 |  |  |  |  |
| rs1599381 | A | G | A | G | 0.01002 | 0.0017 | 3.83E-09 | 0.007899 | 0.0139 | 0.5719 |  |  |  |  |
| rs1618725 | T | C | T | C | 0.01477 | 0.00174 | 2.22E-17 | 0.023697 | 0.0138 | 0.08532 |  |  |  |  |
| rs1671770 | C | A | C | A | -0.01342 | 0.00223 | 1.91E-09 | -0.0118 | 0.0182 | 0.5144 |  |  |  |  |
| rs16854920 | T | C | T | C | -0.01007 | 0.00181 | 2.51E-08 | 0.004301 | 0.0155 | 0.7793 |  |  |  |  |
| rs17048855 | A | G | A | G | 0.01184 | 0.00179 | 3.27E-11 | -0.0274 | 0.0147 | 0.06236 |  |  |  |  |
| rs17060737 | C | T | C | T | -0.01192 | 0.0019 | 3.18E-10 | -0.0238 | 0.0154 | 0.1215 |  |  |  |  |
| rs17110109 | C | T | C | T | 0.01023 | 0.00175 | 4.71E-09 | -0.0141 | 0.0143 | 0.3245 |  |  |  |  |
| rs17126938 | T | C | T | C | -0.01536 | 0.0025 | 8.14E-10 | -0.0289 | 0.0201 | 0.1501 |  |  |  |  |
| rs17321729 | A | G | A | G | 0.01141 | 0.00196 | 6.06E-09 | 0.0003 | 0.0166 | 0.9861 |  |  |  |  |
| rs17425572 | G | A | G | A | -0.01224 | 0.0017 | 6.89E-13 | 0.005304 | 0.014 | 0.7032 |  |  |  |  |
| rs17489649 | A | G | A | G | 0.0139 | 0.00181 | 1.57E-14 | 0.016296 | 0.0147 | 0.2681 |  |  |  |  |
| rs17551064 | G | A | G | A | -0.01493 | 0.0023 | 8.62E-11 | 0.0078 | 0.0188 | 0.6774 |  |  |  |  |
| rs17563464 | A | C | A | C | -0.01477 | 0.00212 | 2.89E-12 | -0.0245 | 0.0183 | 0.1793 |  |  |  |  |
| rs17565975 | A | G | A | G | -0.01142 | 0.00171 | 2.56E-11 | -0.0056 | 0.0139 | 0.6876 |  |  |  |  |
| rs17570033 | G | T | G | T | 0.01862 | 0.00283 | 4.65E-11 | -0.0093 | 0.0225 | 0.6791 |  |  |  |  |
| rs17598675 | C | T | C | T | 0.01199 | 0.0017 | 1.75E-12 | 0.028399 | 0.0139 | 0.04131 |  |  |  |  |
| rs176218 | G | T | G | T | -0.01883 | 0.00215 | 1.85E-18 | -0.009 | 0.0174 | 0.606 |  |  |  |  |
| rs17638867 | T | C | T | C | 0.01329 | 0.00222 | 2.11E-09 | -0.0122 | 0.0176 | 0.4883 |  |  |  |  |
| rs1866823 | A | G | A | G | 0.01009 | 0.00171 | 3.81E-09 | -0.0001 | 0.0139 | 0.9971 |  |  |  |  |
| rs1880692 | A | G | A | G | 0.01008 | 0.0017 | 3.17E-09 | -0.0108 | 0.014 | 0.4404 |  |  |  |  |
| rs1892417 | C | T | C | T | -0.01732 | 0.00202 | 1.12E-17 | -0.0338 | 0.0162 | 0.03711 |  |  |  |  |
| rs1918394 | C | T | C | T | -0.01261 | 0.0023 | 4.15E-08 | 0.011304 | 0.0183 | 0.5374 |  |  |  |  |
| rs1931259 | A | G | A | G | -0.01237 | 0.00211 | 4.30E-09 | 0.016001 | 0.0168 | 0.3402 |  |  |  |  |
| rs1947114 | G | A | G | A | 0.01071 | 0.00192 | 2.64E-08 | 0.001301 | 0.0156 | 0.9356 |  |  |  |  |
| rs1952183 | G | A | G | A | 0.01055 | 0.0017 | 5.57E-10 | 0.010101 | 0.0142 | 0.4798 |  |  |  |  |
| rs1964927 | G | A | G | A | -0.01423 | 0.00177 | 9.90E-16 | -0.0041 | 0.0144 | 0.7789 |  |  |  |  |
| rs2002058 | C | T | C | T | 0.01211 | 0.00216 | 1.97E-08 | 0.013197 | 0.0181 | 0.4642 |  |  |  |  |
| rs2034670 | G | A | G | A | -0.01312 | 0.00214 | 8.09E-10 | -0.0066 | 0.0176 | 0.7057 |  |  |  |  |
| rs2052285 | A | G | A | G | 0.01123 | 0.00175 | 1.34E-10 | 0.008603 | 0.0142 | 0.5454 |  |  |  |  |
| rs2067854 | A | G | A | G | 0.01477 | 0.00209 | 1.38E-12 | 0.020498 | 0.0171 | 0.2287 |  |  |  |  |
| rs2141277 | A | G | A | G | 0.00952 | 0.0017 | 2.16E-08 | 0.022603 | 0.0139 | 0.1032 |  |  |  |  |
| rs2179152 | C | T | C | T | 0.01455 | 0.00176 | 1.21E-16 | 0.033298 | 0.0142 | 0.01887 |  |  |  |  |
| rs2182505 | T | C | T | C | 0.01086 | 0.00192 | 1.64E-08 | 0.018704 | 0.0153 | 0.2231 |  |  |  |  |
| rs2195086 | T | G | T | G | 0.01282 | 0.00228 | 2.00E-08 | 0.002497 | 0.0194 | 0.8983 |  |  |  |  |
| rs2220926 | T | C | T | C | -0.01055 | 0.00172 | 7.99E-10 | -0.0111 | 0.014 | 0.4282 |  |  |  |  |
| rs2245901 | A | G | A | G | -0.01403 | 0.00174 | 6.26E-16 | 0.009604 | 0.0141 | 0.4976 |  |  |  |  |
| rs2256965 | G | A | G | A | -0.01128 | 0.00176 | 1.59E-10 | 0.005304 | 0.0142 | 0.7075 |  |  |  |  |
| rs2283076 | A | G | A | G | 0.01143 | 0.00204 | 2.07E-08 | 0.001199 | 0.017 | 0.9448 |  |  |  |  |
| rs2302761 | T | C | T | C | 0.01354 | 0.00209 | 1.00E-10 | -0.0272 | 0.017 | 0.1093 |  |  |  |  |
| rs2336721 | C | T | C | T | -0.00996 | 0.0018 | 3.05E-08 | -0.0247 | 0.0146 | 0.0913 |  |  |  |  |
| rs2343094 | A | G | A | G | 0.01029 | 0.00181 | 1.29E-08 | -0.0175 | 0.0146 | 0.2311 |  |  |  |  |
| rs2347526 | T | C | T | C | -0.01395 | 0.00179 | 6.84E-15 | 0.027897 | 0.0148 | 0.05911 |  |  |  |  |
| rs2365376 | C | A | C | A | -0.0107 | 0.00179 | 2.15E-09 | -0.008 | 0.0146 | 0.5854 |  |  |  |  |
| rs2406253 | G | A | G | A | -0.01411 | 0.00216 | 6.40E-11 | -0.0223 | 0.0176 | 0.2047 |  |  |  |  |
| rs242093 | A | G | A | G | -0.01031 | 0.00172 | 2.07E-09 | 0.004102 | 0.0143 | 0.7744 |  |  |  |  |
| rs2447535 | A | G | A | G | -0.01181 | 0.00185 | 1.69E-10 | 0.0285 | 0.015 | 0.05679 |  |  |  |  |
| rs2496482 | C | T | C | T | -0.01109 | 0.00177 | 4.04E-10 | 0.007599 | 0.0143 | 0.5929 |  |  |  |  |
| rs2554835 | G | A | G | A | -0.00974 | 0.00175 | 2.69E-08 | -0.0076 | 0.0144 | 0.5964 |  |  |  |  |
| rs2589091 | G | A | G | A | 0.00949 | 0.00172 | 3.26E-08 | 0.016902 | 0.014 | 0.2282 |  |  |  |  |
| rs268120 | A | G | A | G | 0.01244 | 0.00196 | 2.13E-10 | -0.0077 | 0.0156 | 0.6205 |  |  |  |  |
| rs2706762 | T | C | T | C | 0.01484 | 0.00246 | 1.59E-09 | 0.0227 | 0.0198 | 0.2515 |  |  |  |  |
| rs2725370 | T | C | T | C | -0.01536 | 0.00187 | 1.97E-16 | 0.039201 | 0.0152 | 0.009881 |  |  |  |  |
| rs273438 | G | A | G | A | 0.00937 | 0.00171 | 4.15E-08 | -0.014 | 0.0138 | 0.3109 |  |  |  |  |
| rs277828 | C | A | C | A | 0.01091 | 0.00196 | 2.71E-08 | -0.0044 | 0.0165 | 0.7907 |  |  |  |  |
| rs2787101 | C | T | C | T | -0.00968 | 0.00174 | 2.50E-08 | 0.017095 | 0.0142 | 0.2292 |  |  |  |  |
| rs281302 | A | G | A | G | -0.0111 | 0.00172 | 9.84E-11 | -0.0055 | 0.0142 | 0.6985 |  |  |  |  |
| rs2819336 | T | C | T | C | 0.01828 | 0.00177 | 5.46E-25 | 0.0063 | 0.0145 | 0.6629 |  |  |  |  |
| rs2820314 | A | C | A | C | 0.011 | 0.0018 | 9.34E-10 | -0.0108 | 0.0147 | 0.4627 |  |  |  |  |
| rs28513670 | G | A | G | A | 0.01477 | 0.00225 | 5.06E-11 | -0.0005 | 0.0183 | 0.9761 |  |  |  |  |
| rs28513882 | G | A | G | A | 0.01246 | 0.00221 | 1.63E-08 | -0.0016 | 0.0185 | 0.9313 |  |  |  |  |
| rs28661002 | T | C | T | C | 0.01099 | 0.00198 | 2.89E-08 | 0.0118 | 0.0162 | 0.4664 |  |  |  |  |
| rs2885198 | A | G | A | G | 0.01025 | 0.0017 | 1.81E-09 | -0.0022 | 0.0141 | 0.8782 |  |  |  |  |
| rs2898191 | A | C | A | C | 0.01041 | 0.00188 | 3.29E-08 | -0.0075 | 0.015 | 0.6188 |  |  |  |  |
| rs2964197 | T | C | T | C | 0.01177 | 0.0017 | 4.70E-12 | 0.021801 | 0.0139 | 0.1173 |  |  |  |  |
| rs2998315 | G | A | G | A | 0.01269 | 0.00171 | 1.12E-13 | 0.020601 | 0.0143 | 0.1495 |  |  |  |  |
| rs301800 | T | C | T | C | 0.01516 | 0.00224 | 1.33E-11 | 0.016296 | 0.0183 | 0.3743 |  |  |  |  |
| rs3026996 | A | C | A | C | 0.01537 | 0.00199 | 1.05E-14 | 0.038903 | 0.0167 | 0.0196 |  |  |  |  |
| rs31940 | G | A | G | A | -0.01548 | 0.00246 | 3.24E-10 | -0.0123 | 0.0202 | 0.544 |  |  |  |  |
| rs337637 | G | A | G | A | -0.01123 | 0.00177 | 2.11E-10 | -0.0069 | 0.0145 | 0.6328 |  |  |  |  |
| rs339054 | G | T | G | T | 0.0117 | 0.0017 | 5.90E-12 | -0.0071 | 0.0138 | 0.6076 |  |  |  |  |
| rs34316 | C | A | C | A | -0.02016 | 0.00177 | 3.35E-30 | -0.0068 | 0.0142 | 0.6298 |  |  |  |  |
| rs34394051 | G | A | G | A | 0.01392 | 0.0024 | 6.20E-09 | -0.016 | 0.0191 | 0.401 |  |  |  |  |
| rs34485537 | T | C | T | C | 0.01075 | 0.00173 | 5.67E-10 | 0.0007 | 0.0143 | 0.9612 |  |  |  |  |
| rs35039375 | G | A | G | A | -0.01983 | 0.00293 | 1.22E-11 | -0.0119 | 0.0251 | 0.6357 |  |  |  |  |
| rs35309068 | G | T | G | T | 0.01321 | 0.00171 | 1.15E-14 | 0.001701 | 0.0139 | 0.9004 |  |  |  |  |
| rs35316276 | C | T | C | T | -0.01173 | 0.00194 | 1.52E-09 | 0.028996 | 0.0158 | 0.06546 |  |  |  |  |
| rs35417702 | C | T | C | T | 0.01445 | 0.0017 | 1.93E-17 | -0.0292 | 0.0139 | 0.03479 |  |  |  |  |
| rs35475880 | G | T | G | T | 0.01511 | 0.00208 | 3.80E-13 | 0.014403 | 0.0171 | 0.4012 |  |  |  |  |
| rs36083520 | C | T | C | T | 0.01629 | 0.00223 | 2.60E-13 | -0.0284 | 0.0184 | 0.1225 |  |  |  |  |
| rs36119825 | G | A | G | A | -0.01063 | 0.00171 | 4.82E-10 | -0.0037 | 0.0139 | 0.7891 |  |  |  |  |
| rs363096 | C | T | C | T | 0.01363 | 0.00172 | 2.04E-15 | -0.0026 | 0.014 | 0.8503 |  |  |  |  |
| rs3781339 | T | C | T | C | -0.01247 | 0.00219 | 1.30E-08 | 0.019999 | 0.0177 | 0.2597 |  |  |  |  |
| rs3796348 | G | A | G | A | -0.01032 | 0.00175 | 3.66E-09 | -0.0136 | 0.0144 | 0.3447 |  |  |  |  |
| rs3809634 | A | G | A | G | -0.01058 | 0.00185 | 1.09E-08 | 0.0007 | 0.0149 | 0.964 |  |  |  |  |
| rs3812281 | C | T | C | T | -0.01228 | 0.00174 | 1.58E-12 | 0.003295 | 0.0142 | 0.8178 |  |  |  |  |
| rs3890802 | A | G | A | G | -0.01133 | 0.00191 | 2.74E-09 | 0.006896 | 0.0153 | 0.6505 |  |  |  |  |
| rs3897821 | A | G | A | G | 0.01502 | 0.0018 | 8.25E-17 | 0.001798 | 0.0146 | 0.8992 |  |  |  |  |
| rs4073894 | A | G | A | G | 0.01524 | 0.00211 | 5.40E-13 | -0.0113 | 0.0175 | 0.5186 |  |  |  |  |
| rs4144624 | C | T | C | T | 0.01338 | 0.00239 | 2.30E-08 | -0.0197 | 0.0203 | 0.3333 |  |  |  |  |
| rs4328757 | T | C | T | C | 0.01067 | 0.00174 | 9.39E-10 | 0.013903 | 0.0141 | 0.3263 |  |  |  |  |
| rs4352658 | T | C | T | C | -0.0212 | 0.00308 | 5.55E-12 | -0.0253 | 0.0266 | 0.342 |  |  |  |  |
| rs4382592 | T | G | T | G | -0.01636 | 0.00185 | 1.01E-18 | 0.005296 | 0.015 | 0.7238 |  |  |  |  |
| rs4384309 | G | A | G | A | -0.0109 | 0.00172 | 2.52E-10 | -0.0271 | 0.0143 | 0.05833 |  |  |  |  |
| rs4641552 | C | A | C | A | -0.01926 | 0.00336 | 1.02E-08 | -0.0105 | 0.0276 | 0.7033 |  |  |  |  |
| rs4652135 | C | A | C | A | -0.01219 | 0.0019 | 1.54E-10 | -0.0229 | 0.0152 | 0.131 |  |  |  |  |
| rs4675248 | G | A | G | A | 0.01004 | 0.00173 | 6.75E-09 | -0.009 | 0.0142 | 0.5272 |  |  |  |  |
| rs4700393 | G | A | G | A | 0.02086 | 0.0017 | 1.51E-34 | 0.0185 | 0.0138 | 0.1801 |  |  |  |  |
| rs4726070 | G | A | G | A | -0.01251 | 0.00174 | 5.95E-13 | -0.0046 | 0.014 | 0.7443 |  |  |  |  |
| rs4731413 | G | A | G | A | -0.01211 | 0.00213 | 1.37E-08 | -0.0214 | 0.0179 | 0.2325 |  |  |  |  |
| rs4760687 | G | A | G | A | 0.01039 | 0.00183 | 1.30E-08 | -0.0212 | 0.0151 | 0.1597 |  |  |  |  |
| rs4778058 | C | T | C | T | 0.01017 | 0.0017 | 2.40E-09 | 0.003596 | 0.0141 | 0.8009 |  |  |  |  |
| rs4780563 | G | A | G | A | 0.01398 | 0.00245 | 1.12E-08 | 0.023596 | 0.0197 | 0.2319 |  |  |  |  |
| rs4787457 | G | A | G | A | -0.01741 | 0.00176 | 3.73E-23 | 0.002603 | 0.0143 | 0.8563 |  |  |  |  |
| rs4810227 | A | G | A | G | 0.01272 | 0.00175 | 3.57E-13 | 0.026398 | 0.0143 | 0.06557 |  |  |  |  |
| rs4839155 | T | G | T | G | 0.01251 | 0.002 | 3.94E-10 | 0.002497 | 0.0162 | 0.8786 |  |  |  |  |
| rs4846724 | G | A | G | A | -0.01018 | 0.0017 | 2.26E-09 | -0.0005 | 0.0139 | 0.9707 |  |  |  |  |
| rs4858670 | C | T | C | T | 0.0101 | 0.00183 | 3.22E-08 | -0.0006 | 0.0147 | 0.9698 |  |  |  |  |
| rs4860734 | A | G | A | G | -0.0114 | 0.00188 | 1.29E-09 | 0.0008 | 0.0155 | 0.9566 |  |  |  |  |
| rs4895650 | T | C | T | C | 0.00965 | 0.00172 | 2.16E-08 | -0.0076 | 0.0142 | 0.5915 |  |  |  |  |
| rs4904523 | G | A | G | A | 0.00936 | 0.0017 | 3.71E-08 | 0.002804 | 0.014 | 0.8429 |  |  |  |  |
| rs4964046 | G | A | G | A | 0.01053 | 0.00178 | 3.36E-09 | 0.012498 | 0.0145 | 0.3892 |  |  |  |  |
| rs4972400 | G | A | G | A | -0.01156 | 0.00181 | 1.70E-10 | -0.0172 | 0.0149 | 0.2482 |  |  |  |  |
| rs4976445 | T | C | T | C | 0.012 | 0.00197 | 1.16E-09 | -0.0055 | 0.016 | 0.732 |  |  |  |  |
| rs4984541 | A | G | A | G | -0.01233 | 0.00207 | 2.77E-09 | -0.0224 | 0.0175 | 0.2011 |  |  |  |  |
| rs535307 | A | G | A | G | 0.01004 | 0.00184 | 4.73E-08 | -0.0251 | 0.0152 | 0.0991 |  |  |  |  |
| rs55641816 | T | C | T | C | -0.01462 | 0.00262 | 2.40E-08 | 0.002098 | 0.0199 | 0.9162 |  |  |  |  |
| rs56048629 | T | C | T | C | -0.01654 | 0.00176 | 6.76E-21 | -0.0172 | 0.0144 | 0.2317 |  |  |  |  |
| rs56391344 | A | G | A | G | 0.01571 | 0.00197 | 1.34E-15 | 0.006797 | 0.0159 | 0.6702 |  |  |  |  |
| rs563954 | A | G | A | G | -0.00976 | 0.00175 | 2.29E-08 | -0.0037 | 0.0139 | 0.7879 |  |  |  |  |
| rs57016874 | C | T | C | T | -0.02902 | 0.00457 | 2.09E-10 | -0.0848 | 0.0379 | 0.02519 |  |  |  |  |
| rs5763431 | T | C | T | C | 0.01125 | 0.00176 | 1.53E-10 | 0.027099 | 0.0143 | 0.05797 |  |  |  |  |
| rs59123361 | A | G | A | G | -0.02094 | 0.00291 | 5.87E-13 | -0.0087 | 0.024 | 0.7181 |  |  |  |  |
| rs59484001 | C | T | C | T | 0.02926 | 0.00391 | 7.42E-14 | -0.0188 | 0.0346 | 0.5862 |  |  |  |  |
| rs60096640 | G | A | G | A | -0.01574 | 0.00273 | 7.82E-09 | 0.015205 | 0.0215 | 0.48 |  |  |  |  |
| rs6067645 | A | G | A | G | -0.01003 | 0.00173 | 6.38E-09 | 0.001499 | 0.0153 | 0.9212 |  |  |  |  |
| rs6122735 | T | C | T | C | 0.0105 | 0.00174 | 1.49E-09 | 0.014199 | 0.014 | 0.3122 |  |  |  |  |
| rs6123924 | A | G | A | G | 0.01528 | 0.00235 | 7.55E-11 | 0.031499 | 0.0189 | 0.09604 |  |  |  |  |
| rs613872 | G | T | G | T | 0.0175 | 0.00227 | 1.20E-14 | 0.021203 | 0.0182 | 0.2439 |  |  |  |  |
| rs61996546 | C | T | C | T | -0.00965 | 0.0017 | 1.34E-08 | -0.0078 | 0.0138 | 0.5724 |  |  |  |  |
| rs62142891 | G | A | G | A | -0.01121 | 0.00194 | 6.94E-09 | -0.0034 | 0.0157 | 0.8273 |  |  |  |  |
| rs62155873 | T | C | T | C | -0.01441 | 0.00259 | 2.58E-08 | -0.0265 | 0.0217 | 0.2218 |  |  |  |  |
| rs62157915 | T | C | T | C | -0.02091 | 0.00348 | 1.96E-09 | -0.0253 | 0.0282 | 0.3692 |  |  |  |  |
| rs62166492 | A | G | A | G | 0.02846 | 0.0034 | 6.03E-17 | 0.066097 | 0.0276 | 0.01641 |  |  |  |  |
| rs62172885 | T | C | T | C | -0.01012 | 0.00179 | 1.73E-08 | 0.010999 | 0.0146 | 0.4524 |  |  |  |  |
| rs62174974 | G | A | G | A | 0.01191 | 0.00215 | 2.83E-08 | -0.0229 | 0.0176 | 0.1925 |  |  |  |  |
| rs62177359 | A | C | A | C | -0.0304 | 0.00475 | 1.60E-10 | 0.011197 | 0.033 | 0.7337 |  |  |  |  |
| rs62184480 | T | C | T | C | -0.01528 | 0.00191 | 1.28E-15 | -0.012 | 0.0153 | 0.4342 |  |  |  |  |
| rs62190914 | T | C | T | C | 0.01001 | 0.00176 | 1.38E-08 | -0.0144 | 0.0143 | 0.3119 |  |  |  |  |
| rs622169 | C | T | C | T | -0.00999 | 0.00178 | 1.89E-08 | -0.0081 | 0.0163 | 0.6199 |  |  |  |  |
| rs62439690 | G | A | G | A | 0.01087 | 0.00194 | 2.18E-08 | 0.003105 | 0.0164 | 0.8481 |  |  |  |  |
| rs62444881 | C | T | C | T | -0.01815 | 0.00217 | 5.79E-17 | 0.023299 | 0.0175 | 0.1851 |  |  |  |  |
| rs6429082 | C | T | C | T | 0.01078 | 0.0017 | 2.37E-10 | 0.020703 | 0.0139 | 0.1357 |  |  |  |  |
| rs6436555 | C | A | C | A | -0.01012 | 0.0017 | 2.70E-09 | -0.0204 | 0.014 | 0.1454 |  |  |  |  |
| rs6440008 | T | C | T | C | -0.00976 | 0.00175 | 2.44E-08 | 0.001099 | 0.0144 | 0.9371 |  |  |  |  |
| rs6457996 | C | T | C | T | -0.01014 | 0.0017 | 2.43E-09 | 0.013896 | 0.014 | 0.3224 |  |  |  |  |
| rs6493265 | C | T | C | T | 0.01385 | 0.00174 | 1.70E-15 | -0.0172 | 0.014 | 0.2186 |  |  |  |  |
| rs6513959 | A | G | A | G | 0.01177 | 0.00185 | 1.88E-10 | -0.0065 | 0.0151 | 0.6669 |  |  |  |  |
| rs6535149 | T | C | T | C | -0.01051 | 0.00189 | 2.62E-08 | -0.0217 | 0.0155 | 0.1616 |  |  |  |  |
| rs6557171 | C | T | C | T | 0.01567 | 0.00181 | 4.15E-18 | -0.0005 | 0.0148 | 0.9725 |  |  |  |  |
| rs6573552 | T | C | T | C | -0.01086 | 0.0017 | 1.62E-10 | -0.019 | 0.0138 | 0.1695 |  |  |  |  |
| rs66568921 | G | T | G | T | 0.01565 | 0.00182 | 7.49E-18 | -0.0007 | 0.0145 | 0.9624 |  |  |  |  |
| rs66641143 | T | C | T | C | -0.03162 | 0.00486 | 8.04E-11 | -0.0076 | 0.0395 | 0.8474 |  |  |  |  |
| rs6666119 | A | G | A | G | -0.01269 | 0.00183 | 4.16E-12 | -0.0136 | 0.0149 | 0.363 |  |  |  |  |
| rs66671632 | C | T | C | T | 0.01838 | 0.00254 | 4.59E-13 | -0.0133 | 0.0215 | 0.5351 |  |  |  |  |
| rs6736898 | A | G | A | G | 0.01032 | 0.00178 | 6.09E-09 | -0.0064 | 0.0145 | 0.6606 |  |  |  |  |
| rs67885444 | T | C | T | C | 0.01406 | 0.00232 | 1.48E-09 | 0.015499 | 0.0195 | 0.4256 |  |  |  |  |
| rs67890737 | C | A | C | A | 0.01141 | 0.00179 | 2.01E-10 | 0.0003 | 0.0144 | 0.9829 |  |  |  |  |
| rs6805241 | C | T | C | T | -0.01413 | 0.00203 | 3.09E-12 | -0.0093 | 0.0167 | 0.5772 |  |  |  |  |
| rs6871635 | A | G | A | G | -0.00946 | 0.00173 | 4.63E-08 | -0.0207 | 0.0142 | 0.1444 |  |  |  |  |
| rs6959891 | G | A | G | A | -0.01136 | 0.00189 | 1.74E-09 | 0.019295 | 0.0151 | 0.2018 |  |  |  |  |
| rs7012546 | C | T | C | T | -0.01009 | 0.00172 | 4.93E-09 | -0.0209 | 0.014 | 0.135 |  |  |  |  |
| rs702606 | T | C | T | C | 0.01427 | 0.0025 | 1.12E-08 | 0.0007 | 0.0216 | 0.9751 |  |  |  |  |
| rs7029718 | G | A | G | A | -0.02439 | 0.00174 | 1.85E-44 | -0.0398 | 0.0141 | 0.0046 |  |  |  |  |
| rs710629 | A | G | A | G | 0.01053 | 0.00177 | 2.96E-09 | -0.0015 | 0.0143 | 0.914 |  |  |  |  |
| rs7108020 | C | A | C | A | -0.01094 | 0.00178 | 7.35E-10 | -0.0215 | 0.0145 | 0.1391 |  |  |  |  |
| rs7136760 | A | G | A | G | 0.01018 | 0.00177 | 9.36E-09 | -0.0231 | 0.0145 | 0.1126 |  |  |  |  |
| rs7139165 | C | A | C | A | -0.01136 | 0.00195 | 5.29E-09 | -0.0219 | 0.0151 | 0.1479 |  |  |  |  |
| rs71432775 | A | G | A | G | 0.0113 | 0.00198 | 1.06E-08 | -0.0017 | 0.0157 | 0.9133 |  |  |  |  |
| rs71646142 | C | T | C | T | -0.01286 | 0.00217 | 3.11E-09 | -0.0017 | 0.0178 | 0.924 |  |  |  |  |
| rs7188873 | A | G | A | G | 0.0106 | 0.00175 | 1.34E-09 | 0.026905 | 0.0142 | 0.05695 |  |  |  |  |
| rs7215889 | T | C | T | C | 0.01132 | 0.00196 | 7.06E-09 | -0.0067 | 0.016 | 0.677 |  |  |  |  |
| rs7233920 | A | G | A | G | -0.01315 | 0.00202 | 7.13E-11 | -0.0124 | 0.0167 | 0.4583 |  |  |  |  |
| rs7257460 | T | C | T | C | 0.01145 | 0.00189 | 1.25E-09 | -0.0144 | 0.0152 | 0.3456 |  |  |  |  |
| rs72677177 | G | A | G | A | -0.0105 | 0.00174 | 1.55E-09 | -0.0042 | 0.0142 | 0.7656 |  |  |  |  |
| rs72686126 | C | T | C | T | 0.01862 | 0.00331 | 1.88E-08 | 0.011496 | 0.0313 | 0.7127 |  |  |  |  |
| rs72693550 | C | A | C | A | 0.01542 | 0.00234 | 4.57E-11 | -0.0037 | 0.0198 | 0.8503 |  |  |  |  |
| rs728054 | G | A | G | A | 0.01274 | 0.00177 | 6.65E-13 | 0.029398 | 0.0147 | 0.04576 |  |  |  |  |
| rs72829857 | G | A | G | A | 0.01516 | 0.00202 | 5.39E-14 | -0.0173 | 0.0163 | 0.2882 |  |  |  |  |
| rs72840994 | G | T | G | T | 0.01247 | 0.00216 | 7.77E-09 | 0.014302 | 0.0176 | 0.4159 |  |  |  |  |
| rs7321274 | G | A | G | A | -0.01275 | 0.00211 | 1.59E-09 | -0.0018 | 0.0168 | 0.9155 |  |  |  |  |
| rs73301698 | A | G | A | G | -0.01291 | 0.00208 | 5.81E-10 | -0.0047 | 0.0172 | 0.7844 |  |  |  |  |
| rs7332724 | T | C | T | C | -0.01149 | 0.00189 | 1.26E-09 | 0.021096 | 0.0152 | 0.1652 |  |  |  |  |
| rs73344830 | G | A | G | A | -0.0172 | 0.00172 | 1.95E-23 | -0.0052 | 0.0142 | 0.7141 |  |  |  |  |
| rs73874335 | C | T | C | T | 0.0199 | 0.00361 | 3.40E-08 | 0.013703 | 0.0303 | 0.6507 |  |  |  |  |
| rs743316 | T | C | T | C | 0.01185 | 0.00208 | 1.20E-08 | -0.0141 | 0.017 | 0.4071 |  |  |  |  |
| rs74701752 | G | T | G | T | -0.01591 | 0.00285 | 2.38E-08 | -0.009 | 0.0228 | 0.6928 |  |  |  |  |
| rs74944275 | C | T | C | T | -0.02739 | 0.00424 | 1.02E-10 | -0.0618 | 0.0352 | 0.07905 |  |  |  |  |
| rs7526112 | T | G | T | G | 0.01215 | 0.00177 | 6.10E-12 | 0.016601 | 0.0145 | 0.2509 |  |  |  |  |
| rs75708852 | A | C | A | C | -0.02792 | 0.00498 | 2.09E-08 | -0.0175 | 0.0374 | 0.6405 |  |  |  |  |
| rs75755471 | A | G | A | G | -0.01891 | 0.00344 | 3.79E-08 | -0.0069 | 0.0278 | 0.8023 |  |  |  |  |
| rs7575637 | A | G | A | G | 0.01136 | 0.00171 | 2.86E-11 | 0.016601 | 0.0139 | 0.2337 |  |  |  |  |
| rs7595950 | C | T | C | T | -0.00993 | 0.0017 | 4.99E-09 | 0.021898 | 0.014 | 0.1168 |  |  |  |  |
| rs7597126 | C | T | C | T | 0.01009 | 0.00172 | 4.20E-09 | 0.008304 | 0.0142 | 0.5597 |  |  |  |  |
| rs7603132 | G | A | G | A | -0.01317 | 0.00215 | 9.17E-10 | 0.007196 | 0.0179 | 0.6872 |  |  |  |  |
| rs77025239 | A | G | A | G | -0.01422 | 0.00234 | 1.33E-09 | -0.0073 | 0.0192 | 0.7043 |  |  |  |  |
| rs77128898 | T | C | T | C | -0.02769 | 0.00482 | 9.47E-09 | 0.060399 | 0.0529 | 0.253 |  |  |  |  |
| rs7737905 | T | G | T | G | -0.01285 | 0.00188 | 8.34E-12 | 0.007601 | 0.0157 | 0.6285 |  |  |  |  |
| rs77702819 | G | T | G | T | -0.01863 | 0.00298 | 3.92E-10 | -0.0122 | 0.0256 | 0.6342 |  |  |  |  |
| rs77835879 | A | G | A | G | 0.01601 | 0.00288 | 2.68E-08 | -0.0265 | 0.0257 | 0.303 |  |  |  |  |
| rs7788620 | G | A | G | A | -0.01635 | 0.00206 | 1.84E-15 | 0.008496 | 0.0165 | 0.6077 |  |  |  |  |
| rs7803932 | A | G | A | G | 0.0143 | 0.00226 | 2.44E-10 | 0.006896 | 0.0189 | 0.7141 |  |  |  |  |
| rs790647 | C | A | C | A | 0.01482 | 0.00202 | 2.17E-13 | 0.004601 | 0.0166 | 0.7812 |  |  |  |  |
| rs7924036 | T | G | T | G | 0.01501 | 0.0017 | 1.07E-18 | 0.0118 | 0.0138 | 0.3919 |  |  |  |  |
| rs79265434 | A | G | A | G | -0.02331 | 0.00262 | 6.08E-19 | -0.0532 | 0.0224 | 0.0173 |  |  |  |  |
| rs79269403 | G | A | G | A | -0.01447 | 0.00204 | 1.17E-12 | 0.025102 | 0.0173 | 0.146 |  |  |  |  |
| rs7928017 | C | A | C | A | -0.00953 | 0.00172 | 2.83E-08 | -0.0144 | 0.014 | 0.304 |  |  |  |  |
| rs79375112 | G | A | G | A | -0.01529 | 0.00257 | 2.63E-09 | -0.0061 | 0.0212 | 0.773 |  |  |  |  |
| rs7943853 | C | T | C | T | -0.01128 | 0.00201 | 2.06E-08 | 0.001001 | 0.0165 | 0.9535 |  |  |  |  |
| rs795230 | T | C | T | C | 0.00952 | 0.00172 | 2.97E-08 | 0.008702 | 0.0139 | 0.5302 |  |  |  |  |
| rs79523955 | A | G | A | G | 0.01802 | 0.00283 | 1.87E-10 | 0.019901 | 0.0235 | 0.3966 |  |  |  |  |
| rs7977614 | G | A | G | A | 0.01325 | 0.00198 | 2.09E-11 | -0.0233 | 0.0184 | 0.2042 |  |  |  |  |
| rs8008382 | T | C | T | C | -0.01208 | 0.00185 | 6.12E-11 | -0.02 | 0.0152 | 0.1864 |  |  |  |  |
| rs80171383 | A | G | A | G | 0.0145 | 0.00241 | 1.83E-09 | 0.033802 | 0.0203 | 0.09532 |  |  |  |  |
| rs8052297 | T | G | T | G | -0.01029 | 0.00172 | 2.10E-09 | -0.0079 | 0.014 | 0.5733 |  |  |  |  |
| rs818415 | T | G | T | G | -0.01235 | 0.00219 | 1.72E-08 | -0.0176 | 0.0178 | 0.3242 |  |  |  |  |
| rs892612 | C | A | C | A | 0.01464 | 0.00237 | 6.63E-10 | 0.020203 | 0.0195 | 0.3003 |  |  |  |  |
| rs9289300 | T | C | T | C | -0.01512 | 0.00234 | 1.10E-10 | -0.01521 | 0.0191 | 0.4262 |  |  |  |  |
| rs9320493 | G | A | G | A | -0.01394 | 0.0024 | 6.13E-09 | 0.023596 | 0.0202 | 0.2431 |  |  |  |  |
| rs9342482 | T | G | T | G | 0.01264 | 0.00197 | 1.36E-10 | -0.0064 | 0.0161 | 0.6914 |  |  |  |  |
| rs9349956 | A | C | A | C | -0.01881 | 0.00225 | 6.28E-17 | -0.02599 | 0.0184 | 0.1559 |  |  |  |  |
| rs9372625 | A | G | A | G | 0.02383 | 0.00176 | 6.76E-42 | 0.029801 | 0.0145 | 0.04039 |  |  |  |  |
| rs9384679 | T | C | T | C | -0.00959 | 0.00176 | 4.88E-08 | -0.0187 | 0.0145 | 0.1955 |  |  |  |  |
| rs9388490 | T | C | T | C | 0.00972 | 0.00171 | 1.43E-08 | -0.0089 | 0.0139 | 0.524 |  |  |  |  |
| rs939400 | G | T | G | T | 0.01031 | 0.00177 | 5.28E-09 | -0.0044 | 0.0143 | 0.7605 |  |  |  |  |
| rs9436866 | C | A | C | A | 0.01882 | 0.00289 | 7.45E-11 | 0.029903 | 0.0243 | 0.2192 |  |  |  |  |
| rs9503598 | A | G | A | G | 0.01079 | 0.00171 | 3.12E-10 | 0.0167 | 0.014 | 0.2335 |  |  |  |  |
| rs9513416 | G | A | G | A | 0.01316 | 0.00232 | 1.46E-08 | 0.016495 | 0.0193 | 0.3945 |  |  |  |  |
| rs9536961 | A | G | A | G | -0.01242 | 0.0018 | 4.96E-12 | -0.003 | 0.0147 | 0.8367 |  |  |  |  |
| rs9556958 | C | T | C | T | 0.0108 | 0.0017 | 2.38E-10 | -0.0098 | 0.0138 | 0.4804 |  |  |  |  |
| rs9616906 | G | A | G | A | -0.01497 | 0.00172 | 2.92E-18 | 0.005304 | 0.0139 | 0.701 |  |  |  |  |
| rs9704097 | A | C | A | C | -0.0103 | 0.00171 | 1.61E-09 | -0.002 | 0.0139 | 0.8852 |  |  |  |  |
| rs9771228 | T | C | T | C | 0.01182 | 0.00178 | 3.07E-11 | -0.0166 | 0.0146 | 0.2548 |  |  |  |  |
| rs9914918 | G | A | G | A | -0.01155 | 0.00189 | 8.90E-10 | 0.006099 | 0.0154 | 0.6904 |  |  |  |  |
| rs9936270 | T | C | T | C | -0.0136 | 0.00198 | 6.43E-12 | 0.0305 | 0.0162 | 0.06026 |  |  |  |  |
| rs9964724 | C | T | C | T | -0.01978 | 0.00183 | 2.66E-27 | 0.001301 | 0.0148 | 0.9311 |  |  |  |  |
| rs997123 | C | T | C | T | 0.00966 | 0.0017 | 1.32E-08 | 0.011304 | 0.0139 | 0.4164 |  |  |  |  |
| rs998887 | A | C | A | C | 0.01272 | 0.0017 | 7.42E-14 | -0.03 | 0.0139 | 0.03047 |  |  |  |  |
| rs10205801 | A | G | A | G | -0.01053 | 0.00171 | 7.17E-10 | 0.031101 | 0.0139 | 0.02525 | *Removed during Steiger filtering | | | |
| rs10215082 | A | G | A | G | -0.01303 | 0.00172 | 3.33E-14 | 0.036602 | 0.0139 | 0.008287 |  |  |  |  |
| rs10402747 | C | T | C | T | -0.0097 | 0.00172 | 1.73E-08 | -0.0292 | 0.0143 | 0.04172 |  |  |  |  |
| rs11081529 | T | C | T | C | 0.01311 | 0.00186 | 1.82E-12 | -0.0362 | 0.0154 | 0.01837 |  |  |  |  |
| rs11623285 | G | T | G | T | 0.01413 | 0.00251 | 1.72E-08 | 0.048098 | 0.0225 | 0.03249 |  |  |  |  |
| rs11663602 | A | C | A | C | -0.01213 | 0.0019 | 1.64E-10 | 0.031896 | 0.0156 | 0.04043 |  |  |  |  |
| rs11754551 | C | T | C | T | 0.01627 | 0.00272 | 2.08E-09 | 0.045803 | 0.0224 | 0.04066 |  |  |  |  |
| rs11774212 | T | C | T | C | 0.01196 | 0.00171 | 2.74E-12 | -0.0498 | 0.0152 | 0.001066 |  |  |  |  |
| rs12030427 | G | A | G | A | -0.01085 | 0.00198 | 4.34E-08 | -0.0283 | 0.0162 | 0.08073 |  |  |  |  |
| rs12908232 | A | G | A | G | 0.00975 | 0.0017 | 1.00E-08 | 0.028199 | 0.0138 | 0.04124 |  |  |  |  |
| rs13163062 | T | C | T | C | 0.01007 | 0.00172 | 4.71E-09 | 0.040095 | 0.014 | 0.004313 |  |  |  |  |
| rs1364626 | C | T | C | T | -0.00956 | 0.00171 | 2.15E-08 | -0.0299 | 0.0139 | 0.03212 |  |  |  |  |
| rs1381247 | C | T | C | T | -0.01013 | 0.00182 | 2.46E-08 | 0.037401 | 0.0155 | 0.01611 |  |  |  |  |
| rs1427298 | C | T | C | T | -0.0102 | 0.00172 | 3.28E-09 | -0.0343 | 0.0141 | 0.01529 |  |  |  |  |
| rs143163770 | T | C | T | C | -0.01625 | 0.00258 | 3.22E-10 | -0.0674 | 0.0228 | 0.003107 |  |  |  |  |
| rs1461515 | G | A | G | A | -0.0093 | 0.0017 | 4.55E-08 | -0.0271 | 0.0138 | 0.04892 |  |  |  |  |
| rs152603 | G | A | G | A | 0.01019 | 0.00177 | 9.47E-09 | 0.028996 | 0.0147 | 0.0483 |  |  |  |  |
| rs1566085 | T | G | T | G | 0.01645 | 0.00171 | 6.90E-22 | 0.054602 | 0.0141 | 0.000104 |  |  |  |  |
| rs1620977 | G | A | G | A | -0.02046 | 0.00195 | 1.14E-25 | -0.0624 | 0.0162 | 0.000119 |  |  |  |  |
| rs16822665 | T | C | T | C | 0.01286 | 0.00185 | 3.57E-12 | 0.043404 | 0.0147 | 0.003093 |  |  |  |  |
| rs16995054 | C | T | C | T | 0.0139 | 0.00208 | 2.52E-11 | 0.040104 | 0.0165 | 0.0149 |  |  |  |  |
| rs1758747 | A | G | A | G | 0.0101 | 0.00184 | 4.13E-08 | -0.0294 | 0.0151 | 0.05165 |  |  |  |  |
| rs1827540 | G | A | G | A | -0.0106 | 0.0017 | 4.50E-10 | -0.0282 | 0.0138 | 0.04085 |  |  |  |  |
| rs192436652 | C | T | C | T | 0.03497 | 0.00545 | 1.35E-10 | 0.083001 | 0.0449 | 0.06469 |  |  |  |  |
| rs1925587 | C | T | C | T | -0.01016 | 0.00171 | 3.03E-09 | 0.027104 | 0.0139 | 0.05146 |  |  |  |  |
| rs1979969 | G | T | G | T | 0.01208 | 0.00195 | 6.17E-10 | 0.036104 | 0.0153 | 0.01818 |  |  |  |  |
| rs2287838 | A | G | A | G | -0.01152 | 0.00171 | 1.53E-11 | -0.0324 | 0.0139 | 0.01944 |  |  |  |  |
| rs2297600 | G | T | G | T | -0.01569 | 0.00223 | 2.16E-12 | 0.042302 | 0.0179 | 0.01809 |  |  |  |  |
| rs2702575 | C | T | C | T | 0.00989 | 0.00175 | 1.56E-08 | 0.0254 | 0.0143 | 0.07455 |  |  |  |  |
| rs2833483 | C | T | C | T | 0.01974 | 0.00337 | 4.77E-09 | 0.087499 | 0.028 | 0.001748 |  |  |  |  |
| rs2971970 | T | G | T | G | -0.01654 | 0.00207 | 1.25E-15 | -0.0465 | 0.0171 | 0.006599 |  |  |  |  |
| rs35104491 | A | G | A | G | 0.01218 | 0.00219 | 2.62E-08 | 0.039999 | 0.0176 | 0.02341 |  |  |  |  |
| rs35919256 | C | A | C | A | -0.01032 | 0.00177 | 5.56E-09 | 0.042897 | 0.0144 | 0.002934 |  |  |  |  |
| rs4369924 | A | G | A | G | 0.01362 | 0.00234 | 5.82E-09 | 0.044495 | 0.02 | 0.02582 |  |  |  |  |
| rs4442732 | G | A | G | A | -0.01063 | 0.00176 | 1.49E-09 | -0.0387 | 0.0142 | 0.006385 |  |  |  |  |
| rs56194430 | T | C | T | C | -0.01514 | 0.00232 | 6.65E-11 | -0.0424 | 0.0206 | 0.03928 |  |  |  |  |
| rs56330207 | A | G | A | G | 0.01184 | 0.00207 | 1.05E-08 | 0.035695 | 0.0169 | 0.03465 |  |  |  |  |
| rs61757207 | G | A | G | A | -0.04941 | 0.00795 | 5.09E-10 | 0.145697 | 0.0615 | 0.0178 |  |  |  |  |
| rs635754 | A | G | A | G | 0.0134 | 0.00173 | 8.01E-15 | 0.055596 | 0.0141 | 7.81E-05 |  |  |  |  |
| rs6731373 | G | A | G | A | 0.01256 | 0.00181 | 3.47E-12 | 0.041395 | 0.015 | 0.005796 |  |  |  |  |
| rs6774533 | T | C | T | C | 0.0126 | 0.00187 | 1.73E-11 | 0.0766 | 0.0157 | 1.08E-06 |  |  |  |  |
| rs6821231 | C | T | C | T | 0.01217 | 0.00197 | 6.06E-10 | 0.038803 | 0.0163 | 0.0175 |  |  |  |  |
| rs7117878 | A | C | A | C | -0.01036 | 0.00182 | 1.18E-08 | 0.033203 | 0.0148 | 0.02471 |  |  |  |  |
| rs71415374 | T | C | T | C | 0.02142 | 0.00307 | 2.93E-12 | 0.0569 | 0.024 | 0.01766 |  |  |  |  |
| rs72486027 | C | T | C | T | 0.01123 | 0.00197 | 1.25E-08 | 0.033195 | 0.0159 | 0.03706 |  |  |  |  |
| rs72828517 | C | T | C | T | 0.01836 | 0.00224 | 2.83E-16 | 0.061195 | 0.0183 | 0.000834 |  |  |  |  |
| rs72972965 | C | A | C | A | -0.01015 | 0.00183 | 2.84E-08 | -0.0523 | 0.0143 | 0.000245 |  |  |  |  |
| rs730384 | G | A | G | A | -0.01016 | 0.00171 | 3.01E-09 | 0.039001 | 0.0141 | 0.005558 |  |  |  |  |
| rs736282 | C | T | C | T | -0.01082 | 0.0017 | 2.07E-10 | -0.0454 | 0.0139 | 0.001094 |  |  |  |  |
| rs74545339 | A | G | A | G | -0.01626 | 0.00269 | 1.44E-09 | -0.0399 | 0.0219 | 0.06931 |  |  |  |  |
| rs74998289 | T | G | T | G | 0.01821 | 0.00213 | 1.31E-17 | -0.077 | 0.0172 | 7.23E-06 |  |  |  |  |
| rs76076331 | C | T | C | T | -0.01873 | 0.00248 | 4.40E-14 | 0.065499 | 0.0204 | 0.001303 |  |  |  |  |
| rs7625428 | C | T | C | T | -0.00989 | 0.00174 | 1.26E-08 | -0.0351 | 0.0143 | 0.0141 |  |  |  |  |
| rs7796203 | G | A | G | A | 0.01074 | 0.00171 | 3.60E-10 | 0.033298 | 0.0141 | 0.01819 |  |  |  |  |
| rs78721320 | G | A | G | A | -0.01307 | 0.00219 | 2.28E-09 | 0.054805 | 0.0185 | 0.003094 |  |  |  |  |
| rs8020034 | G | A | G | A | -0.01782 | 0.00223 | 1.17E-15 | -0.05 | 0.0181 | 0.005843 |  |  |  |  |
| rs837080 | C | T | C | T | 0.01092 | 0.0017 | 1.43E-10 | 0.035596 | 0.0139 | 0.01046 |  |  |  |  |
| rs9371881 | G | A | G | A | -0.01036 | 0.00178 | 5.76E-09 | -0.0346 | 0.0145 | 0.01722 |  |  |  |  |
| rs9386319 | G | A | G | A | 0.00991 | 0.00174 | 1.27E-08 | -0.0322 | 0.0142 | 0.02318 |  |  |  |  |
| rs9386787 | A | G | A | G | -0.00958 | 0.0017 | 1.82E-08 | -0.0301 | 0.0138 | 0.02975 |  |  |  |  |
| rs9557378 | A | G | A | G | -0.01094 | 0.00193 | 1.45E-08 | -0.0296 | 0.0156 | 0.05817 |  |  |  |  |
| rs9882532 | C | T | C | T | -0.01208 | 0.00177 | 8.17E-12 | 0.052705 | 0.0143 | 0.000232 |  |  |  |  |
| rs10266047 | G | C | G | C | 0.01132 | 0.00171 | 3.27E-11 | 0.017696 | 0.0139 | 0.2032 | *Removed during harmonisation | | | |
| rs10417097 | G | C | G | C | 0.01014 | 0.00174 | 5.17E-09 | -0.0211 | 0.0144 | 0.1426 |  |  |  |  |
| rs10772644 | G | C | G | C | -0.01614 | 0.00267 | 1.50E-09 | -0.0184 | 0.0216 | 0.395 |  |  |  |  |
| rs10773002 | T | A | T | A | -0.02191 | 0.00197 | 8.68E-29 | 0.008698 | 0.0161 | 0.588 |  |  |  |  |
| rs10862376 | T | A | T | A | -0.01616 | 0.00239 | 1.40E-11 | 0.012002 | 0.0196 | 0.5393 |  |  |  |  |
| rs10875121 | G | C | G | C | -0.01834 | 0.00226 | 5.53E-16 | -0.0367 | 0.0186 | 0.04853 |  |  |  |  |
| rs10963297 | G | C | G | C | 0.01904 | 0.00198 | 7.36E-22 | 0.006904 | 0.0163 | 0.6709 |  |  |  |  |
| rs11082975 | G | C | G | C | 0.01309 | 0.00172 | 2.52E-14 | -0.0247 | 0.0139 | 0.07638 |  |  |  |  |
| rs112806496 | G | C | G | C | 0.0187 | 0.00305 | 8.28E-10 | -0.0059 | 0.0263 | 0.8236 |  |  |  |  |
| rs112969166 | G | C | G | C | -0.01008 | 0.00171 | 3.81E-09 | 0.004701 | 0.0138 | 0.7362 |  |  |  |  |
| rs115000530 | T | A | T | A | 0.02892 | 0.00381 | 3.30E-14 | -0.0197 | 0.0323 | 0.5419 |  |  |  |  |
| rs11609711 | C | G | C | G | 0.01371 | 0.00241 | 1.35E-08 | 0.010396 | 0.0204 | 0.6097 |  |  |  |  |
| rs11708375 | G | C | G | C | 0.01376 | 0.00235 | 4.58E-09 | -0.0114 | 0.0201 | 0.5697 |  |  |  |  |
| rs117799466 | G | C | G | C | -0.01173 | 0.00198 | 2.91E-09 | -0.0184 | 0.0156 | 0.239 |  |  |  |  |
| rs12118513 | T | A | T | A | 0.01215 | 0.0021 | 7.47E-09 | -0.0238 | 0.0177 | 0.1791 |  |  |  |  |
| rs12332731 | A | T | A | T | 0.01374 | 0.00218 | 3.12E-10 | -0.0201 | 0.018 | 0.2632 |  |  |  |  |
| rs1245829 | A | T | A | T | -0.01083 | 0.00173 | 3.51E-10 | 0.021996 | 0.0141 | 0.1171 |  |  |  |  |
| rs12764593 | C | G | C | G | 0.02293 | 0.00358 | 1.53E-10 | -0.0176 | 0.0311 | 0.5712 |  |  |  |  |
| rs13130765 | C | G | C | G | -0.01014 | 0.00173 | 4.69E-09 | -0.005 | 0.0144 | 0.7293 |  |  |  |  |
| rs140711597 | C | G | C | G | 0.04026 | 0.0063 | 1.66E-10 | -0.0141 | 0.067 | 0.8338 |  |  |  |  |
| rs1408284 | C | G | C | G | -0.01388 | 0.0025 | 2.74E-08 | -0.0314 | 0.0207 | 0.1291 |  |  |  |  |
| rs1455350 | T | A | T | A | 0.01614 | 0.0017 | 2.61E-21 | 0.035503 | 0.0139 | 0.01078 |  |  |  |  |
| rs1592757 | C | G | C | G | -0.01045 | 0.0018 | 5.89E-09 | 0.042302 | 0.0145 | 0.003557 |  |  |  |  |
| rs1689510 | G | C | G | C | -0.01761 | 0.0018 | 1.40E-22 | -0.0146 | 0.0148 | 0.324 |  |  |  |  |
| rs17428076 | C | G | C | G | 0.01216 | 0.00198 | 8.90E-10 | 0.032303 | 0.0161 | 0.04545 |  |  |  |  |
| rs175325 | A | T | A | T | -0.01179 | 0.00174 | 1.11E-11 | -0.0037 | 0.0141 | 0.7929 |  |  |  |  |
| rs17568389 | A | T | A | T | 0.01201 | 0.0017 | 1.53E-12 | -0.0113 | 0.0138 | 0.414 |  |  |  |  |
| rs1960603 | C | G | C | G | 0.01096 | 0.00198 | 3.07E-08 | -0.0005 | 0.0161 | 0.9744 |  |  |  |  |
| rs2414072 | A | T | A | T | -0.01005 | 0.00171 | 4.35E-09 | -0.0055 | 0.0139 | 0.6906 |  |  |  |  |
| rs2478208 | C | G | C | G | -0.0106 | 0.0017 | 4.82E-10 | -0.0266 | 0.0139 | 0.05647 |  |  |  |  |
| rs2545798 | T | A | T | A | 0.01346 | 0.00171 | 3.11E-15 | 0.003998 | 0.0143 | 0.7784 |  |  |  |  |
| rs28373063 | G | C | G | C | -0.01389 | 0.00229 | 1.23E-09 | 0.013501 | 0.0193 | 0.4827 |  |  |  |  |
| rs2923431 | C | G | C | G | 0.0114 | 0.00176 | 9.84E-11 | -0.0109 | 0.0142 | 0.4443 |  |  |  |  |
| rs34410 | G | C | G | C | 0.00931 | 0.0017 | 4.70E-08 | 0.011901 | 0.0139 | 0.3903 |  |  |  |  |
| rs35518360 | A | T | A | T | 0.01875 | 0.00327 | 9.79E-09 | -0.047 | 0.029 | 0.1046 |  |  |  |  |
| rs35532491 | T | A | T | A | 0.02007 | 0.00286 | 2.42E-12 | 0.021796 | 0.026 | 0.4018 |  |  |  |  |
| rs3747631 | G | C | G | C | -0.02207 | 0.00208 | 2.97E-26 | 0 | 0.0169 | 0.9997 |  |  |  |  |
| rs3768480 | G | C | G | C | -0.01038 | 0.00172 | 1.74E-09 | -0.01 | 0.0141 | 0.4761 |  |  |  |  |
| rs3800546 | G | C | G | C | -0.01183 | 0.00194 | 9.73E-10 | -0.04 | 0.0159 | 0.01201 |  |  |  |  |
| rs401687 | G | C | G | C | -0.01144 | 0.0017 | 1.86E-11 | -0.0258 | 0.0138 | 0.06269 |  |  |  |  |
| rs406413 | A | T | A | T | 0.01695 | 0.00209 | 4.84E-16 | 0.069097 | 0.0168 | 4.00E-05 |  |  |  |  |
| rs4728278 | C | G | C | G | 0.01066 | 0.00184 | 6.38E-09 | 0.002497 | 0.0152 | 0.8689 |  |  |  |  |
| rs4733264 | C | G | C | G | -0.00954 | 0.00174 | 4.53E-08 | 0.003803 | 0.0141 | 0.7866 |  |  |  |  |
| rs4757957 | C | G | C | G | 0.0141 | 0.00184 | 1.81E-14 | 0.001 | 0.0151 | 0.9448 |  |  |  |  |
| rs4766424 | G | C | G | C | -0.0141 | 0.00258 | 4.43E-08 | -0.0184 | 0.021 | 0.3797 |  |  |  |  |
| rs4877516 | T | A | T | A | 0.01163 | 0.00171 | 1.02E-11 | -0.0098 | 0.014 | 0.4819 |  |  |  |  |
| rs4984682 | G | C | G | C | 0.01202 | 0.00203 | 3.13E-09 | 0.008899 | 0.0167 | 0.5931 |  |  |  |  |
| rs55736314 | C | G | C | G | -0.01431 | 0.00174 | 1.63E-16 | 0.035396 | 0.0143 | 0.0133 |  |  |  |  |
| rs55771711 | C | G | C | G | 0.01555 | 0.00199 | 5.41E-15 | 0.065797 | 0.0162 | 4.70E-05 |  |  |  |  |
| rs59480703 | G | C | G | C | 0.01237 | 0.00215 | 8.32E-09 | 0.026796 | 0.0175 | 0.1265 |  |  |  |  |
| rs59953820 | A | T | A | T | -0.01595 | 0.00289 | 3.50E-08 | -0.0021 | 0.024 | 0.9316 |  |  |  |  |
| rs60483752 | G | C | G | C | -0.01078 | 0.00172 | 3.89E-10 | -0.0162 | 0.0143 | 0.2579 |  |  |  |  |
| rs62247449 | C | G | C | G | 0.01137 | 0.00171 | 3.31E-11 | 0.025405 | 0.0141 | 0.07133 |  |  |  |  |
| rs663234 | G | C | G | C | -0.01005 | 0.00174 | 7.39E-09 | -0.0137 | 0.0142 | 0.3332 |  |  |  |  |
| rs6678474 | T | A | T | A | -0.02927 | 0.00513 | 1.13E-08 | 0.055502 | 0.0443 | 0.2104 |  |  |  |  |
| rs6867851 | C | G | C | G | -0.012 | 0.00173 | 3.97E-12 | -0.0159 | 0.0144 | 0.2677 |  |  |  |  |
| rs7016302 | G | C | G | C | 0.01243 | 0.00228 | 4.98E-08 | 0.0298 | 0.0186 | 0.1086 |  |  |  |  |
| rs7278859 | A | T | A | T | -0.01013 | 0.00185 | 4.15E-08 | -0.0114 | 0.0149 | 0.4419 |  |  |  |  |
| rs73496688 | T | A | T | A | -0.01429 | 0.00238 | 2.04E-09 | -0.051 | 0.0196 | 0.009105 |  |  |  |  |
| rs76878669 | C | G | C | G | 0.01399 | 0.00205 | 8.67E-12 | 0.023697 | 0.0163 | 0.1444 |  |  |  |  |
| rs7833201 | C | G | C | G | -0.01532 | 0.00262 | 5.05E-09 | 0.001099 | 0.0224 | 0.9597 |  |  |  |  |
| rs7920624 | T | A | T | A | -0.01181 | 0.0017 | 3.97E-12 | -0.0156 | 0.0138 | 0.2577 |  |  |  |  |
| rs7928622 | T | A | T | A | 0.01011 | 0.00181 | 2.52E-08 | 0.019101 | 0.0148 | 0.1985 |  |  |  |  |
| rs925161 | G | C | G | C | 0.00941 | 0.0017 | 3.26E-08 | 0.013004 | 0.0139 | 0.3496 |  |  |  |  |
| rs9529119 | C | G | C | G | 0.01295 | 0.00204 | 2.13E-10 | -0.0011 | 0.0171 | 0.9477 |  |  |  |  |
| rs969512 | T | A | T | A | 0.01249 | 0.00179 | 3.22E-12 | 0.013197 | 0.0145 | 0.3652 |  |  |  |  |
| rs9886703 | A | T | A | T | -0.01315 | 0.00227 | 7.14E-09 | 0.025697 | 0.0191 | 0.1788 |  |  |  |  |
| rs9938678 | A | T | A | T | -0.01355 | 0.00205 | 4.12E-11 | -0.01 | 0.0171 | 0.5599 |  |  |  |  |

**Table S3**. Causal effect estimates of ADHD genetic liability on educational attainment derived from two-sample MR analyses.

| **Method** | **N.SNP** | **Beta** | **SE** | **P** | **95% CI** | |
| --- | --- | --- | --- | --- | --- | --- |
| Inverse variance weighted | 8 | -0.10265 | 0.022538 | 5.25E-06 | -0.14683 | -0.05848 |
|  |  |  |  |  |  |  |
| MR Egger | 8 | -0.01291 | 0.102849 | 0.904191 | -0.2145 | 0.188672 |
| IGX= 0.5; Suggesting a 50% attenuation of the MR Egger causal effect estimate towards zero | | | | | | |
| MR Egger Simex | 8 | -0.015872 | 0.138333 | 0.912 | -0.287 | 0.2552607 |
| Weighted median | 8 | -0.07982 | 0.015599 | 3.10E-07 | -0.1104 | -0.04925 |
| Weighted mode | 8 | -0.0809 | 0.025975 | 0.016975 | -0.13181 | -0.02999 |

**Table S4**. Direct causal effect estimates of genetic liability to ADHD and cognitive ability on educational attainment, as estimated by MVMR analysis.

|  | **Beta** | **95% CI** | | **Conditional**  **F-statistic** | **SE** | **P** |
| --- | --- | --- | --- | --- | --- | --- |
| Direct effect: ADHD | -0.04924 | -0.07579 | -0.02269 | 3 | 0.013547 | 0.000363 |
| Direct effect: CA | 0.392492 | 0.354442 | 0.430541 | 9 | 0.019413 | 3.40E-48 |
| Robust to weak instruments direct effect: ADHD | -0.02241 | -0.063 | 0.035 |  | | |

**Table S5.** Causal effect estimates of ASD genetic liability on educational attainment derived from two-sample MR analyses.

| **Method** | **N.SNP** | **Beta** | **SE** | **P** | **95% CI** | |
| --- | --- | --- | --- | --- | --- | --- |
| Inverse variance weighted | 7 | 0.003949 | 0.031207 | 0.899301 | -0.05722 | 0.065115 |
|  |  |  |  |  |  |  |
| MR Egger | 7 | 0.103776 | 0.119219 | 0.42386 | -0.12989 | 0.337446 |
| IGX= 0.63; Suggesting a 37% attenuation of the MR Egger causal effect estimate towards zero | | | | | | |
| MR Egger Simex | 7 | 0.13627 | 0.15449 | 0.418 | -0.16653 | 0.4390704 |
| Weighted median | 7 | -0.01166 | 0.013816 | 0.398748 | -0.03874 | 0.015421 |
| Weighted mode | 7 | -0.01768 | 0.01428 | 0.261933 | -0.04567 | 0.010309 |
| MR Raps | 7 | 0.030946 | 0.029614 | 0.296022 | -0.0271 | 0.088989 |

**Table S6**. Direct causal effect estimates of genetic liability to ASD and cognitive ability on educational attainment, as estimated by MVMR analysis.

|  | **Beta** | **95% CI** | | **Conditional**  **F-statistic** | **SE** | **P** |
| --- | --- | --- | --- | --- | --- | --- |
| Direct effect: ASD | 0.028262 | 0.002263 | 0.054262 | 3 | 0.013265 | 0.03443 |
| Direct effect: CA | 0.42195 | 0.386934 | 0.456966 | 31 | 0.017865 | 5.09E-58 |
| Robust to weak instruments direct effect: ASD | 0.04364388 | 0.017 | 0.069 |  | | |

**Table S7a.** Causal effect estimates of genetic liability to higher educational attainment on risk of ADHD derived from two-sample MR analyses.

| **Method** | **N.SNP** | **OR** | **SE** | **P** | **95% CI** | |
| --- | --- | --- | --- | --- | --- | --- |
| Inverse variance weighted | 400 | 0.304211 | 0.079272 | 6.13E-51 | 0.260434 | 0.355348 |
|  |  |  |  |  |  |  |
| MR Egger | 400 | 0.381112 | 0.322415 | 0.002944 | 0.202585 | 0.716964 |
| IGX= 0.63; Suggesting a 37% attenuation of the MR Egger causal effect estimate towards zero. | | | | | | |
| MR Egger Simex | 400 | 0.2721037 | 0.425583 | 0.00238 | 0.1181601 | 0.626611 |
| Weighted median | 400 | 0.318067 | 0.096573 | 1.88E-32 | 0.263217 | 0.384347 |
| Weighted mode | 400 | 0.462979 | 0.405596 | 0.058336 | 0.209079 | 1.025208 |

**Table S7b.** Causal effect estimates of genetic liability to higher educational attainment on risk of ADHD after removing instruments identified to explain more variation in the outcome through Steiger filtering.

| **Method** | **N.SNP** | **OR** | **SE** | **P** | **95% CI** | |
| --- | --- | --- | --- | --- | --- | --- |
| Inverse variance weighted | 318* | 0.49566 | 0.066515 | 4.97E-26 | 0.435076 | 0.56468 |
|  |  |  |  |  |  |  |
| MR Egger | 318 | 0.343599 | 0.260931 | 5.39E-05 | 0.206036 | 0.573007 |
| IGX= 0.67; Suggesting a 33% attenuation of the MR Egger causal effect estimate towards zero. | | | | | | |
| MR Egger Simex | 318 | 0.237563 | 3.36E-01 | 2.46E-05 | 0.123042 | 0.458674 |
| Weighted median | 318 | 0.465216 | 0.098438 | 7.60E-15 | 0.383587 | 0.564218 |
| Weighted mode | 318 | 0.484627 | 0.409337 | 7.77E-02 | 0.217257 | 1.08104 |

*EAF was not provided for the ADHD SNPs and therefore was approximated using phase3 european ancestry 1000Genomes reference panel. One SNP was not identified in the panel and therefore removed from the analysis.

**Table S8**. Direct causal effect estimates of genetic liability to higher EA and cognitive ability on risk of ADHD, as estimated by MVMR analysis.

|  | **OR** | **95% CI** | | **Conditional F-statistic** | **SE** | **P** |
| --- | --- | --- | --- | --- | --- | --- |
| Direct effect: EA | 0.333641 | 0.260406 | 0.427472 | 7 | 0.12644 | 6.13E-17 |
| Direct effect: EA- SNPs from Steiger excluded | 0.474302 | 0.355872 | 0.632142 | 2 | 0.146567 | 6.45E-07 |
| Direct effect: CA | 0.832399 | 0.670293 | 1.033708 | 6 | 0.110508 | 0.097568 |
| Robust to weak instruments direct effect: EA | 0.3834937 | 0.299692 | 0.5005739 |  | | |

**Table S9a.** Causal effect estimates of genetic liability to higher educational attainment on risk of ASD derived from two-sample MR analyses.

| **Method** | **N.SNP** | **OR** | **SE** | **P** | **95% CI** | |
| --- | --- | --- | --- | --- | --- | --- |
| Inverse variance weighted | 410 | 1.510136 | 0.081609 | 4.40E-07 | 1.286914 | 1.772077 |
|  |  |  |  |  |  |  |
| MR Egger | 410 | 2.519884 | 0.33014 | 0.005362 | 1.319349 | 4.81284 |
| IGX= 0.64; Suggesting a 36% attenuation of the MR Egger causal effect estimate towards zero. | | | | | | |
| MR Egger Simex | 410 | 3.564544 | 0.429477 | 0.00326 | 1.536122 | 8.271459 |
| Weighted median | 410 | 1.432918 | 0.097467 | 0.000224 | 1.18374 | 1.734549 |
| Weighted mode | 410 | 1.335246 | 0.297617 | 0.331907 | 0.745116 | 2.392755 |

**Table S9b.** Causal effect estimates of genetic liability to higher educational attainment on risk of ASD after removing instruments identified to explain more variation in the outcome through Steiger filtering.

| **Method** | **N.SNP** | **OR** | **SE** | **P** | **95% CI** | |
| --- | --- | --- | --- | --- | --- | --- |
| Inverse variance weighted | 347* | 1.306284 | 0.06507 | 4.02E-05 | 1.14987 | 1.483974 |
|  |  |  |  |  |  |  |
| MR Egger | 347 | 2.741672 | 0.265259 | 1.70E-04 | 1.63013 | 4.611144 |
| IGX= 0.64; Suggesting a 33% attenuation of the MR Egger causal effect estimate towards zero. | | | | | | |
| MR Egger Simex | 347 | 4.015063 | 3.37E-01 | 4.67E-05 | 2.073811 | 7.773481 |
| Weighted median | 347 | 1.361438 | 0.098718 | 1.78E-03 | 1.121935 | 1.652068 |
| Weighted mode | 347 | 1.304833 | 0.372311 | 4.75E-01 | 0.628981 | 2.706901 |

*EAF was not provided for the ASD SNPs and therefore was approximated using phase3 european ancestry 1000Genomes reference panel. One SNP was not identified in the panel and therefore removed from the analysis.

**Table S10**. Direct causal effect estimates of genetic liability to higher EA and cognitive ability on risk of ASD, as estimated by MVMR analysis.

|  | **OR** | **95% CI** | | **Conditional**  **F-statistic** | **SE** | **P** |
| --- | --- | --- | --- | --- | --- | --- |
| Direct effect: EA | 1.243268 | 0.963451 | 1.604354 | 7 | 0.130091 | 0.094807 |
| Direct effect: EA- SNPs from Steiger excluded | 1.172924 | 0.883974 | 1.556326 | 3 | 0.1443 | 0.27 |
| Direct effect: CA | 1.256709 | 1.006175 | 1.569625 | 6 | 0.113439 | 0.044524 |
| Robust to weak instruments direct effect: EA | 1.378239 | 0.8904752 | 1.847808 |  | | |

References

1. Bowden, J., Davey Smith, G. & Burgess, S. Mendelian randomization with invalid instruments: effect estimation and bias detection through Egger regression. *Int. J. Epidemiol.* **44**, 512–525 (2015).

2. Pierce, B. L. & Burgess, S. Efficient design for Mendelian randomization studies: subsample and 2-sample instrumental variable estimators. *Am. J. Epidemiol.* **178**, 1177–1184 (2013).

3. Higgins, J. P. T., Thompson, S. G., Deeks, J. J. & Altman, D. G. Measuring inconsistency in meta-analyses. *Bmj* **327**, 557–560 (2003).

4. Bowden, J. *et al.* Assessing the suitability of summary data for two-sample Mendelian randomization analyses using MR-Egger regression: the role of the I 2 statistic. *Int. J. Epidemiol.* **45**, 1961–1974 (2016).

5. Bowden, J., Davey Smith, G., Haycock, P. C. & Burgess, S. Consistent estimation in Mendelian randomization with some invalid instruments using a weighted median estimator. *Genet. Epidemiol.* **40**, 304–314 (2016).

6. Hartwig, F. P., Davey Smith, G. & Bowden, J. Robust inference in summary data Mendelian randomization via the zero modal pleiotropy assumption. *Int. J. Epidemiol.* **46**, 1985–1998 (2017).

7. Zhao, Q., Wang, J., Hemani, G., Bowden, J. & Small, D. S. Statistical inference in two-sample summary-data Mendelian randomization using robust adjusted profile score. *Ann. Stat.* **48**, 1742–1769 (2020).

8. Hemani, G., Tilling, K. & Smith, G. D. Orienting the causal relationship between imprecisely measured traits using GWAS summary data. *PLoS Genet.* **13**, e1007081 (2017).
